# Supplementary material for: Effectiveness of a fully immersive virtual reality-based therapeutic exercise programme with altered visual feedback in patients with fibromyalgia: A study protocol for a randomised controlled trial
Source: PLoS One. 2026 Jun 4;21(6):e0348346. doi: 10.1371/journal.pone.0348346 (PMC13235889; doi:10.1371/journal.pone.0348346)
Supplement: S1 Protocol — (DOCX) [file pone.0348346.s002.docx]

**Memoria Proyecto**

**Comité de Ética de Investigación con** **Humanos**

**(Área Biosanitaria/Área Humanidades-Sociales)**

Título: Eficacia de la realidad virtual inmersiva en pacientes con fibromialgia. Un ensayo clínico aleatorizado.

Nombre de Tutor TFG/TFM/PI/Tesis: Dr. Juan José Amer Cuenca y Dr. Juan Francisco Lisón Párraga.

Nombre del estudiante: Carlos Salvador Huerta

**1.Resumen (200 palabras)**

La fibromialgia (FM) es un síndrome caracterizado por dolor generalizado crónico, fatiga y alteraciones del sueño. Su tratamiento incluye intervenciones farmacológicas, aunque su efectividad es limitada debido a efectos adversos y baja adherencia. En contraste, el ejercicio físico ha demostrado beneficios en la reducción del dolor y la mejora funcional. Sin embargo, el dolor persistente en FM dificulta la adherencia al ejercicio, lo que motiva la búsqueda de estrategias complementarias.

La realidad virtual inmersiva (RVI) ha surgido como una herramienta prometedora para modular la percepción del dolor mediante inmersión sensorial y distracción cognitiva. Aunque se ha utilizado en FM a través de *exergames* (videojuegos interactivos que buscan hacer de la actividad física algo gratificante), su integración simultánea con el ejercicio terapéutico no ha sido ampliamente explorada. Se ha planteado que la inmersión en entornos virtuales puede influir en la percepción del esfuerzo y la amplitud del movimiento, optimizando los beneficios del ejercicio.

Este estudio evaluará la eficacia de un sistema de RVI que ajusta el feedback visual del paciente, generando la ilusión de menor movimiento corporal durante el ejercicio de resistencia. Se espera que esta manipulación favorezca un mayor rango de movimiento sin aumentar la percepción de dolor o esfuerzo, optimizando así la respuesta al tratamiento en pacientes con FM.

**2. Introducción y justificación del proyecto**

La fibromialgia (FM) es una enfermedad etiología desconocida, caracterizada principalmente por la presencia de dolor crónico (>3 meses) y generalizado, que afecta diversas regiones del cuerpo ^1^. Aunque el dolor generalizado es el rasgo clínico más distintivo, es una enfermedad compleja y polisintomática que abarca otros síntomas cardinales, como la fatiga y las alteraciones del sueño^2–4^.

Además, esta condición suele estar acompañada de disfunciones cognitivas ^3^; síndromes de dolor regional^5–9^; alteraciones autonómicas ^10–16^; síntomas psiquiátricos^17–19^ e hipersensibilidad a estímulos externos. La sintomatología queda resumida en la *figura 1.*


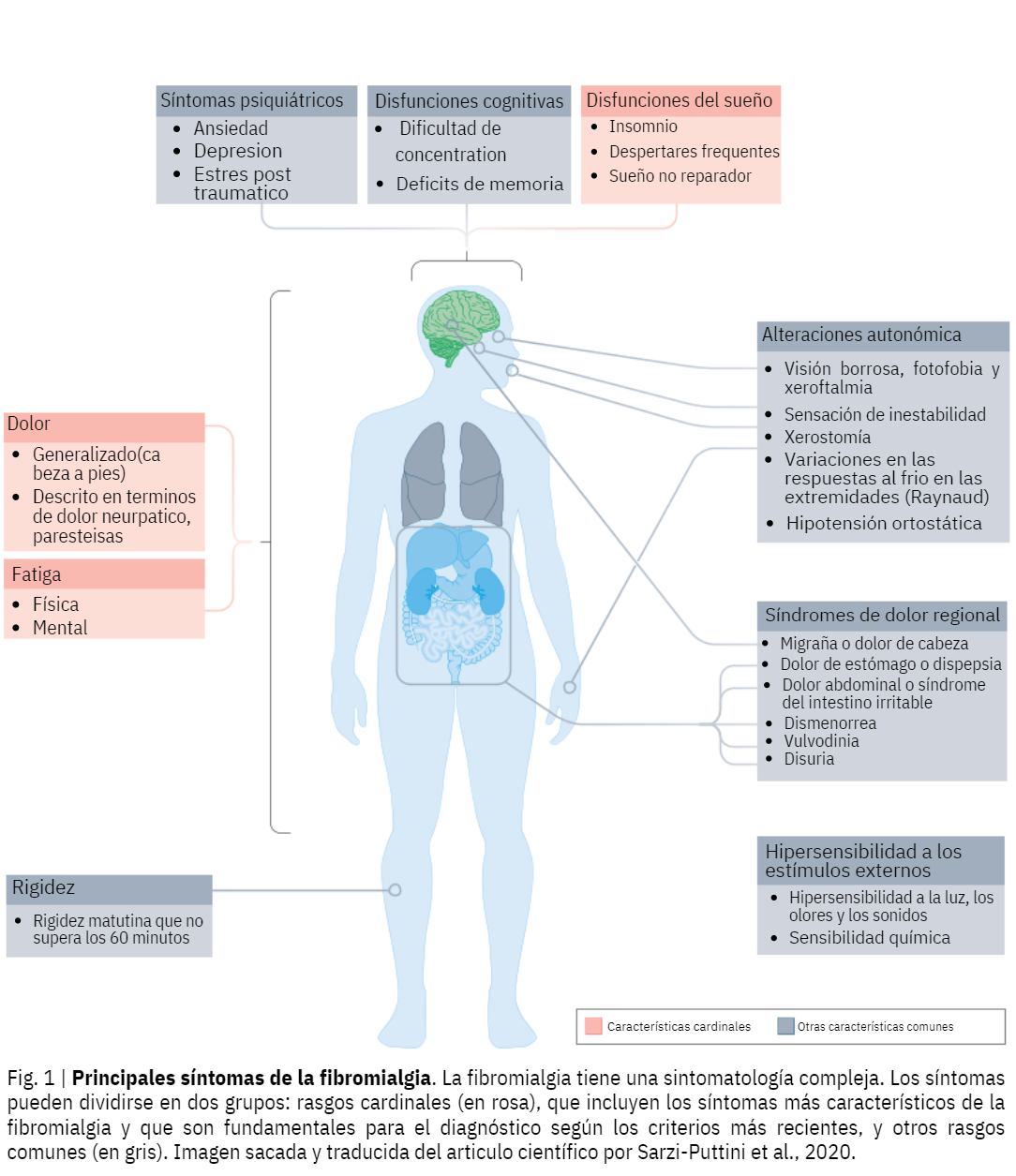


Su prevalencia varía ampliamente en función de los criterios diagnósticos empleados. Según los criterios del ACR de 1990, las tasas oscilan entre el 0,4 % y el 8,8 %, con una media global estimada del 2,7 %. En Europa, un estudio multinacional realizado en Francia, Portugal, España, Alemania e Italia reportó una prevalencia del 4,7 %, con una relación mujeres:hombres de 3:1^20^. Reconocida como la tercera enfermedad musculoesquelética más prevalente, después del dolor lumbar y la artrosis, su frecuencia aumenta con la edad, alcanzando su máximo entre los 50 y 60 años^21,22^. No obstante, la precisión de estas estimaciones puede verse afectada por discrepancias entre los datos reportados por los pacientes y los diagnósticos clínicos, debido a un reconocimiento insuficiente de la enfermedad en la práctica médica^23^.

La calidad de vida de los sujetos que padecen FM se ve gravemente afectada por las características de los síntomas propios de la enfermedad, lo cual se refleja en los elevados costes de atención sanitaria. El número anual de consultas médicas necesarias es casi el doble que el de las personas sanas, y los costos totales de atención sanitaria son aproximadamente tres veces más altos^24,25^. Además, los costos indirectos para la sociedad son significativos, principalmente debido a la pérdida de productividad laboral^26,27^: un estudio mostró que el 24,3 % de los pacientes dejó de trabajar cinco años después del inicio de la FM^28^.

Se ha establecido una relación directa entre los costes sanitarios y la gravedad de los síntomas de la FM ^29^, lo que resalta la necesidad de enfoques terapéuticos eficaces y sostenibles. Actualmente, el tratamiento de la FM incluye intervenciones farmacológicas que, si bien pueden proporcionar alivio sintomático, presentan una alta incidencia de efectos secundarios y una menor adherencia por parte de los pacientes ^30^.

En contraste, el ejercicio físico (EF) se ha consolidado como una estrategia terapéutica eficaz y bien documentada en el manejo de la FM. Múltiples estudios han demostrado sus beneficios en la reducción del dolor, la mejora de la calidad de vida, el aumento de la capacidad física y la reducción de síntomas depresivos. Entre las modalidades más efectivas se encuentran tanto el entrenamiento de resistencia ^31–36^ como el ejercicio aeróbico^31,32,37–39^, los cuales han sido ampliamente adoptados en las recomendaciones clínicas para el tratamiento de la FM.

Sin embargo, dada la naturaleza compleja y multidimensional del dolor crónico, se han explorado nuevas estrategias complementarias al propio ejercicio. En este contexto, la realidad virtual (RV) ha surgido como una alternativa innovadora con un gran potencial terapéutico. Estudios recientes sugieren que la RV puede modular la percepción del dolor a través de la inmersión sensorial, distracción cognitiva y estimulación multisensorial, lo que la posiciona como una opción prometedora para mejorar el control del dolor en pacientes con FM^40,41^.

Las tecnologías de RV pueden clasificarse en tres categorías según su nivel de inmersión: no inmersivas, semi-inmersivas y totalmente inmersivas. El nivel de inmersión influye directamente en la experiencia perceptual y psicológica del usuario, así como en su sensación de *“embodiment”* (encarnación con el avatar) en el entorno virtual^42^.

Actualmente, en el tratamiento de la FM, se han empleado mayoritariamente dispositivos de RV no inmersivos clasificados como *exergames*. Son opciones que ofrecen un nivel de inmersión limitado. A pesar de ello han demostrado ser eficaces en la mejora de variables de interés en la población estudiada como el impacto de los síntomas de la FM, el dolor, el equilibrio dinámico, la capacidad aeróbica, la fatiga, la calidad de vida, la ansiedad y la depresión^43–53^.

La realidad virtual inmersiva (RVI) proporciona una experiencia de inmersión significativamente superior^42,54^. De esta manera son capaces no solo de mejorar la sensación de “Embodiment” sino que además ofrecen la posibilidad de proporcionar una retroalimentación al usuario^55^. Estudios sugieren que la calidad de la inmersión está directamente relacionada con la efectividad de la distracción del dolor, de modo que niveles más altos de inmersión pueden generar una mayor reducción del dolor en comparación con niveles más bajos^56^.

La realidad virtual inmersiva (RVI) ha emergido como una herramienta terapéutica innovadora, no solo para modular la percepción del dolor, sino también para mejorar la funcionalidad en pacientes con fibromialgia (FM)^57–59^. Si bien su uso ha sido ampliamente estudiado en el contexto de exergames y como complemento posterior al ejercicio, su impacto cuando se aplica de forma simultánea al ejercicio no ha sido suficientemente investigado. Además, la modificación del feedback visual en RVI durante un programa de ejercicio terapéutico y sus efectos en pacientes con FM aún no han sido evaluados de manera exhaustiva.

Con todo ello, y hasta donde sabemos, en la literatura no se encuentran antecedentes del estudio de los efectos sobre los síntomas de la fibromialgia de un programa de ejercicios terapéuticos realizados con realidad virtual inmersiva modificando el feedback visual del paciente.

En este sentido, el presente estudio tiene como objetivo evaluar la eficacia de un sistema de RVI que ajusta el feedback visual del paciente, generando la ilusión de un movimiento corporal reducido en comparación con el movimiento real, mientras se realiza un entrenamiento de resistencia. Este mecanismo podría favorecer un mayor rango de movimiento sin incrementar la percepción de esfuerzo o dolor, optimizando así la eficacia del tratamiento.

**3. Objetivos**

Objetivo 1: Evaluar la efectividad de la realidad virtual inmersiva combinada con ejercicios de resistencia para mejorar el dolor producido por la fibromialgia en la vida diaria de los pacientes.

Objetivo 2: Determinar la capacidad de la realidad virtual inmersiva combinada con ejercicios de resistencia para mejorar la calidad de vida y capacidad funcional de los pacientes con fibromialgia.

Objetivo 3: Analizar la efectividad de la realidad virtual inmersiva combinada con ejercicios de resistencia para producir cambios en el grado de sensibilización central de los pacientes con fibromialgia.

**4. Metodología:**

4.1 Diseño del estudio.

Este Ensayo Clínico Aleatorizado (ECA) ha sido diseñado para evaluar la eficacia de la realidad virtual inmersiva en pacientes con fibromialgia. Se asignarán aleatoriamente los participantes a dos grupos. El grupo intervención recibirá ejercicio terapéutico combinado con realidad virtual inmersiva, mientras que el grupo control realizará únicamente el ejercicio de resistencia. El objetivo es determinar si la realidad virtual inmersiva es capaz de producir una mejora significativa en los resultados del grupo intervención con respecto al grupo control.

4.2 Población diana y población a estudiar.

Adultos de ambos sexos y mayores de 18 años que padezcan fibromialgia (diagnosticada mediante ACR 1990, 2010,2011 y 2016).

4.3 Criterios de selección.

Los criterios de selección aplicados en este estudio serán aquellos estandarizados y comúnmente utilizados en investigaciones donde la intervención consiste en la aplicación de ejercicio terapéutico y/o realidad virtual inmersiva en pacientes con fibromialgia.

Se incluirán participantes que cumplan los siguientes requisitos: adultos de ambos sexos, mayores de 18 años, con diagnóstico de fibromialgia según cualquiera de los criterios establecidos por el *American College of Rheumatology* (ACR 1990, 2010, 2011 o 2016), con capacidad de comunicación con el personal del estudio, que manifiesten su voluntad expresa de participación mediante la firma del consentimiento informado y que presenten un dolor percibido igual o superior a 3 en una Escala Numérica de 11 puntos (NRS-11).

Se excluirán aquellos participantes que presenten enfermedades adicionales y/o síntomas que constituyan una contraindicación para la intervención con realidad virtual inmersiva y ejercicio, patologías que puedan interferir en los resultados, tales como trastornos visuales, auditivos, perceptivos o sensoriales, uso de medicación que pueda afectar los resultados del estudio, seguimiento de un tratamiento de actividad física terapéutica durante la intervención o cambios en las terapias usuales, ya sea en la medicación o en las terapias físicas, a lo largo de la intervención.

4.4 Cálculo del tamaño muestral.

El resultado primario es el FIQR. La diferencia mínima clínicamente importante entre grupos es ≈14% del total del FIQR. Asumiendo DE=20, α=0,05 y potencia=80% con ANCOVA ajustada por basal, se requieren 32 por grupo (64 total). Con 25% de pérdidas, la muestra objetivo es 80.

4.5 Procedimiento de captación de participantes.

El reclutamiento de participantes se llevará a cabo en colaboración con la Asociación Valenciana de Afectados de Fibromialgia (AVAFI), que será la encargada de difundir la información pertinente a través de diversos medios con el objetivo de facilitar la captación de voluntarios. Además de la explicación detallada de la investigación, se destinará un tiempo específico para resolver dudas y atender consultas individuales de los asistentes. De esta manera, se garantizará que los posibles participantes tengan un conocimiento completo de la naturaleza del estudio y de la intervención antes de tomar una decisión informada sobre su participación.

4.6 Tipo de muestreo y aleatorización

El reclutamiento de participantes y la obtención del consentimiento informado por escrito serán realizados por médicos del grupo de investigación “Estilo de vida y salud” de la Universidad CEU Cardenal Herrera. La aleatorización será llevada a cabo por un investigador independiente mediante una secuencia 1:1 generada por ordenador, con bloques permutados estratificados de tamaños aleatorios (2–4) dentro de ocho estratos definidos por sexo (hombre/mujer) y gravedad de la FM (cuatro clústeres FIQR según Pérez-Aranda et al., 2019 [32]). La asignación se ocultará mediante sobres opacos, sellados y numerados de forma secuencial (SNOSE), preparados fuera del centro con precintos a prueba de manipulación y copias autocopiativas. Tras la evaluación basal, el personal del centro abrirá el siguiente sobre en orden numérico para asignar al grupo experimental (GE) o al grupo control (GC). La lista de aleatorización será custodiada por un gestor independiente de datos sin participación en el reclutamiento ni en la evaluación. Cualquier desviación de la secuencia o de la integridad de los sobres se registrará e investigará.

Los evaluadores de resultados y el estadístico del ensayo estarán cegados a la asignación. Los participantes y los proveedores de la intervención no podrán ser cegados debido a la naturaleza de la RV inmersiva y se les instruirá para no revelar su asignación durante las evaluaciones. Las evaluaciones serán realizadas por personal sin acceso a los registros de programación ni de asignación; los grupos se etiquetarán como A/B hasta el bloqueo de la base de datos. El descegamiento solo se realizará para abordar problemas graves de seguridad o desviaciones del protocolo con implicaciones de seguridad, previa autorización por escrito del investigador principal; todos los eventos de descegamiento quedarán registrados. La lista de asignación permanecerá en poder del gestor independiente de datos.

4.7 Tipo de cegamiento

Este estudio se llevará a cabo bajo un diseño de ciego único, en el que los evaluadores desconocerán la asignación de los participantes a los grupos de intervención. Esto permitirá minimizar el sesgo de detección, asegurando una evaluación objetiva de los resultados y aumentando la validez del estudio.

4.8 Intervención

El programa de intervención tendrá una duración total de seis semanas, con dos sesiones semanales de 60 minutos cada una. Las sesiones se realizarán en las instalaciones de AVAFI, en una sala climatizada acondicionada al efecto, y con las condiciones de espacio y privacidad necesarias. Cada sesión será dirigida por un investigador el cual se encargará de la correcta realización de los ejercicios, así como de garantizar el bienestar de estos. La sesión se dividirá en tres partes diferenciadas:

- **Fase de calentamiento (5 minutos):**

Ejercicios de movilidad articular combinados con la respiración para preparar el cuerpo para la actividad física. Los ejercicios se centrarán en la movilidad cervical, lumbopélvica, extremidades superiores e inferiores, sin carga adicional.

- **Fase de intervención principal (50 minutos):**

Los participantes llevarán a cabo un programa de ejercicios terapéuticos adaptado a su grupo (intervención o control), con la intensidad regulada mediante la Escala de Borg (6-20). Las sesiones serán supervisadas por un investigador para asegurar la correcta ejecución de los ejercicios y la seguridad de los participantes. Los ejercicios están ilustrados en la figura 2. Estos consisten en una serie de movimientos que incluyen flexión, extensión, rotación e inclinación del tronco, en diversas posiciones (de pie, sentado y tumbado). Durante la ejecución, se les instruirá a sincronizar las fases concéntrica y excéntrica con su ritmo respiratorio, con el objetivo de mantener una velocidad de ejecución uniforme.

El programa se centrará en ejercicios utilizando el peso corporal, y a partir de la sexta sesión, se añadirá pesas en las muñecas y algunos ejercicios con mancuernas. Los participantes realizarán entre 1 y 2 series de cada ejercicio, ajustando la intensidad y el número de repeticiones según su esfuerzo percibido, evaluado mediante la Escala de Borg (6-20), con un rango de intensidad adecuado entre los valores de 13 y 17. Durante cada sesión, se registrarán la intensidad del dolor NRS-11, el esfuerzo percibido (RPE) y el bienestar asociado a la Realidad Virtual.

- **Fase de enfriamiento (5 minutos):**

Ejercicios de estiramiento y respiración para reducir la tensión muscular y promover la relajación. Se incluirán estiramientos de la región lumbar y las extremidades inferiores, y ejercicios de respiración profunda en posición supina.

**Grupo de intervención: Realidad Virtual + Ejercicio**

Los participantes asignados a este grupo realizarán el programa de ejercicios terapéuticos anteriormente descrito con la utilización simultanea del dispositivo de Realidad Virtual inmersiva (HTC Vive Pro). La RV modificará la información visual y propioceptiva del paciente, lo que les permitirá reducir la percepción del movimiento mejorando la tolerancia al dolor y la conciencia corporal.

**Grupo control: Ejercicio sin Realidad Virtual**

Los participantes en el grupo control seguirán el mismo programa de ejercicios terapéuticos que el grupo de intervención, pero sin utilizar la tecnología de Realidad Virtual. Los ejercicios serán idénticos en cuanto a tipo, progresión y carga.

Figura 2: Programa de ejercicios con realidad virtual. 1) Flexión del tronco en posición de pie; 2) Extensión del tronco en posición sentada; 3) Flexión del tronco en posición sentada; 4) Puente de glúteos; 5) Crunch; 6) Inclinación del tronco en posición de pie; 7) Rotación del tronco en posición tumbada y 8) Extensión del tronco en posición tumbada.

Figura 3: Montaje de la RV y entorno virtual.

4.9 Variables a recoger e instrumentos de medida.

1. El Fibromyalgia Impact Questionnaire Revised (FIQR) es un instrumento validado y ampliamente utilizado para evaluar el impacto de la fibromialgia en la vida diaria de los pacientes. Mide tres dimensiones clave: funcionalidad (9 ítems, 0-30 puntos), síntomas (10 ítems, 0-50 puntos) e impacto global (2 ítems, 0-20 puntos), obteniendo una puntuación total de 0 a 100, donde valores más altos reflejan un mayor impacto de la enfermedad. Cada ítem se puntúa de 0 a 10, indicando desde la ausencia hasta el máximo nivel de afectación. La versión validada al español ha demostrado una alta fiabilidad (α de Cronbach = 0.91-0.95) y es considerada el instrumento de referencia para evaluar la efectividad de intervenciones en fibromialgia, debido a su sensibilidad para detectar cambios y su sólido respaldo en la literatura científica^61,62^.
2. El EuroQol-5D-5L (EQ-5D-5L) es un cuestionario validado y traducido al español para evaluar la calidad de vida, desarrollado por el Grupo EuroQol como mejora del EQ-5D-3L. Evalúa cinco dimensiones clave: movilidad, autocuidado, actividades diarias, dolor/malestar y ansiedad/depresión, con cinco niveles de respuesta (1 = mejor estado de salud, 5 = peor). Además, incluye una escala visual analógica (EVA) de 0 a 100 para valorar el estado de salud el día de la evaluación. Su fiabilidad test-retest varía entre 0.69 y 0.94 (ICC)^63^, y es ampliamente utilizado en estudios sobre fibromialgia y dolor crónico por su capacidad para proporcionar una evaluación objetiva y comparativa de la calidad de vida ^44,51,63–66^.
3. Los niveles de ansiedad y depresión de los participantes se evaluarán mediante la Hospital Anxiety and Depression Scale (HADS), un instrumento validado en poblaciones clínicas y no clínicas. Consta de 14 ítems divididos en dos subescalas: ansiedad (HADS-A) y depresión (HADS-D), con puntuaciones de 0 a 21, donde valores más altos indican mayor afectación. La versión en español ha mostrado alta fiabilidad (α de Cronbach = 0.83-0.86 para ansiedad y 0.82-0.84 para depresión; ICC = 0.76-0.93) y una sensibilidad y especificidad cercanas al 80%. En pacientes con fibromialgia, ha demostrado una validez concurrente significativa con medidas de dolor y calidad de vida, lo que la convierte en una herramienta adecuada para esta población, ya que permite evaluar síntomas afectivos sin interferencia de síntomas somáticos propios de la enfermedad. Dado que la fibromialgia se asocia con altas tasas de ansiedad y depresión, su evaluación es clave para comprender el impacto emocional de la enfermedad y valorar la efectividad de las intervenciones terapéuticas.^67,68^.
4. La fatiga de los participantes se evaluará mediante la Multidimensional Fatigue Inventory (MFI-20), un instrumento validado para medir cinco dimensiones de la fatiga: general, física, reducción de la actividad, reducción de la motivación y mental. Consta de 20 ítems, puntuados de 1 (sí, es verdad) a 5 (no, no es verdad), con una puntuación total de 20 a 100, donde valores más altos indican mayor fatiga. Su fiabilidad test-retest varía entre 0.80 y 0.93 (ICC), y la versión en español ha sido validada y ampliamente utilizada en estudios de fibromialgia y enfermedades crónicas por su precisión para evaluar la multidimensionalidad de la fatiga y su impacto en la calidad de vida^69,70^.
5. La calidad del sueño se evaluará mediante la versión española del Pittsburgh Sleep Quality Index (PSQI), un instrumento validado para medir la calidad y los patrones de sueño en el último mes. Consta de 19 ítems agrupados en siete componentes, con una puntuación global de 0 a 21, donde valores más altos indican peor calidad del sueño. Su versión en español ha demostrado alta fiabilidad (α = 0.805; test-retest r = 0.773, p < 0.001) y validez convergente con el FIQR y el SF-36. Su uso está ampliamente respaldado en estudios sobre fibromialgia, siendo una herramienta sensible para detectar alteraciones del sueño en esta población^71,72^.
6. La catastrofización del dolor será evaluada mediante la Pain Catastrophizing Scale (PCS), un cuestionario desarrollado para medir la tendencia a experimentar pensamientos negativos y exagerados en respuesta al dolor. Consta de 13 ítems que evalúan rumiación, magnificación e impotencia, puntuados de 0 (nunca) a 4 (siempre), con una puntuación total de 0 a 52, donde valores más altos indican mayor catastrofización. La versión en español ha demostrado alta fiabilidad (α de Cronbach = 0.79; fiabilidad test-retest ICC = 0.84) y mantiene la estructura trifactorial de la escala original. Además, ha mostrado sensibilidad al cambio con un tamaño del efecto de hasta 2, lo que la hace una herramienta válida y confiable en estudios sobre fibromialgia y dolor crónico, siendo útil para predecir la intensidad del dolor, la discapacidad y la respuesta a intervenciones terapéuticas^73–75^.
7. La sensibilización central de los participantes se evaluará mediante el Central Sensitization Inventory (CSI), un instrumento diseñado para identificar síntomas asociados a la sensibilización central y su impacto en la vida diaria. Consta de 25 ítems que evalúan dolor generalizado, fatiga, problemas de concentración, hipersensibilidad sensorial y alteraciones del sueño, con una puntuación total de 0 a 100, donde valores más altos indican una mayor carga de síntomas. Se considera que puntuaciones superiores a 40 sugieren la presencia de un síndrome de sensibilización central, como la fibromialgia. La versión en español ha demostrado alta fiabilidad test-retest (ICC = 0.82-0.91) y es ampliamente utilizada en estudios sobre dolor crónico, proporcionando una evaluación efectiva del grado de sensibilización central en diversas poblaciones clínicas^76^.
8. La función del sistema nervioso será evaluada mediante el Quantitative Sensory Testing (QST), un conjunto de pruebas estandarizadas que mide la percepción sensorial y la modulación del dolor a través de estímulos mecánicos, térmicos y de presión. Evalúa parámetros clave como umbrales de detección y tolerancia al dolor por presión, detección térmica (calor/frío), hiperalgesia mecánica y térmica, y alodinia, permitiendo diferenciar entre sensibilización periférica y central. Su fiabilidad test-retest varía según el parámetro evaluado (ICC = 0.75-0.95), y ha sido validado en estudios de fibromialgia y dolor crónico, siendo una herramienta fundamental para caracterizar disfunciones en la modulación del dolor y evaluar la eficacia de intervenciones terapéuticas^77,78^.
9. La kinesiofobia de los participantes será evaluada mediante la Tampa Scale for Kinesiophobia (TSK), un cuestionario desarrollado para medir el miedo al movimiento y la evitación de la actividad física en personas con dolor crónico. La versión en español ha sido validada y estructurada en una versión reducida de 11 ítems (TSK-11), que mide dos factores: Actividad Evitativa (AA) y Daño (H). Cada ítem se puntúa en una escala de 1 (totalmente en desacuerdo) a 4 (totalmente de acuerdo), con una puntuación total de 11 a 44, donde valores más altos reflejan mayor kinesiofobia. Su fiabilidad test-retest ha sido moderada a alta (α de Cronbach = 0.79-0.81; ICC = 0.55-0.91), y se ha asociado significativamente con el catastrofismo, la ansiedad y la depresión. La TSK-11 es ampliamente utilizada en estudios sobre fibromialgia y dolor crónico, siendo una herramienta clave para evaluar el impacto del miedo al movimiento en la funcionalidad y la adherencia a tratamientos basados en el ejercicio.^58,79,80^
10. La fuerza y resistencia de los miembros inferiores se evaluará mediante el Sit-to-Stand Test de 30 segundos (STS-30), una prueba funcional que mide la capacidad de levantarse de una silla repetidamente en 30 segundos sin usar los brazos. Es una herramienta ampliamente utilizada para evaluar fuerza muscular, resistencia y funcionalidad en personas con dolor crónico y movilidad reducida. El número total de repeticiones completadas indica el rendimiento funcional, siendo mayores valores indicativos de mejor capacidad. Su fiabilidad test-retest es alta (ICC = 0.84-0.96), y su versión en español ha sido validada en estudios sobre fibromialgia y envejecimiento, siendo clave para evaluar la funcionalidad y la evolución en programas de intervención basados en el ejercicio^81–83^.
11. La movilidad funcional de los participantes se evaluará mediante el Timed Up and Go (TUG) Test, una prueba ampliamente utilizada para medir la velocidad de marcha, equilibrio dinámico y capacidad funcional en personas con fibromialgia. Consiste en cronometrar el tiempo que tarda el participante en levantarse de una silla, caminar 3 metros, girar 180 grados, regresar y sentarse nuevamente. Su fiabilidad test-retest es alta, con un ICC de 0.935 utilizando cronómetro manual y 0.955 con cronómetro automático, recomendándose el uso de dispositivos automáticos para mejorar la precisión y reducir la variabilidad en la medición. Ha sido evaluado y validado previamente en pacientes con fibromialgia y resulta una herramienta clave para evaluar la evolución clínica y la respuesta a intervenciones terapéuticas basadas en el ejercicio^46,84,85^.
12. La fuerza isométrica del cuádriceps de los participantes será evaluada mediante dinamometría, una técnica ampliamente utilizada para cuantificar la función neuromuscular en personas con fibromialgia. Se empleará un dinamómetro portátil, colocado a nivel del tobillo con la rodilla flexionada a 90°, para medir la fuerza máxima de contracción isométrica voluntaria del músculo cuádriceps. En pacientes con fibromialgia, se ha reportado una reducción significativa de la fuerza muscular, lo que puede estar asociado con la fatiga, el dolor crónico y una disminución en la capacidad funcional. La medición de la fuerza del cuádriceps es un indicador clave de la funcionalidad del tren inferior y del riesgo de discapacidad en esta población. La fiabilidad test-retest de la dinamometría isométrica en pacientes con fibromialgia varía entre 0.85 y 0.96 (ICC), y su uso ha sido validado en estudios previos, proporcionando una evaluación objetiva de la respuesta muscular a intervenciones terapéuticas basadas en el ejercicio.
13. La fuerza de agarre de los participantes será evaluada mediante dinamometría manual, un método validado para medir la fuerza isométrica de la mano y un marcador clínico relevante en personas con fibromialgia. Se realizarán tres intentos máximos por extremidad con un dinamómetro de mano, siguiendo protocolos estandarizados con el codo flexionado a 90° y el antebrazo en posición neutra. En pacientes con fibromialgia, la fuerza de agarre suele estar reducida hasta un 40% en comparación con individuos sanos, lo que se asocia con disminución de la funcionalidad, dolor generalizado y fatiga. Además, esta reducción refleja una posible disfunción neuromuscular y sensibilización central, características de esta condición. Su fiabilidad test-retest es alta (ICC = 0.85-0.98) y ha sido validada en estudios previos, siendo una herramienta clave para monitorizar cambios en la funcionalidad y evaluar la respuesta a intervenciones terapéuticas.^82,86^
14. La regulación de la conducta en el ejercicio será evaluada mediante el Behavioral Regulation in Exercise Questionnaire-3 (BREQ-3), un instrumento basado en la Teoría de la Autodeterminación que mide los diferentes tipos de motivación hacia la práctica de ejercicio. Consta de 24 ítems distribuidos en seis dimensiones, permitiendo analizar el grado de autodeterminación en la actividad física. Su versión en español ha sido validada, demostrando adecuada fiabilidad (α de Cronbach = 0.81 para regulación intrínseca y 0.70 para desmotivación) y una estructura factorial sólida, lo que respalda su validez de constructo. En personas con fibromialgia, la motivación es clave para la adherencia a programas de ejercicio, impactando en su funcionalidad y calidad de vida, por lo que el BREQ-3 se presenta como una herramienta fundamental para evaluar estos aspectos en el presente estudio^87^.
15. La experiencia de los participantes con la realidad virtual (RV) será evaluada mediante escalas de intensidad de mareo y satisfacción, ambas medidas en una escala de 0 a 10, para garantizar la tolerancia y aceptación de la intervención. La RV ha demostrado ser una herramienta eficaz en el tratamiento del dolor crónico, incluida la fibromialgia, y su uso en este estudio se monitorizará para asegurar la comodidad de los participantes. Además, se utilizará una NRS-11 para medir el dolor percibido antes y después de cada sesión, proporcionando una evaluación objetiva del impacto del tratamiento.
16. Las creencias de evitación por miedo al dolor serán evaluadas mediante el Fear-Avoidance Beliefs Questionnaire (FABQ), un instrumento ampliamente utilizado en estudios de fibromialgia y dolor crónico para medir el impacto del miedo en la actividad física y el trabajo. Consta de 16 ítems divididos en dos subescalas: actividad física (FABQ-PA) y trabajo (FABQ-W), puntuadas de 0 (totalmente en desacuerdo) a 6 (totalmente de acuerdo). En personas con fibromialgia, puntuaciones más altas en el FABQ se han asociado con mayor discapacidad, dolor y menor adherencia a programas de ejercicio. Su versión en español ha sido validada, mostrando alta fiabilidad test-retest (ICC = 0.84 para FABQ-W y 0.80 para FABQ-PA) y buena consistencia interna (α de Cronbach = 0.88 para FABQ-W y 0.79 para FABQ-PA). Además, el análisis factorial confirmó su estructura bidimensional, consolidando su utilidad como herramienta clave para evaluar el impacto psicológico del dolor en la funcionalidad y la recuperación^88^.
17. La extensión del dolor y la severidad de los síntomas serán evaluadas mediante la escala de dolor generalizado (IDG) y la escala de severidad sintomatica (ESS), herramientas utilizadas en el diagnóstico y seguimiento de la fibromialgia según los criterios del American College of Rheumatology (ACR). La IDG mide la distribución del dolor en 19 regiones corporales (0-19 puntos), mientras que la ESS evalúa la gravedad de síntomas como fatiga, alteraciones del sueño y problemas cognitivos (0-12 puntos). Ambos instrumentos serán aplicados antes y después de la intervención para evaluar el impacto del tratamiento en la extensión del dolor y la severidad de los síntomas.
18. La percepción corporal será evaluada mediante la versión adaptada del Fremantle Body Awareness Questionnaire (FBAQ-B), herramienta que identifica distorsiones en la representación corporal, un fenómeno documentado en personas con fibromialgia. Estas alteraciones incluyen sensaciones de desconexión, cambios en la forma o tamaño del cuerpo y dificultad para localizar partes corporales, vinculadas a la disfunción en la integración somatosensorial y la sensibilización central. La versión española del FBAQ-B ha sido validada, mostrando una estructura unidimensional, alta fiabilidad (α = 0.82) y buena fiabilidad test-retest (ICC = 0.78), además de una validez discriminante y convergente, permitiendo diferenciar entre pacientes y controles. Dado que la realidad virtual (RV) puede modular estos mecanismos al proporcionar un entorno inmersivo que estimula la conciencia corporal, su impacto será analizado antes y después de la intervención. Evaluar estos cambios permitirá determinar si la RV combinada con ejercicio terapéutico mejora la integración sensorial y reduce las alteraciones perceptivas, aportando información novedosa sobre su potencial en el manejo de la fibromialgia^89,90^.

**4.10 Información sobre el análisis de resultados previsto**

Todos los análisis seguirán el principio de intención de tratar. La estadística descriptiva resumirá las características basales. Las diferencias entre grupos en el momento basal se explorarán con pruebas t de Student para muestras independientes o U de Mann–Whitney para variables continuas y pruebas de chi-cuadrado para variables categóricas.

Análisis principal. Los efectos de la intervención se evaluarán mediante una ANCOVA de medidas repetidas de dos vías con un factor intra-sujeto (Tiempo: t0, t1) y un factor inter-sujetos (Grupo: IVR+ejercicio frente a ejercicio). La variable dependiente será la puntuación postintervención (t1) del resultado analizado, ajustada por su valor basal (t0). El sexo y el FIQR basal se incluirán como covariables preespecificadas. La interacción Grupo×Tiempo será el efecto de interés. Se informarán diferencias de medias ajustadas con intervalos de confianza del 95%. El umbral de significación será bilateral p < 0,05.

Los desenlaces secundarios se analizarán con el mismo marco de ANCOVA: la puntuación en t1 como variable dependiente, ajustada por su valor en t0, con Grupo como factor inter-sujetos y las mismas covariables (sexo y FIQR basal). Los resultados se presentarán como diferencias de medias ajustadas con IC del 95%. Los tamaños del efecto dentro de cada grupo (d de Cohen) se calcularán con fines descriptivos.

Todos los análisis se realizarán con SPSS v27.0 (IBM Corp., Armonk, NY, EE. UU.).

**5. Tratamiento de los datos**: Todo el tratamiento de los datos debe cumplir con el RGPD y la LOPD-DGG.

- 1. Recogida y almacenamiento de datos.

Los datos se recogerán de forma presencial por evaluadores formados en t0 y t1. Los formularios en papel se introducirán por duplicado en una base de datos electrónica protegida por contraseña. La información de identificación personal (PII) se registrará únicamente en los formularios de consentimiento y en un registro de vinculación separado, mantenido fuera de línea y guardado en un armario cerrado con llave. Los conjuntos de datos desidentificados para el análisis se conservarán y se compartirán según el plan de disponibilidad de datos (DOI de Zenodo), mientras que el registro de vinculación se destruirá en el plazo de un mes tras el cierre de la base de datos.

- 1. Análisis, codificación y destrucción.

Una vez analizados los datos en SPSS exportados desde un Excel, se almacenarán en el ordenador y bajo contraseña durante un tiempo limitado hasta su destrucción (por ordenador se eliminarán todos los archivos y el Excel principal y en papel se destruirán en la trituradora de papel).

- 1. Identificación del responsable de los datos.

Se recogerán datos de identificación personal en los formularios de consentimiento y en un registro de vinculación separado, mantenido fuera de línea; el conjunto de datos para el análisis estará completamente desidentificado.

- 1. Tiempos de cada tratamiento.

Los datos serán registrados en 2 momentos (t) del estudio: t0) antes de la intervención, t1) inmediatamente después de la intervención. Todos los datos serán almacenados y tratados durante el tiempo estrictamente necesario para el desarrollo del estudio, siguiendo las siguientes fases:

**Recogida y procesamiento de datos:** Se realizará durante el período de intervención del estudio, garantizando que solo el personal autorizado tenga acceso a la información.

**Almacenamiento y análisis:** Los datos serán conservados durante el tiempo requerido para su análisis y posterior publicación de los resultados, asegurando su custodia bajo medidas de seguridad adecuadas.

**Finalización y eliminación:** Una vez concluido el estudio y cumplidos los plazos legales de conservación, los datos serán anonimizados o eliminados de forma segura, conforme a la normativa vigente.

En todo momento, los participantes podrán ejercer sus derechos de acceso, rectificación, limitación o supresión de sus datos, tal como establece la legislación en materia de protección de datos.

5.5 Personas participantes en cada una de estas fases

Los participantes del estudio serán adultos con diagnóstico de fibromialgia, seleccionados según los criterios de inclusión y exclusión establecidos. Antes de su participación, recibirán una hoja informativa detallada y firmarán el consentimiento informado tras resolver cualquier duda.

Se garantizará un seguimiento continuo para monitorizar posibles efectos adversos y asegurar el bienestar de los participantes. Además, podrán retirarse del estudio en cualquier momento sin repercusiones.

Este procedimiento asegura el cumplimiento de los estándares éticos y metodológicos, garantizando la validez y seguridad del estudio.

- 1. Lugares en los que estarán almacenados los datos

Los datos online estarán almacenados en archivos Excel encriptados por el investigador principal en uno de los ordenadores del grupo de investigación que presentará una contraseña. Los datos en papel se guardarán en un casillero específico para este estudio el cual estará bajo llave.

**6. Consideraciones éticas**

Este estudio seguirá las normas CONSORT para Ensayos Controlados Aleatorizados y será evaluado por el Comité de Ética de la Universidad CEU Cardenal Herrera, garantizando el cumplimiento de todas las directrices éticas necesarias. Además, se ajustará a los principios de la Declaración de Helsinki (2013) y a las Normas de Buenas Prácticas Clínicas (BPC) para asegurar el respeto, la seguridad y el bienestar de los participantes.

La fibromialgia es una patología crónica que tiene un impacto significativo en la calidad de vida de quienes la padecen. Por ello, cualquier intervención terapéutica debe ser cuidadosamente diseñada para maximizar sus beneficios sin generar riesgos innecesarios. En este caso, tanto la realidad virtual inmersiva (RVI) como el ejercicio terapéutico han sido ampliamente estudiados y han demostrado ser seguros en poblaciones similares. No obstante, se tendrán en cuenta posibles efectos transitorios, como la fatiga o el mareo leve asociado a la RVI, y se llevará a cabo un seguimiento cercano para garantizar el bienestar de los participantes.

Todos los sujetos que participen en el estudio recibirán información detallada sobre los objetivos, la metodología, los posibles beneficios y los riesgos de la intervención. Se obtendrá su consentimiento informado antes de la participación, asegurando que tomen una decisión libre y consciente. Además, podrán retirarse del estudio en cualquier momento sin que esto tenga consecuencias negativas para ellos.

El tratamiento de los datos se realizará conforme a la Ley Orgánica de Protección de Datos y Garantía de Derechos Digitales (LOPD-GDD) y al Reglamento General de Protección de Datos (RGPD 2016/679), garantizando la confidencialidad y el anonimato de la información. Los datos serán almacenados en servidores seguros de la Universidad CEU Cardenal Herrera, con acceso restringido solo a los investigadores responsables. Una vez finalizado el estudio, la información será eliminada siguiendo los protocolos de seguridad establecidos.

Este proyecto no solo busca aportar evidencia científica sobre la eficacia de la realidad virtual en pacientes con fibromialgia, sino que también se alinea con el enfoque integral de la salud que promueve la Organización Mundial de la Salud (OMS). Se espera que los resultados ayuden a diseñar estrategias terapéuticas más efectivas y adaptadas a las necesidades reales de los pacientes.

En definitiva, el estudio se llevará a cabo con un compromiso absoluto con la ética, asegurando que la investigación contribuya al conocimiento científico sin comprometer en ningún momento el bienestar de los participantes.

**Justificación Científica:**

La fibromialgia es una patología crónica caracterizada por dolor generalizado, fatiga y alteraciones en la percepción corporal, lo que limita significativamente la funcionalidad y calidad de vida de quienes la padecen. En la actualidad, la evidencia científica respalda el uso del ejercicio terapéutico como una estrategia eficaz para mejorar los síntomas de la enfermedad. Sin embargo, la adherencia a estos programas suele verse afectada por la percepción del dolor, el miedo al movimiento (kinesiofobia) y la alteración en la representación corporal de los pacientes.

El presente estudio busca abordar estas limitaciones mediante el uso de realidad virtual inmersiva (RV) combinada con un programa de ejercicio terapéutico. La RV permite modificar la percepción visual y propioceptiva del movimiento, lo que podría contribuir a una mayor tolerancia al ejercicio, reduciendo la percepción de amenaza y el impacto del dolor. Además, se espera que la intervención ayude a modular la representación del esquema corporal, favoreciendo una mayor integración sensoriomotora y mejorando la funcionalidad.

A pesar del creciente interés en el uso de realidad virtual en el manejo del dolor crónico, la evidencia sobre su efectividad en pacientes con fibromialgia sigue siendo limitada. Este estudio pretende aportar datos sólidos sobre su aplicabilidad en esta población, analizando no solo los efectos en la reducción del dolor y la mejora funcional, sino también en la modulación de la percepción corporal y el bienestar general de los pacientes.

**Justificación Ética:**

El presente estudio cumple con los principios establecidos en la Declaración de Helsinki (2013) y sigue las Normas de Buenas Prácticas Clínicas, garantizando el respeto y protección de los participantes en todas las fases de la investigación.

La intervención propuesta no supone riesgos significativos para los participantes, ya que tanto la realidad virtual como el ejercicio terapéutico han demostrado ser seguros en población con fibromialgia. No obstante, podrían experimentarse efectos transitorios como mareo leve o incomodidad durante la exposición a la realidad virtual, los cuales serán monitorizados para asegurar el bienestar de los participantes.

Todos los procedimientos de recolección y tratamiento de datos se llevarán a cabo de acuerdo con el Reglamento General de Protección de Datos (RGPD) y la Ley Orgánica de Protección de Datos y Garantía de los Derechos Digitales (LOPD-GDD), asegurando la confidencialidad y anonimización de la información.

Los participantes recibirán información detallada sobre el estudio y se obtendrá su consentimiento informado antes de la participación. Además, se les garantizará el derecho a retirarse del estudio en cualquier momento sin que esto implique perjuicio alguno.

Este enfoque garantiza que la investigación no solo contribuirá al conocimiento sobre la efectividad de la realidad virtual en fibromialgia, sino que también respetará y protegerá los derechos, bienestar y autonomía de los participantes.

**7. Bibliografía**

1. Clauw DJ. Fibromyalgia. JAMA. 2014 Apr 16;311(15):1547.

2. Sandikci SC, Ozbalkan Z. Fatigue in rheumatic diseases. Eur J Rheumatol. 2015 Aug 27;2(3):109–13.

3. Bennett RM, Jones J, Turk DC, Russell IJ, Matallana L. An internet survey of 2,596 people with fibromyalgia. BMC Musculoskelet Disord. 2007 Dec 9;8(1):27.

4. Kleinman L, Mannix S, Arnold LM, Burbridge C, Howard K, McQuarrie K, et al. Assessment of sleep in patients with fibromyalgia: qualitative development of the fibromyalgia sleep diary. Health Qual Life Outcomes. 2014 Dec 14;12(1):111.

5. WALITT B, FITZCHARLES MA, HASSETT AL, KATZ RS, HÄUSER W, WOLFE F. The Longitudinal Outcome of Fibromyalgia: A Study of 1555 Patients. J Rheumatol. 2011 Oct;38(10):2238–46.

6. Ifergane G, Buskila D, Simiseshvely N, Zeev K, Cohen H. Prevalence of Fibromyalgia Syndrome in Migraine Patients. Cephalalgia. 2006 Apr 1;26(4):451–6.

7. Mathieu N. Comorbidités somatiques dans le Syndrome de l’Intestin Irritable : fibromyalgie, syndrome de fatigue chronique et cystite interstitielle/syndrome de la vessie douloureuse. Gastroenterol Clin Biol. 2009 Feb;33:S17–25.

8. Nickel JC, Tripp DA, Pontari M, Moldwin R, Mayer R, Carr LK, et al. Interstitial Cystitis/Painful Bladder Syndrome and Associated Medical Conditions With an Emphasis on Irritable Bowel Syndrome, Fibromyalgia and Chronic Fatigue Syndrome. Journal of Urology. 2010 Oct;184(4):1358–63.

9. Kalichman L. Association between fibromyalgia and sexual dysfunction in women. Clin Rheumatol. 2009 Apr 23;28(4):365–9.

10. Solano C, Martinez A, Becerril L, Vargas A, Figueroa J, Navarro C, et al. Autonomic Dysfunction in Fibromyalgia Assessed by the Composite Autonomic Symptoms Scale (COMPASS). JCR: Journal of Clinical Rheumatology. 2009 Jun;15(4):172–6.

11. Vincent A, McAllister SJ, Singer W, Toussaint LL, Sletten DM, Whipple MO, et al. A Report of the Autonomic Symptom Profile in Patients With Fibromyalgia. JCR Journal of Clinical Rheumatology. 2014 Mar;20(2):106–8.

12. Wolfe F, Smythe HA, Yunus MB, Bennett RM, Bombardier C, Goldenberg DL, et al. The american college of rheumatology 1990 criteria for the classification of fibromyalgia. Arthritis Rheum. 1990 Feb 9;33(2):160–72.

13. Yunus MB, Aldag JC. Restless legs syndrome and leg cramps in fibromyalgia syndrome: a controlled study. BMJ. 1996 May 25;312(7042):1339–1339.

14. Viola-Saltzman M, Watson NF, Bogart A, Goldberg ; Jack, Buchwald D, Watson SM;, et al. High Prevalence of Restless Legs Syndrome among Patients with Fibromyalgia: A Controlled Cross-Sectional Study. Vol. 6, Journal of Clinical Sleep Medicine. 2010.

15. Stehlik R, Arvidsson L, Ulfberg J. Restless Legs Syndrome Is Common among Female Patients with Fibromyalgia. Eur Neurol. 2009;61(2):107–11.

16. Jones KD, Horak FB, Winters-Stone K, Irvine JM, Bennett RM. Fibromyalgia Is Associated With Impaired Balance and Falls. JCR: Journal of Clinical Rheumatology. 2009 Jan;15(1):16–21.

17. Kessler RC, Berglund P, Demler O, Jin R, Koretz D, Merikangas KR, et al. The Epidemiology of Major Depressive Disorder. JAMA. 2003 Jun 18;289(23):3095.

18. González E, Elorza J, Failde I. Fibromyalgia and psychiatric comorbidity: their effect on the quality of life patients. Actas Esp Psiquiatr. 2010;38(5):295–300.

19. Galvez-Sánchez CM, Duschek S, Reyes del Paso GA. <p>Psychological impact of fibromyalgia: current perspectives</p>. Psychol Res Behav Manag. 2019 Feb;Volume 12:117–27.

20. Branco JC, Bannwarth B, Failde I, Abello Carbonell J, Blotman F, Spaeth M, et al. Prevalence of Fibromyalgia: A Survey in Five European Countries. Semin Arthritis Rheum. 2010 Jun;39(6):448–53.

21. White KP, Speechley M, Harth M, Ostbye T. The London Fibromyalgia Epidemiology Study: the prevalence of fibromyalgia syndrome in London, Ontario. J Rheumatol. 1999 Jul;26(7):1570–6.

22. Spaeth M. Epidemiology, costs, and the economic burden of fibromyalgia. Arthritis Res Ther. 2009;11(3):117.

23. Häuser W, Sarzi-Puttini P, Fitzcharles MA. Fibromyalgia syndrome: under-, over- and misdiagnosis. Clin Exp Rheumatol. 2019;37 Suppl 116(1):90–7.

24. Lachaine J, Beauchemin C, Landry PA. Clinical and Economic Characteristics of Patients With Fibromyalgia Syndrome. Clin J Pain. 2010 May;26(4):284–90.

25. Berger A, Dukes E, Martin S, Edelsberg J, Oster G. Characteristics and healthcare costs of patients with fibromyalgia syndrome. Int J Clin Pract. 2007 Jul 26;61(9):1498–508.

26. Knight T, Schaefer C, Chandran, Zlateva G, Winkelmann, Perrot. Health-resource use and costs associated with fibromyalgia in France, Germany, and the United States. ClinicoEconomics and Outcomes Research. 2013 Apr;171.

27. Lacasse A, Bourgault P, Choinière M. Fibromyalgia-related costs and loss of productivity: a substantial societal burden. BMC Musculoskelet Disord. 2016 Dec 16;17(1):168.

28. Guymer EK, Littlejohn GO, Brand CK, Kwiatek RA. Fibromyalgia onset has a high impact on work ability in Australians. Intern Med J. 2016 Sep 16;46(9):1069–74.

29. Taylor SJ, Steer M, Ashe SC, Furness PJ, Haywood-Small S, Lawson K. Patients’ perspective of the effectiveness and acceptability of pharmacological and non-pharmacological treatments of fibromyalgia. Scand J Pain. 2019 Jan 28;19(1):167–81.

30. Taylor SJ, Steer M, Ashe SC, Furness PJ, Haywood-Small S, Lawson K. Patients’ perspective of the effectiveness and acceptability of pharmacological and non-pharmacological treatments of fibromyalgia. Scand J Pain. 2019 Jan 1;19(1):167–81.

31. Kayo AH, Peccin MS, Sanches CM, Trevisani VFM. Effectiveness of physical activity in reducing pain in patients with fibromyalgia: a blinded randomized clinical trial. Rheumatol Int. 2012 Aug 19;32(8):2285–92.

32. Bircan Ç, Karasel SA, Akgün B, El Ö, Alper S. Effects of muscle strengthening versus aerobic exercise program in fibromyalgia. Rheumatol Int. 2008 Apr 3;28(6):527–32.

33. Assumpção A, Matsutani LA, Yuan SL, Santo AS, Sauer J, Mango P, et al. Muscle stretching exercises and resistance training in fibromyalgia: which is better? A three-arm randomized controlled trial. Eur J Phys Rehabil Med. 2018 Sep;54(5).

34. Larsson A, Palstam A, Löfgren M, Ernberg M, Bjersing J, Bileviciute-Ljungar I, et al. Resistance exercise improves muscle strength, health status and pain intensity in fibromyalgia—a randomized controlled trial. Arthritis Res Ther. 2015 Jun 18;17(1):161.

35. Gavi MBRO, Vassalo DV, Amaral FT, Macedo DCF, Gava PL, Dantas EM, et al. Strengthening Exercises Improve Symptoms and Quality of Life but Do Not Change Autonomic Modulation in Fibromyalgia: A Randomized Clinical Trial. PLoS One. 2014 Mar 20;9(3):e90767.

36. HÃ¤kkinen A, HÃ¤kkinen K, Hannonen P, Alen M. Strength training induced adaptations in neuromuscular function of premenopausal women with fibromyalgia: comparison with healthy women. Ann Rheum Dis. 2001 Jan;60(1):21–6.

37. Sañudo B, Galiano D, Carrasco L, Blagojevic M, de Hoyo M, Saxton J. Aerobic Exercise Versus Combined Exercise Therapy in Women With Fibromyalgia Syndrome: A Randomized Controlled Trial. Arch Phys Med Rehabil. 2010 Dec;91(12):1838–43.

38. Gowans SE, deHueck A, Voss S, Silaj A, Abbey SE, Reynolds WJ. Effect of a randomized, controlled trial of exercise on mood and physical function in individuals with fibromyalgia. Arthritis Rheum. 2001 Dec;45(6):519–29.

39. Wang C, Schmid CH, Fielding RA, Harvey WF, Reid KF, Price LL, et al. Effect of tai chi versus aerobic exercise for fibromyalgia: comparative effectiveness randomized controlled trial. BMJ. 2018 Mar 21;k851.

40. Gupta A, Scott K, Dukewich M. Innovative Technology Using Virtual Reality in the Treatment of Pain: Does It Reduce Pain via Distraction, or Is There More to It? Pain Medicine. 2018 Jan 1;19(1):151–9.

41. Li L, Yu F, Shi D, Shi J, Tian Z, Yang J, et al. Application of virtual reality technology in clinical medicine [Internet]. Vol. 9, Am J Transl Res. 2017. Available from: www.ajtr.org/ISSN:1943-8141/AJTR0055713

42. Lee SH, Jung H, Yun SJ, Oh B, Seo HG. Upper Extremity Rehabilitation Using Fully Immersive Virtual Reality Games With a Head Mount Display: A Feasibility Study. PM&R. 2020 Mar 3;12(3):257–62.

43. Carvalho MS de, Carvalho LC, Menezes F da S, Frazin A, Gomes E da C, Iunes DH. Effects of Exergames in Women with Fibromyalgia: A Randomized Controlled Study. Games Health J. 2020 Oct 1;9(5):358–67.

44. Collado-Mateo D, Dominguez-Muñoz FJ, Adsuar JC, Garcia-Gordillo MA, Gusi N. Effects of Exergames on Quality of Life, Pain, and Disease Effect in Women With Fibromyalgia: A Randomized Controlled Trial. Arch Phys Med Rehabil. 2017 Sep;98(9):1725–31.

45. Villafaina S, Collado-Mateo D, Fuentes JP, Rohlfs-Domínguez P, Gusi N. Effects of Exergames on Brain Dynamics in Women with Fibromyalgia: A Randomized Controlled Trial. J Clin Med. 2019 Jul 11;8(7):1015.

46. Collado-Mateo D, Dominguez-Muñoz FJ, Adsuar JC, Merellano-Navarro E, Gusi N. Exergames for women with fibromyalgia: a randomised controlled trial to evaluate the effects on mobility skills, balance and fear of falling. PeerJ. 2017 Apr 20;5:e3211.

47. Garcia-Palacios A, Herrero R, Vizcaíno Y, Belmonte MA, Castilla D, Molinari G, et al. Integrating Virtual Reality With Activity Management for the Treatment of Fibromyalgia. Clin J Pain. 2015 Jun;31(6):564–72.

48. Leon-Llamas JL, Villafaina S, Murillo-Garcia A, Dominguez-Muñoz FJ, Gusi N. Effects of 24-Week Exergame Intervention on the Gray Matter Volume of Different Brain Structures in Women with Fibromyalgia: A Single-Blind, Randomized Controlled Trial. J Clin Med. 2020 Jul 30;9(8):2436.

49. Martín‐Martínez JP, Villafaina S, Collado‐Mateo D, Pérez‐Gómez J, Gusi N. Effects of 24‐week exergame intervention on physical function under single‐ and dual‐task conditions in fibromyalgia: A randomized controlled trial. Scand J Med Sci Sports. 2019 Oct 3;29(10):1610–7.

50. Polat M, Kahveci A, Muci B, Günendi Z, Kaymak Karataş G. The Effect of Virtual Reality Exercises on Pain, Functionality, Cardiopulmonary Capacity, and Quality of Life in Fibromyalgia Syndrome: A Randomized Controlled Study. Games Health J. 2021 Jun 1;10(3):165–73.

51. Villafaina S, Collado-Mateo D, Domínguez-Muñoz FJ, Fuentes-García JP, Gusi N. Benefits of 24-Week Exergame Intervention on Health-Related Quality of Life and Pain in Women with Fibromyalgia: A Single-Blind, Randomized Controlled Trial. Games Health J. 2019 Dec 1;8(6):380–6.

52. Villafaina S, Borrega-Mouquinho Y, Fuentes-García JP, Collado-Mateo D, Gusi N. Effect of Exergame Training and Detraining on Lower-Body Strength, Agility, and Cardiorespiratory Fitness in Women with Fibromyalgia: Single-Blinded Randomized Controlled Trial. Int J Environ Res Public Health. 2019 Dec 24;17(1):161.

53. Cortés-Pérez I, Zagalaz-Anula N, Ibancos-Losada M del R, Nieto-Escámez FA, Obrero-Gaitán E, Osuna-Pérez MC. Virtual Reality-Based Therapy Reduces the Disabling Impact of Fibromyalgia Syndrome in Women: Systematic Review with Meta-Analysis of Randomized Controlled Trials. J Pers Med. 2021 Nov 9;11(11):1167.

54. Rose T, Nam CS, Chen KB. Immersion of virtual reality for rehabilitation - Review. Appl Ergon. 2018 May;69:153–61.

55. Kozhevnikov M, Kosslyn S, Shephard J. Spatial versus object visualizers: A new characterization of visual cognitive style. Mem Cognit. 2005 Jun;33(4):710–26.

56. Shahrbanian S, Ma X, Aghaei N, Korner-Bitensky N, Moshiri K, Simmonds MJ. Use of virtual reality (immersive vs. non immersive) for pain management in children and adults: A systematic review of evidence from randomized controlled trials [Internet]. Vol. 2012, Pelagia Research Library European Journal of Experimental Biology. Available from: www.pelagiaresearchlibrary.com

57. Darnall BD, Krishnamurthy P, Tsuei J, Minor JD. Self-Administered Skills-Based Virtual Reality Intervention for Chronic Pain: Randomized Controlled Pilot Study. JMIR Form Res. 2020 Jul 7;4(7):e17293.

58. Gulsen C, Soke F, Eldemir K, Apaydin Y, Ozkul C, Guclu-Gunduz A, et al. Effect of fully immersive virtual reality treatment combined with exercise in fibromyalgia patients: a randomized controlled trial. Assistive Technology. 2022 May 4;34(3):256–63.

59. Venuturupalli RS, Chu T, Vicari M, Kumar A, Fortune N, Spielberg B. Virtual Reality–Based Biofeedback and Guided Meditation in Rheumatology: A Pilot Study. ACR Open Rheumatol. 2019 Dec 11;1(10):667–75.

60. Gulsen C, Soke F, Eldemir K, Apaydin Y, Ozkul C, Guclu-Gunduz A, et al. Effect of fully immersive virtual reality treatment combined with exercise in fibromyalgia patients: a randomized controlled trial. Assistive Technology. 2020;1–8.

61. Monterde S, Salvat I, Montull S, Fernández-Ballart J. Validación de la versión española del Fibromyalgia Impact Questionnaire. Revista Española de Reumatología [Internet]. 2004 Nov 1 [cited 2025 Jan 29];31(9):507–13. Available from: http://www.elsevier.es/es-revista-revista-espanola-reumatologia-29-articulo-validacion-version-espanola-del-fibromyalgia-13068512

62. Salgueiro M, García-Leiva JM, Ballesteros J, Hidalgo J, Molina R, Calandre EP. Validation of a Spanish version of the Revised Fibromyalgia Impact Questionnaire (FIQR). Health Qual Life Outcomes. 2013 Dec 1;11(1):132.

63. van Hout B, Janssen MF, Feng YS, Kohlmann T, Busschbach J, Golicki D, et al. Interim Scoring for the EQ-5D-5L: Mapping the EQ-5D-5L to EQ-5D-3L Value Sets. Value in Health. 2012 Jul;15(5):708–15.

64. Herdman M, Gudex C, Lloyd A, Janssen MF, Kind P, Parkin D, et al. Development and preliminary testing of the new five-level version of EQ-5D (EQ-5D-5L). Quality of Life Research. 2011 Dec 9;20(10):1727–36.

65. Hernandez G, Garin O, Pardo Y, Vilagut G, Pont À, Suárez M, et al. Validity of the EQ–5D–5L and reference norms for the Spanish population. Quality of Life Research. 2018 Sep 16;27(9):2337–48.

66. Mendoza-Muñoz M, Morenas-Martín J, Rodal M, García-Matador J, García-Gordillo MÁ, Calzada-Rodríguez JI. Knowledge about Fibromyalgia in Fibromyalgia Patients and Its Relation to HRQoL and Physical Activity. Biology (Basel). 2021 Jul 16;10(7):673.

67. Vallejo MA, Rivera J, Esteve-Vives J, Rodríguez-Muñoz MF. Uso del cuestionario Hospital Anxiety and Depression Scale (HADS) para evaluar la ansiedad y la depresión en pacientes con fibromialgia. Rev Psiquiatr Salud Ment. 2012 Apr;5(2):107–14.

68. Herrero MJ, Blanch J, Peri JM, De Pablo J, Pintor L, Bulbena A. A validation study of the hospital anxiety and depression scale (HADS) in a Spanish population. Gen Hosp Psychiatry. 2003 Jul;25(4):277–83.

69. Munguía-Izquierdo D, Segura-Jiménez V, Camiletti-Moirón D, Pulido-Martos M, Alvarez-Gallardo IC, Romero A, et al. Multidimensional Fatigue Inventory: Spanish adaptation and psychometric properties for fibromyalgia patients. The Al-Andalus study. Clin Exp Rheumatol. 2012;30(6 Suppl 74):94–102.

70. Williams DA, Arnold LM. Measures of fibromyalgia: Fibromyalgia Impact Questionnaire (FIQ), Brief Pain Inventory (BPI), Multidimensional Fatigue Inventory (MFI‐20), Medical Outcomes Study (MOS) Sleep Scale, and Multiple Ability Self‐Report Questionnaire (MASQ). Arthritis Care Res (Hoboken). 2011 Nov 7;63(S11).

71. Hita-Contreras F, Martínez-López E, Latorre-Román PA, Garrido F, Santos MA, Martínez-Amat A. Reliability and validity of the Spanish version of the Pittsburgh Sleep Quality Index (PSQI) in patients with fibromyalgia. Rheumatol Int. 2014 Jul 8;34(7):929–36.

72. Osorio CD, Gallinaro AL, Lorenzi-Filho G, Lage L V. Sleep quality in patients with fibromyalgia using the Pittsburgh Sleep Quality Index. J Rheumatol. 2006 Sep;33(9):1863–5.

73. García Campayo J, Rodero B, Alda M, Sobradiel N, Montero J, Moreno S. Validación de la versión española de la escala de la catastrofización ante el dolor (Pain Catastrophizing Scale) en la fibromialgia. Med Clin (Barc). 2008 Oct;131(13):487–92.

74. Morris LD, Grimmer-Somers KA, Spottiswoode B, Louw QA. Virtual reality exposure therapy as treatment for pain catastrophizing in fibromyalgia patients: proof-of-concept study (Study Protocol). BMC Musculoskelet Disord. 2011 Dec 30;12(1):85.

75. Sullivan MJ. The Pain Catastrophizing Scale User Manual. 1995.

76. Cuesta-Vargas AI, Roldan-Jimenez C, Neblett R, Gatchel RJ. Cross-cultural adaptation and validity of the Spanish central sensitization inventory. Springerplus. 2016 Dec 21;5(1):1837.

77. Weaver KR, Griffioen MA, Klinedinst NJ, Galik E, Duarte AC, Colloca L, et al. Quantitative Sensory Testing Across Chronic Pain Conditions and Use in Special Populations. Frontiers in pain research (Lausanne, Switzerland). 2021;2:779068.

78. Wodehouse T, Poply K, Ramaswamy S, Snidvongs S, Bourke J, Tahir H, et al. A pilot study investigating whether quantitative sensory testing alters after treatment in patients with fibromyalgia. Br J Pain. 2018 Nov 15;12(4):250–6.

79. Gómez-Pérez L, López-Martínez AE, Ruiz-Párraga GT. Psychometric Properties of the Spanish Version of the Tampa Scale for Kinesiophobia (TSK). J Pain. 2011 Apr;12(4):425–35.

80. Roelofs J, Goubert L, Peters ML, Vlaeyen JWS, Crombez G. The Tampa Scale for Kinesiophobia: further examination of psychometric properties in patients with chronic low back pain and fibromyalgia. European Journal of Pain. 2004 Oct 11;8(5):495–502.

81. Martín-Martínez JP, Collado-Mateo D, Domínguez-Muñoz FJ, Villafaina S, Gusi N, Pérez-Gómez J. Reliability of the 30 s Chair Stand Test in Women with Fibromyalgia. Int J Environ Res Public Health. 2019 Jul 2;16(13):2344.

82. Carbonell-Baeza A, Álvarez-Gallardo I, Segura-Jiménez V, Castro-Piñero J, Ruiz J, Delgado-Fernández M, et al. Reliability and Feasibility of Physical Fitness Tests in Female Fibromyalgia Patients. Int J Sports Med. 2014 Oct 20;36(02):157–62.

83. Rikli RE, Jones CJ. Development and Validation of a Functional Fitness Test for Community-Residing Older Adults. J Aging Phys Act. 1999 Apr;7(2):129–61.

84. Podsiadlo D, Richardson S. The Timed “Up &amp; Go”: A Test of Basic Functional Mobility for Frail Elderly Persons. J Am Geriatr Soc. 1991 Feb 27;39(2):142–8.

85. Collado-Mateo D, Domínguez-Muñoz FJ, Adsuar JC, Merellano-Navarro E, Olivares PR, Gusi N. Reliability of the Timed Up and Go Test in Fibromyalgia. Rehabilitation Nursing. 2018 Jan;43(1):35–9.

86. Nordenskiöld UM, Grimby G. Grip Force in Patients with Rheumatoid Arthritis and Fibromyalgia and in Healthy Subjects. A Study with the Grippit Instrument. Scand J Rheumatol. 1993 Jan 12;22(1):14–9.

87. 3810.

88. Kovacs FM, Muriel A, Medina JM, Abraira V, Sánchez MDC, Jaúregui JO. Psychometric Characteristics of the Spanish Version of the FAB Questionnaire. Spine (Phila Pa 1976). 2006 Jan;31(1):104–10.

89. García-Dopico N, De La Torre-Luque A, Wand BM, Velasco-Roldán O, Sitges C. The cross-cultural adaptation, validity, and reliability of the Spanish version of the Fremantle Back Awareness Questionnaire. Front Psychol. 2023;14.

90. Świdrak J, Rodriguez T, Polino L, Arias A, Torres X, Sanchez-Vives M V. Drawing the lines of fibromyalgia: a mixed-methods approach to mapping body image, body schema, and emotions in patient subtypes. Psychol Health Med. 2024 Nov 18;1–21.

**7. ANEXOS**

**ANEXO I: Fibromyalgia Impact Questionnaire Revised (FIQR).**

**ANEXO II: EQ-5D-5L**

**ANEXO III: Hospital Anxiety and Depression Scale (HADS).**

**ANEXO IV:** **Multidimensional Fatigue Inventory (MFI-20)**.

**ANEXO V: Pittsburgh Sleep Quality Index (PSQI).**

**ANEXO VI: Pain Catastrophizing Scale (PCS).**

**ANEXO VII: Central Sensitization Inventory (CSI).**

**ANEXO VIII: Tampa Scale for Kinesiophobia (TSK).**

**ANEXO IX: Behavioral Regulation in Exercise Questionnaire-3 (BREQ-3).**

**ANEXO X: Fear-Avoidance Beliefs Questionnaire (FABQ).**

**ANEXO XI: Fremantle Back Awareness Questionnaire (FBAQ).**

**ANEXO I**

**
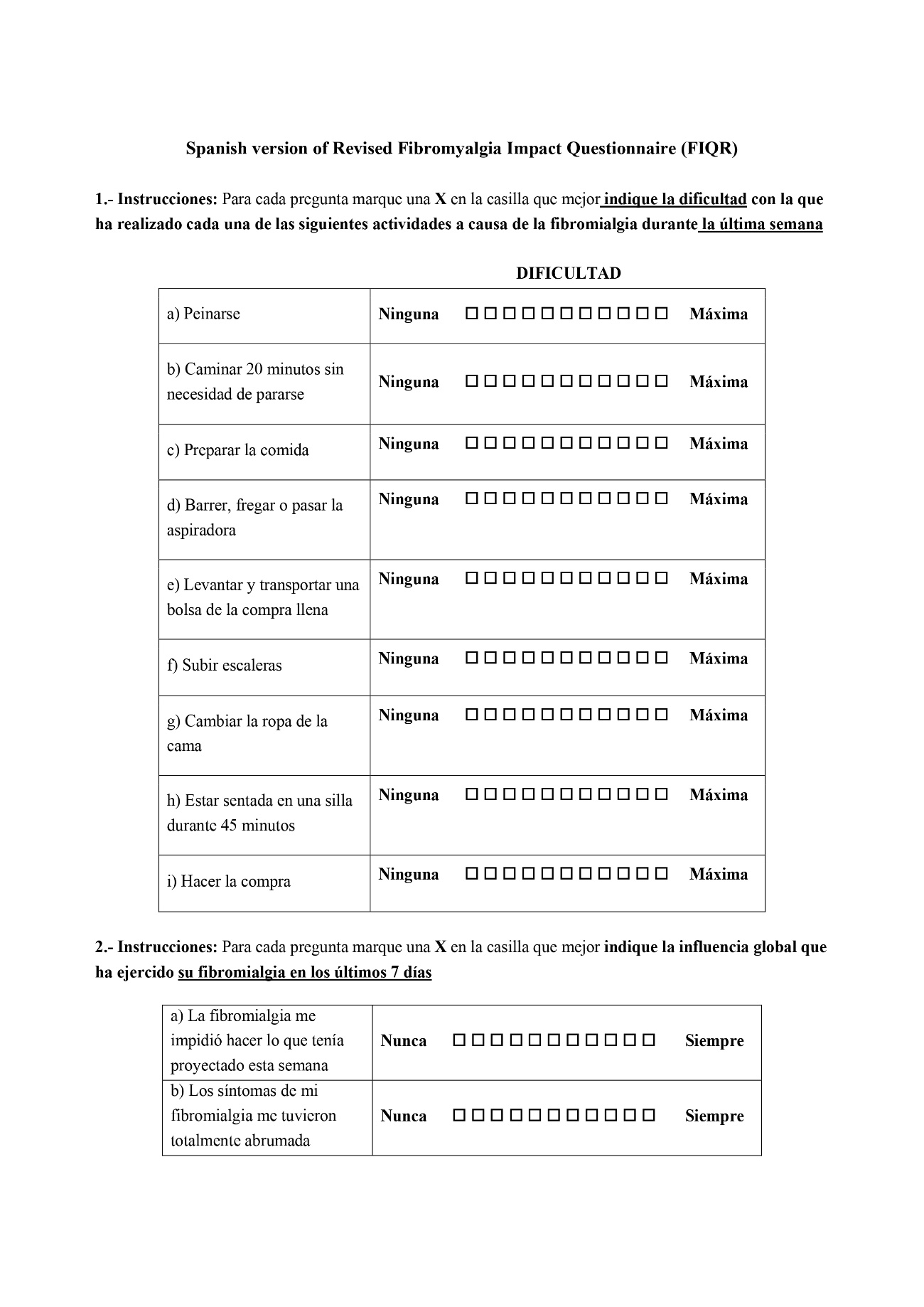
**

**
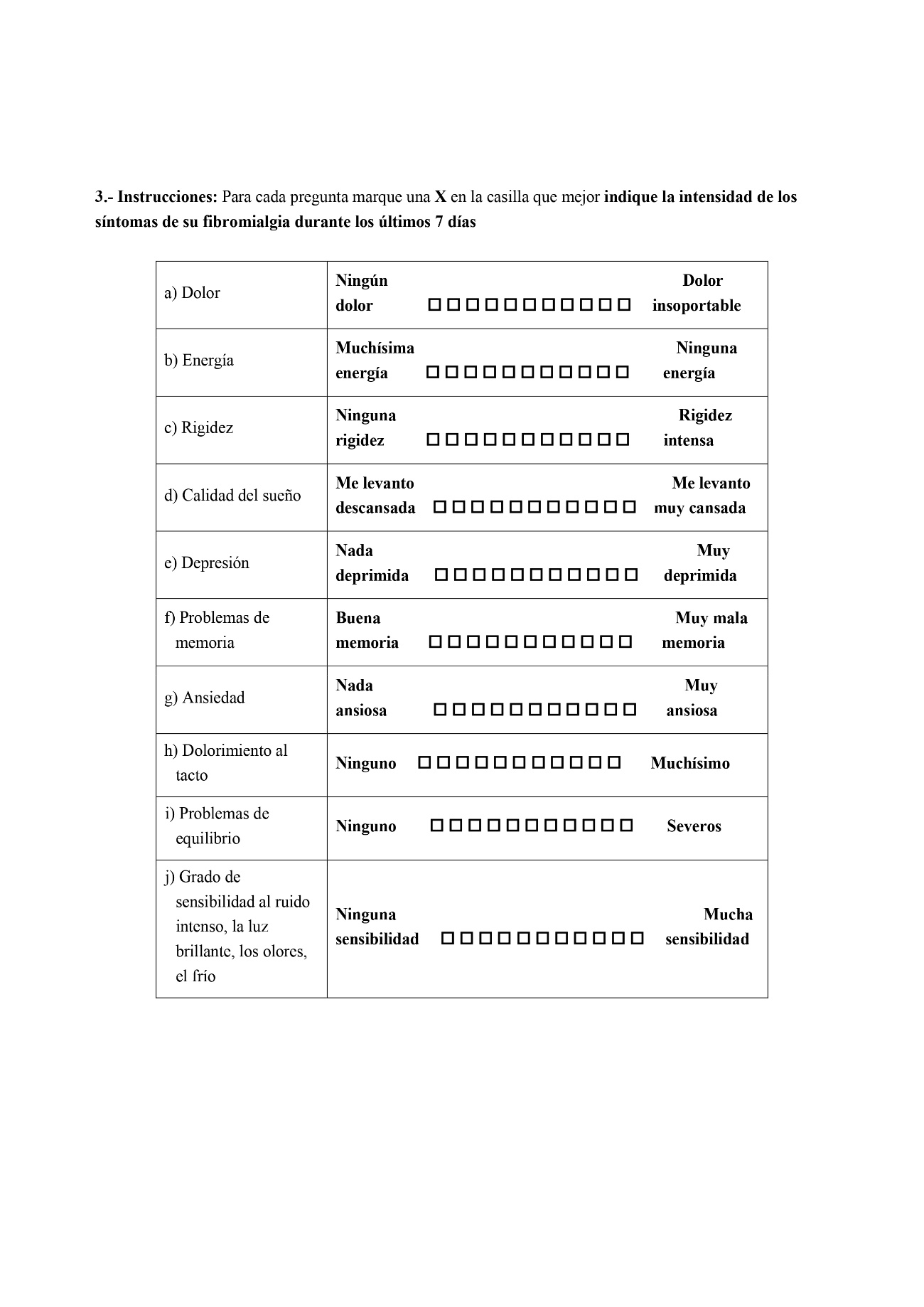
**

**ANEXO II**

**(EQ-5D-5L)**

**
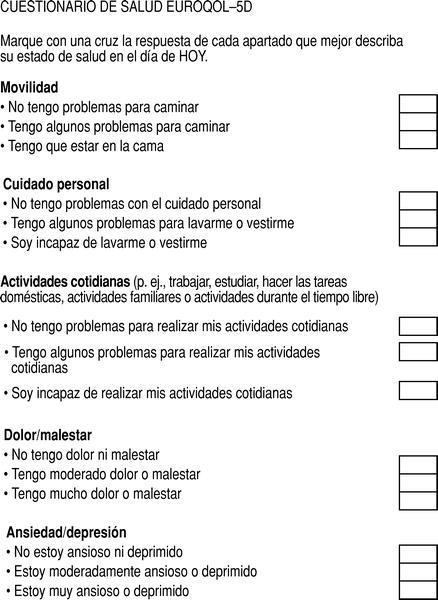
**

**
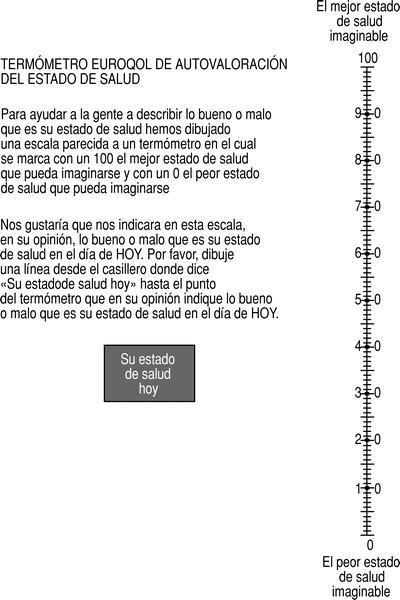
**

**ANEXO III
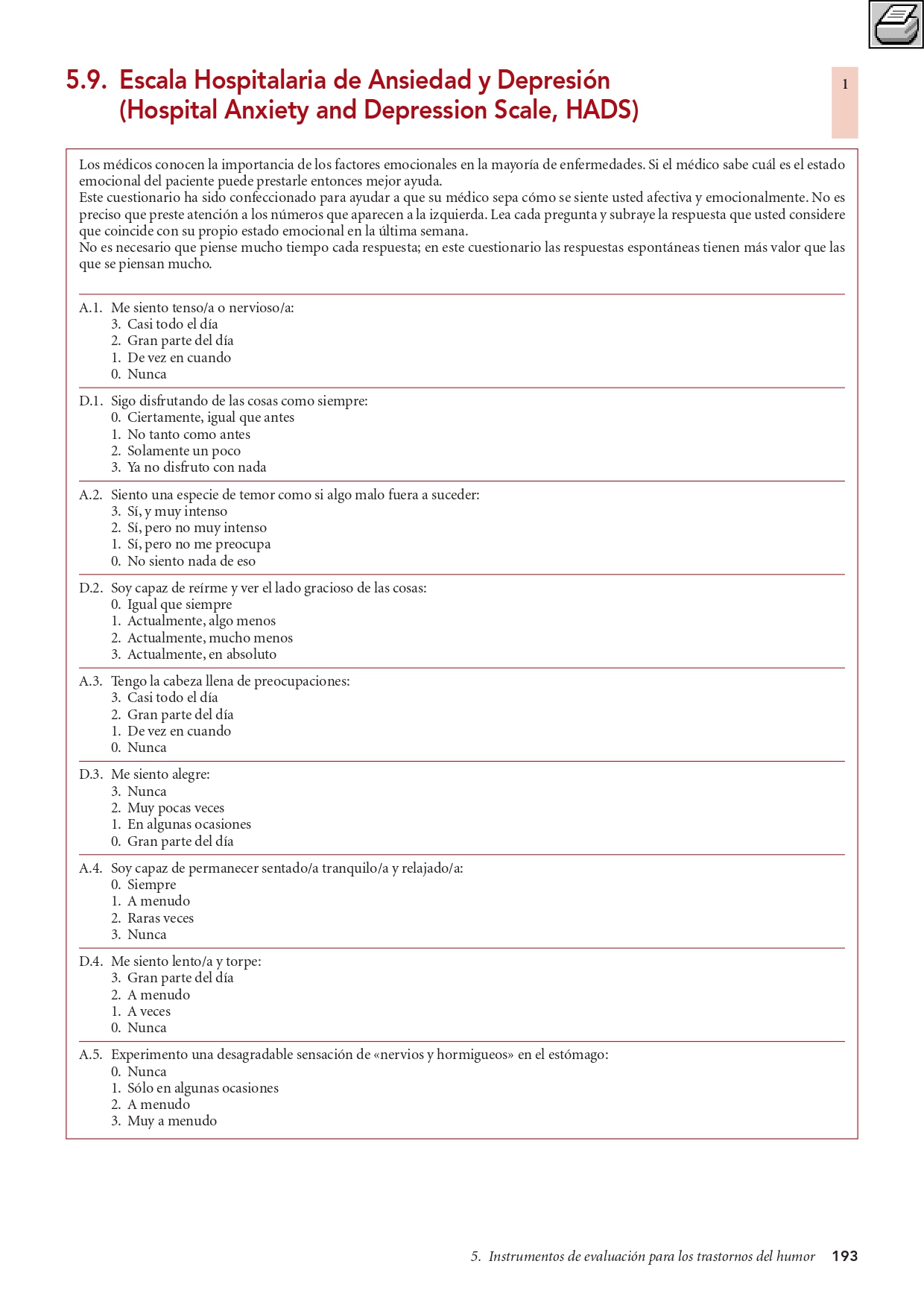
**

**
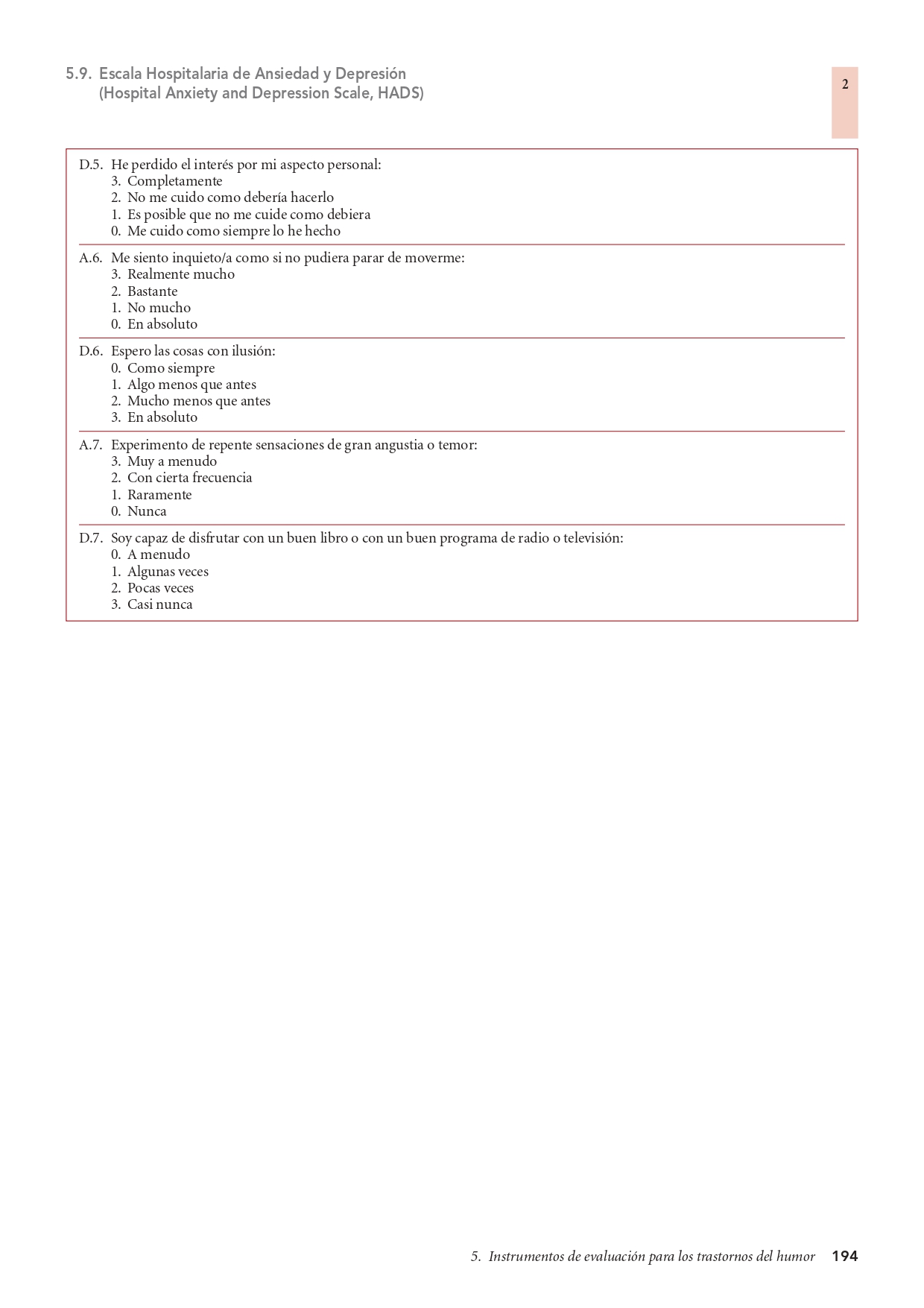
**

**
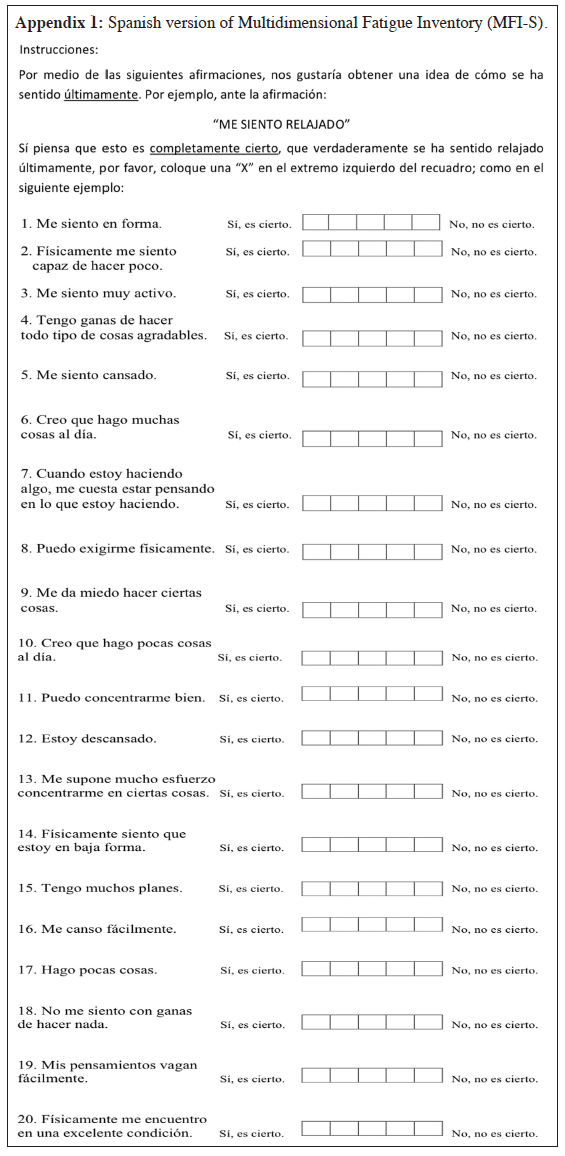
ANEXO IV**

**ANEXO V**

Instrucciones

Las siguientes preguntas se refieren a la forma en que normalmente ha dormido únicamente durante el último mes. Sus respuestas intentarán ajustarse de la manera más exacta a lo ocurrido durante la mayor parte de los días y noches del último mes.

Por favor, conteste a TODAS las preguntas.

1. Durante el último mes: ¿Cuál ha sido, normalmente, su hora de acostarse? _____

2. ¿Cuánto tiempo ha tardado en dormirse, normalmente, las noches, del último mes? _____

3. ¿A qué hora se ha levantado habitualmente por la mañana durante el último mes? _____

4. ¿Cuántas horas calcula que habrá dormido verdaderamente cada noche durante el último mes? (El tiempo puede ser diferente al que usted permanezca en la cama). _____

Para cada una de las siguientes preguntas, elija la repuesta que más se ajuste a su case. Intente contestar a TODAS las preguntas.

5. *Durante el último mes, cuántas veces ha tenido usted problemas para dormir a causa de:*

5a. No poder conciliar el sueño en la primera media hora

_ 0. Ninguna vez en el último mes

_ 1. Menos de una vez a la semana

_ 2. Una o dos veces a la semana

_ 3. Tres o más veces a la semana

5b. Despertarse durante la noche o de madrugada

_ 0. Ninguna vez en el último mes

_ 1. Menos de una vez a la semana

_ 2. Una o dos veces a la semana

_ 3. Tres o más veces a la semana

5c. Tener que levantarse para ir al servicio

_ 0. Ninguna vez en el último mes

_ 1. Menos de una vez a la semana

_ 2. Una o dos veces a la semana

_ 3. Tres o más veces a la semana

5d. No poder respirar bien

_ 0. Ninguna vez en el último mes

_ 1. Menos de una vez a la semana

_ 2. Una o dos veces a la semana

_ 3. Tres o más veces a la semana

5e. Toser o roncar ruidosamente

_ 0. Ninguna vez en el último mes

_ 1. Menos de una vez a la semana

_ 2. Una o dos veces a la semana

_ 3. Tres o más veces a la semana

5f. Sentir frío

_ 0. Ninguna vez en el último mes

_ 1. Menos de una vez a la semana

_ 2. Una o dos veces a la semana

_ 3. Tres o más veces a la semana

5g. Sentir demasiado calor

_ 0. Ninguna vez en el último mes

_ 1. Menos de una vez a la semana

_ 2. Una o dos veces a la semana

_ 3. Tres o más veces a la semana

5h. Tener pesadillas o *malos sueños*

_ 0. Ninguna vez en el último mes

_ 1. Menos de una vez a la semana

_ 2. Una o dos veces a la semana

_ 3. Tres o más veces a la semana

5i. Sufrir dolores

_ 0. Ninguna vez en el último mes

_ 1. Menos de una vez a la semana

_ 2. Una o dos veces a la semana

_ 3. Tres o más veces a la semana

5j. Otras razones. (por favor, descríbalas a continuación): _____

6. Durante el *último mes,* ¿cuántas veces habrá tomado medicinas (por su cuenta o recetadas por al médico) para dormir?

_ 0. Ninguna vez en el último mes

_ 1. Menos de una vez a la semana

_ 2. Una o dos veces a la semana

_ 3. Tres o más veces a la semana

7. Durante el *último mes,* ¿cuántas veces ha sentido somnolencia mientras conducía, comía, o desarrollaba alguna otra actividad?

_ 0. Ninguna vez en el último mes

_ 1. Menos de una vez a la semana

_ 2. Una o dos veces a la semana

_ 3. Tres o más veces a la semana

8. Durante el *último mes,* ¿ha representado para usted mucho problema el *tener ánimos* para realizar alguna de las actividades detalladas en la pregunta anterior?

_0. Ningún problema

_1. Sólo un leve problema

_2. Un problema

_3. Un grave problema

9. Durante el *último mes,* ¿cómo valoraría, en conjunto, la calidad de su sueño?

_ 0. Bastante Buena

_ 1. Buena

_ 2. Mala

_ 3. Bastante mala

**
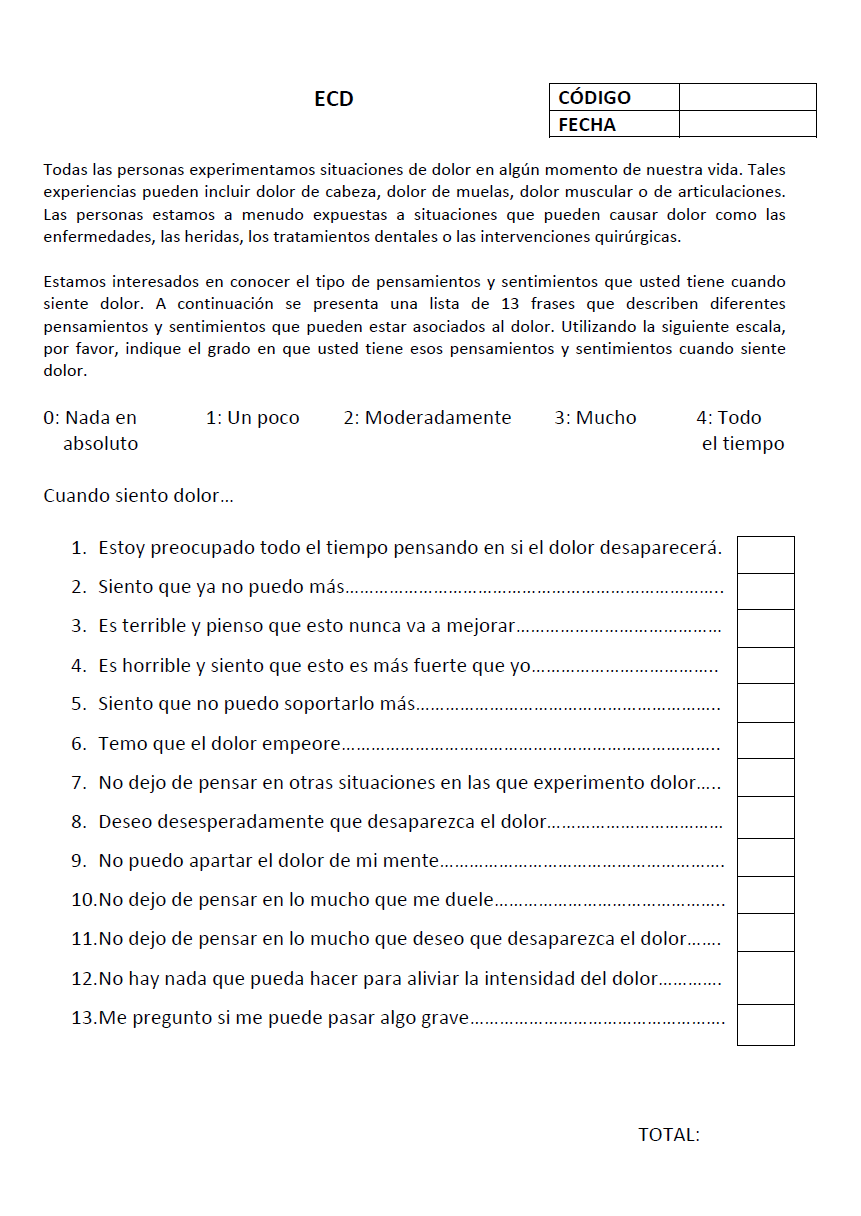
ANEXO VI**

**
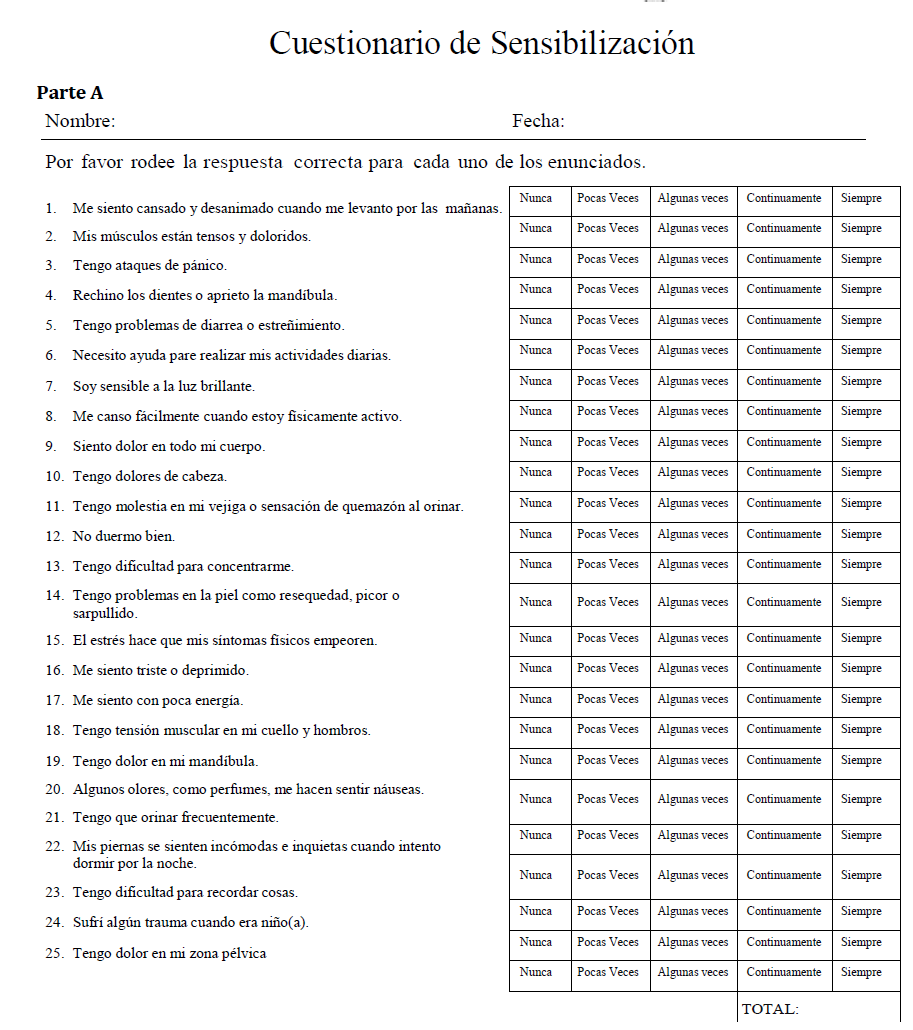
ANEXO VII**

**
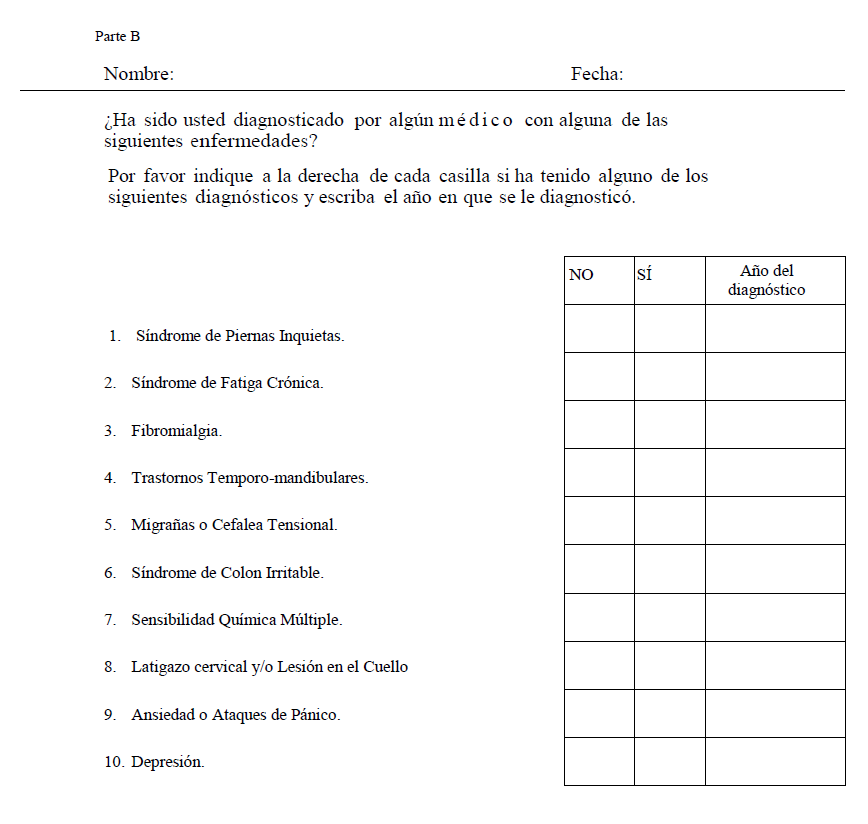
**

**ANEXO VIII**

**
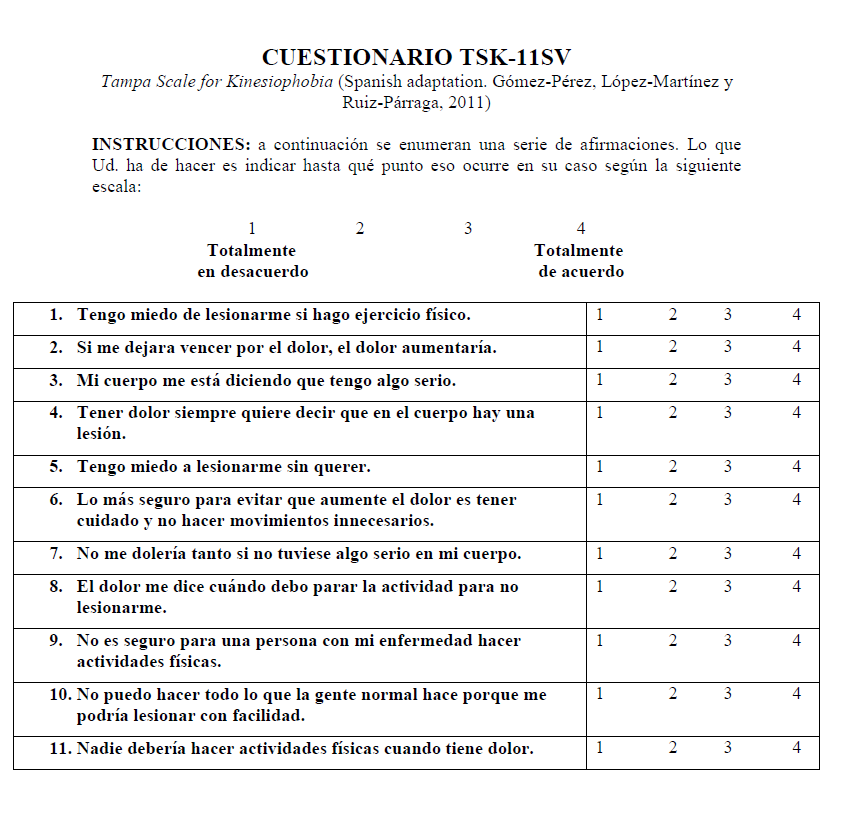
**

**ANEXO IX**

**
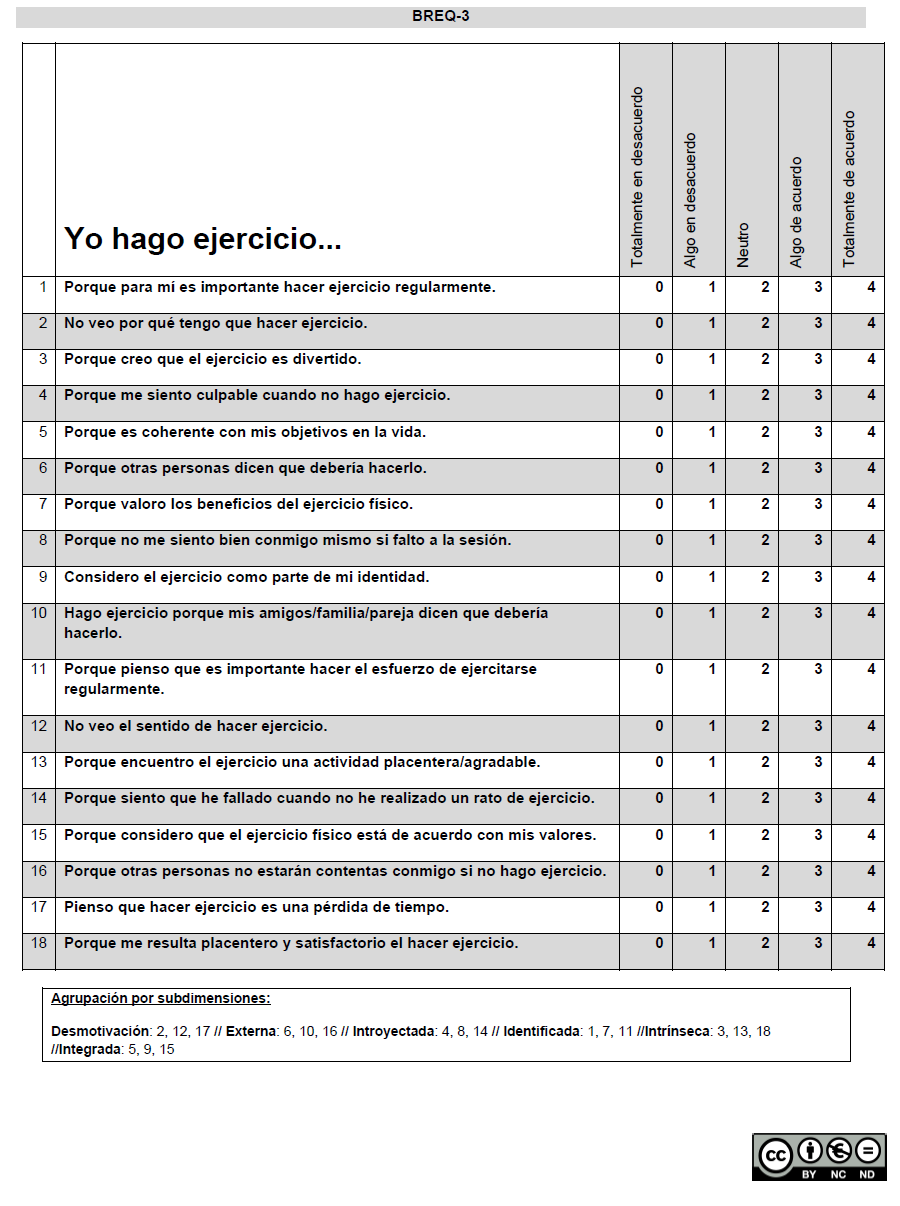
**

**
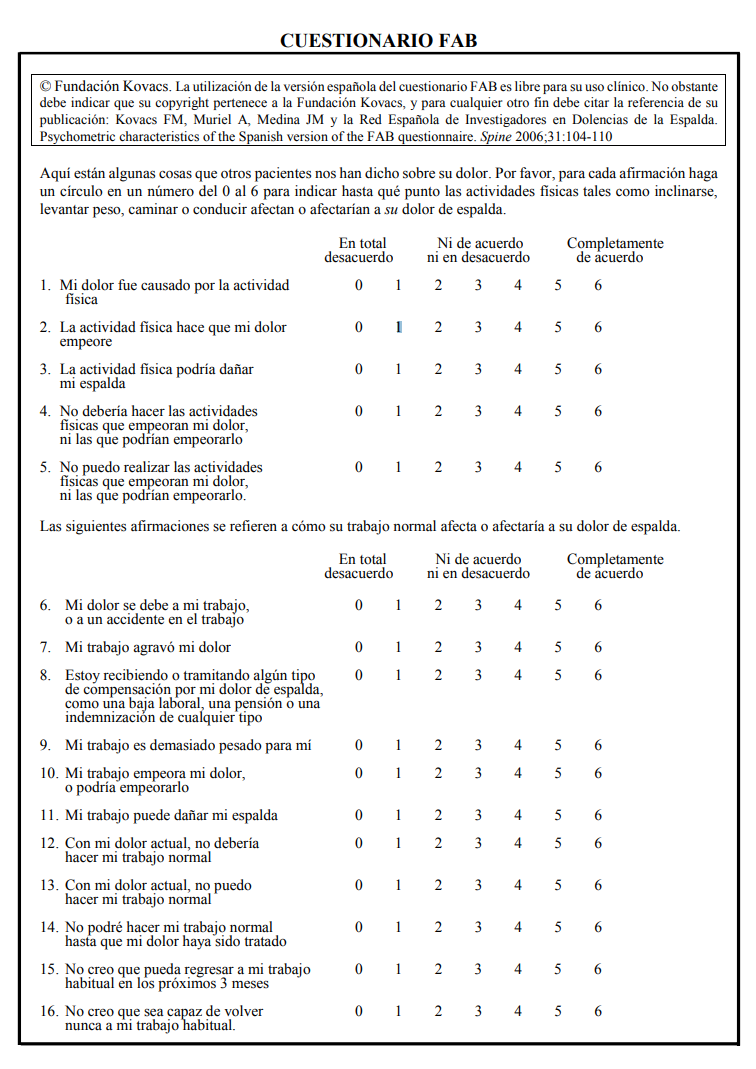
ANEXO X**

**ANEXO X**

**
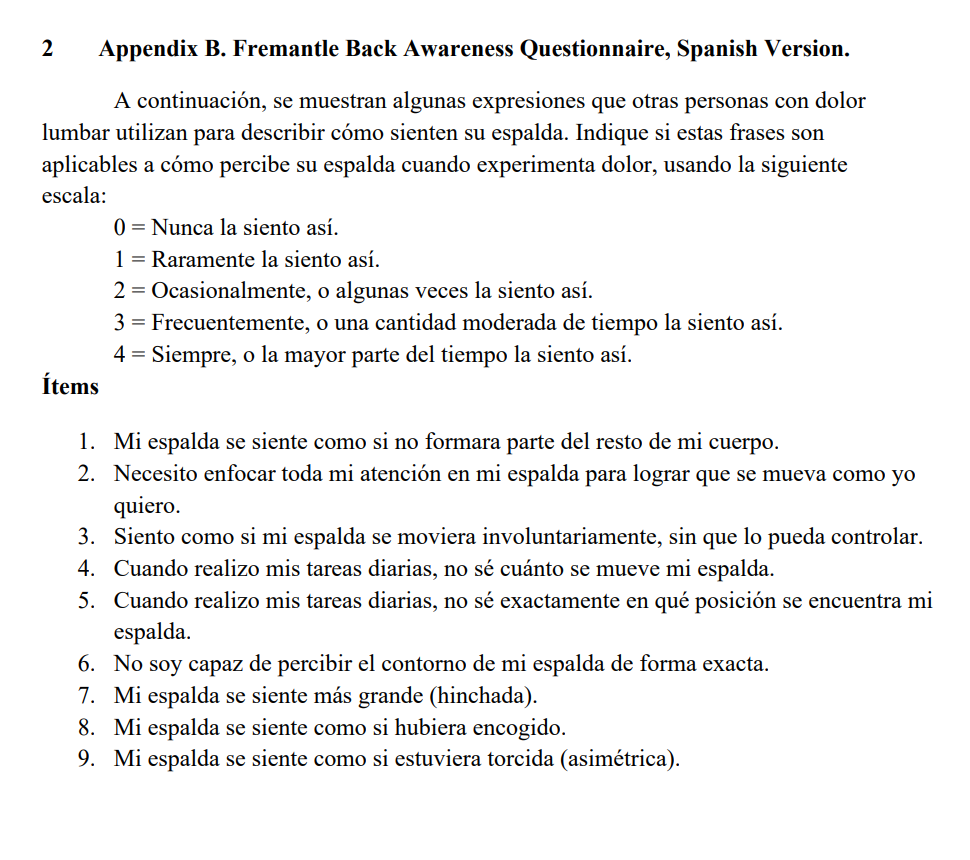
**

**ANEXO XII**

**
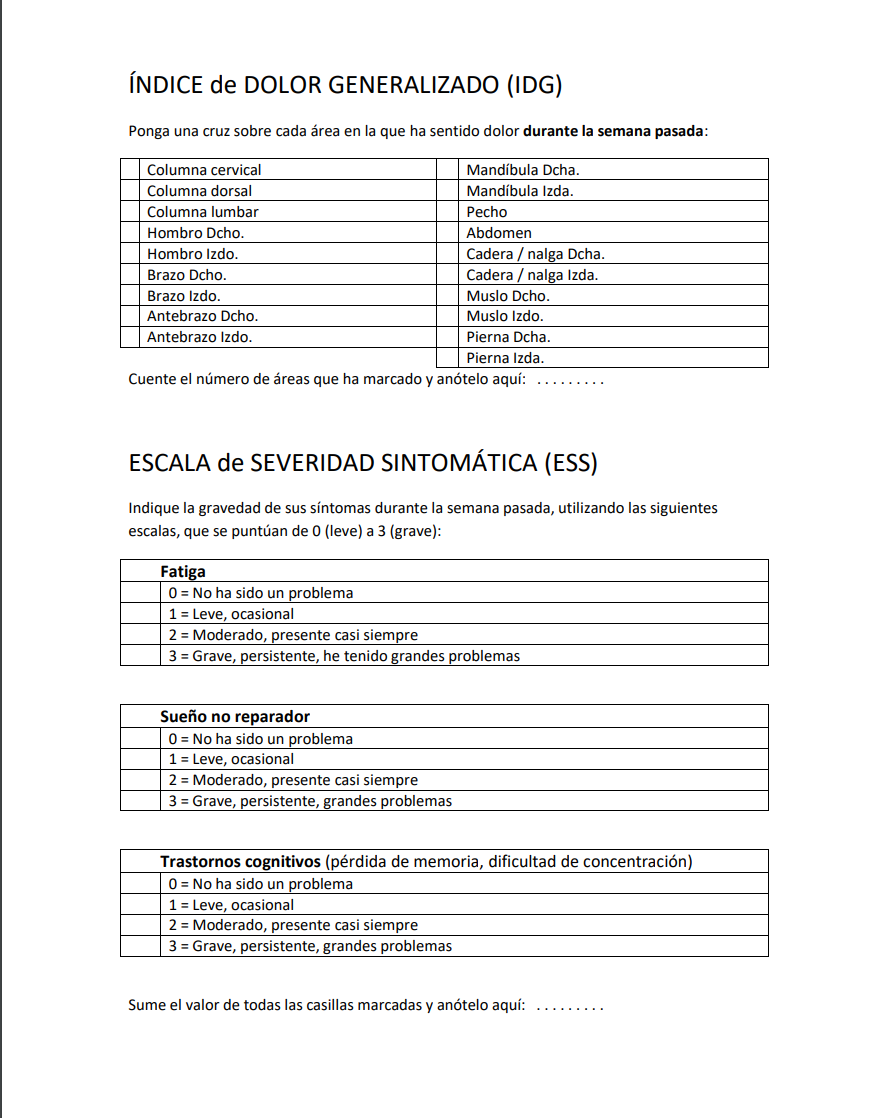
**

**
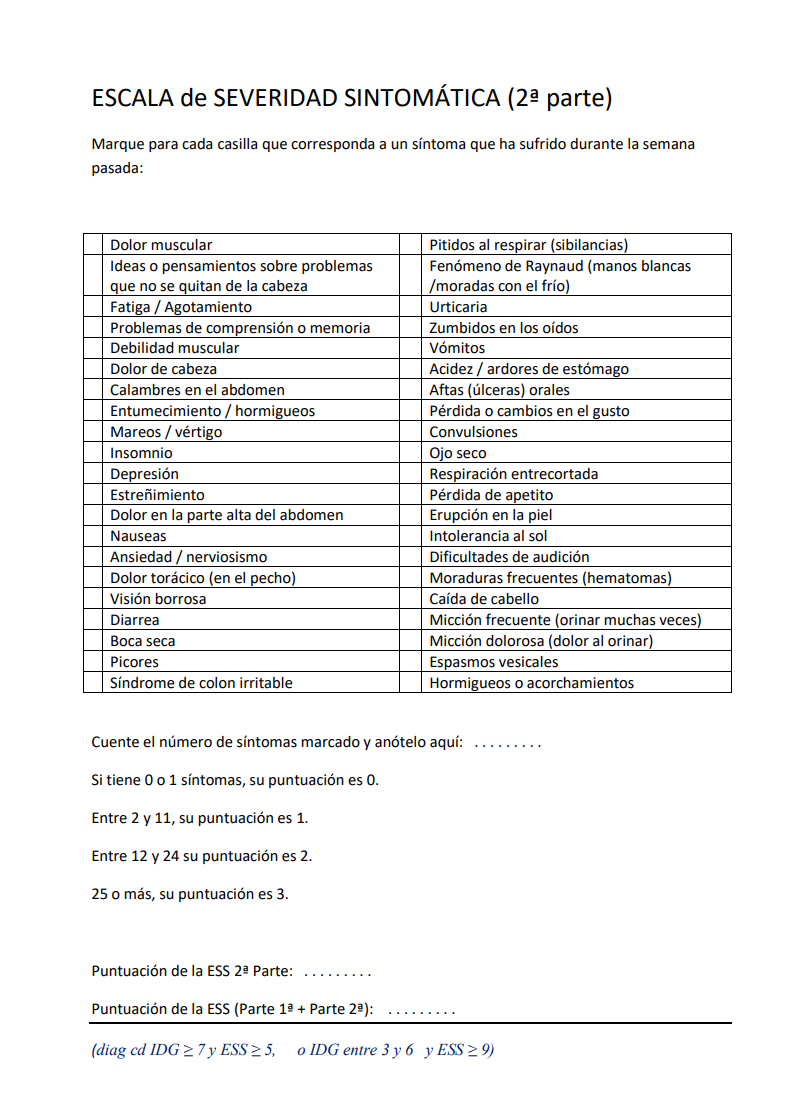
**
