# Supplementary material for: Effectiveness of a fully immersive virtual reality-based therapeutic exercise programme with altered visual feedback in patients with fibromyalgia: A study protocol for a randomised controlled trial
Source: PLoS One. 2026 Jun 4;21(6):e0348346. doi: 10.1371/journal.pone.0348346 (PMC13235889; doi:10.1371/journal.pone.0348346)
Supplement: S2 Protocol — (DOCX) [file pone.0348346.s003.docx]

**Project Report
Human Research Ethics Committee
*(Biosanitary/Humanities-Social Sciences Area)***

**Title:** Efficacy of Immersive Virtual Reality in Patients with Fibromyalgia: A Randomized Clinical Trial

**Supervisors:** Dr. Juan José Amer Cuenca and Dr. Juan Francisco Lisón Párraga

**Student name:** Carlos Salvador Huerta

**1.** **Abstract (200 words)**

Fibromyalgia (FM) is a syndrome characterized by chronic widespread pain, fatigue, and sleep disturbances. Current treatment often relies on pharmacological interventions, whose effectiveness is limited by adverse effects and poor adherence. In contrast, physical exercise has proven beneficial for pain relief and functional improvement. However, persistent pain in FM patients complicates adherence to exercise, necessitating additional complementary strategies. Immersive virtual reality (IVR) has emerged as a promising approach to modulate pain perception through sensory immersion and cognitive distraction. Although IVR has been used in FM through exergames—interactive videogames designed to make physical activity more engaging—its concurrent application with therapeutic exercise remains underexplored. Immersion in virtual environments may positively influence perceived exertion and range of motion, ultimately optimizing the benefits of exercise.

This study aims to evaluate the efficacy of an IVR system that modifies the patient’s visual feedback by creating the illusion of reduced bodily movement during resistance exercises. The hypothesis is that such manipulation may enable greater range of motion without exacerbating pain or perceived effort, thus enhancing the therapeutic outcomes in patients with FM. This innovative approach could pave the way for more effective, patient-centered interventions and promote better adherence to exercise programs.

.

**2. Introduction**

Fibromyalgia (FM) is a condition of unknown etiology, primarily characterized by chronic (>3 months), widespread pain that affects multiple body regions^1^. While the most distinctive clinical feature is widespread pain, FM is a complex, polysymptomatic disease encompassing additional core symptoms, such as fatigue and sleep disturbances^2–4^. Moreover, this condition is commonly accompanied by cognitive dysfunction^3^; regional pain syndromes^5–9^; autonomic alterations^10–16^; psychiatric symptoms^17–19^; and hypersensitivity to external stimuli, as summarized in Figure 1.


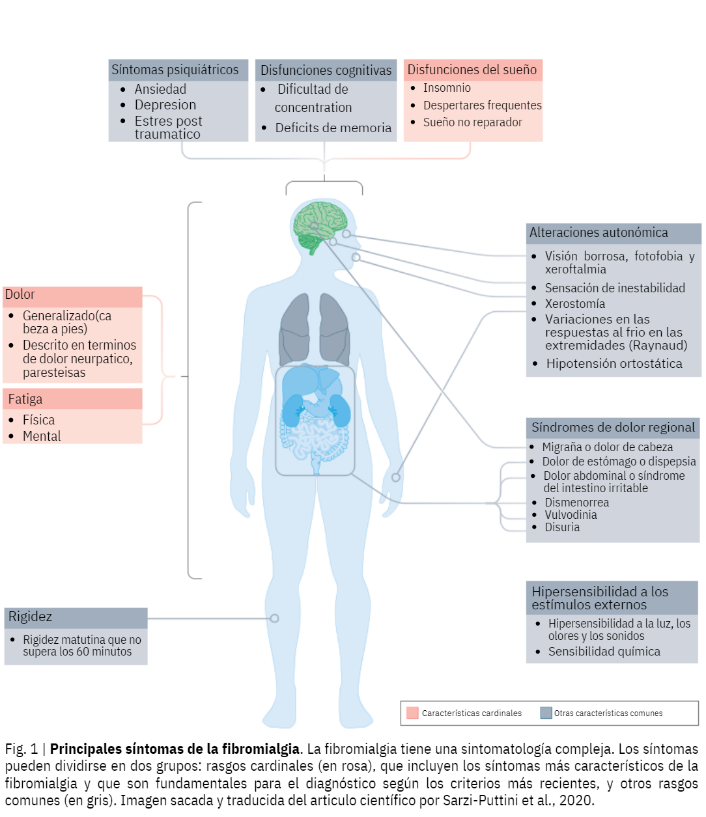


Prevalence estimates of FM vary considerably depending on the diagnostic criteria used. According to the American College of Rheumatology (ACR) 1990 guidelines, rates range from 0.4% to 8.8%, with a global average estimated at 2.7%. In Europe, a multinational study conducted in France, Portugal, Spain, Germany, and Italy reported a prevalence of 4.7% and a female-to-male ratio of 3:1 ^20^. Recognized as the third most common musculoskeletal disorder—after low back pain and osteoarthritis—its frequency increases with age, peaking between 50 and 60 years of age ^21,22^. However, variations in patient-reported data and insufficient clinical recognition may affect the precision of these estimates ^23^.

The quality of life among individuals with FM is severely compromised by the characteristic symptoms of the disease, as evidenced by the high healthcare costs it incurs. The annual number of medical visits is nearly double that of healthy controls, and total healthcare expenditures are roughly three times higher ^24,25^. Indirect societal costs are also substantial, primarily due to reduced work productivity ^26,27^; one study found that 24.3% of patients were no longer working five years following FM onset ^28^. Furthermore, healthcare expenditures are directly related to FM symptom severity ^29^, underscoring the need for effective and sustainable therapeutic strategies. Although pharmacological treatments can provide symptom relief, they are often associated with a high incidence of adverse effects and lower patient adherence^30^.

By contrast, physical exercise (PE) has become a well-documented, effective therapeutic tool for managing FM. Multiple studies have demonstrated its benefits, including pain reduction, enhanced quality of life, increased physical capacity, and decreased depressive symptoms. Particularly effective modalities include resistance training ^31–36^ and aerobic exercise ^31,32,37–39^, both of which are widely recommended for FM management. Nevertheless, considering the complex and multidimensional nature of chronic pain, additional complementary strategies have been explored.

In this regard, virtual reality (VR) has emerged as an innovative approach with significant therapeutic potential. Recent evidence suggests that VR can modulate pain perception through sensory immersion, cognitive distraction, and multisensory stimulation, making it a promising technique for improving pain control in FM patients ^40,41^. VR technologies are typically classified into three categories based on their immersion level: non-immersive, semi-immersive, and fully immersive. The degree of immersion has a direct impact on the user’s perceptual and psychological experience, as well as their sense of embodiment within the virtual environment ^42^.

In the context of FM treatment, non-immersive VR devices, often in the form of exergames, have been the most frequently employed. Despite offering a limited degree of immersion, these approaches have shown efficacy in improving critical variables in FM populations, including disease impact, pain, dynamic balance, aerobic capacity, fatigue, quality of life, anxiety, and depression ^43–53^. Conversely, immersive virtual reality (IVR) offers a substantially higher level of immersion ^42,54^. This modality not only enhances the user’s sense of embodiment but can also deliver real-time feedback^55^. Studies indicate that higher immersion quality correlates with stronger pain distraction effects, wherein greater immersion leads to more pronounced pain reduction compared to less immersive approaches ^56^.

IVR has thus emerged as an innovative therapeutic tool, not only for modulating pain perception but also for improving functionality in patients with FM ^57–59^. While extensive research has examined IVR in the context of exergames or as an adjunct after exercise, its simultaneous application during therapeutic exercise remains underexplored. Furthermore, the potential benefits of manipulating visual feedback in an IVR environment during a therapeutic exercise program for FM have yet to be thoroughly investigated.

To our knowledge, no existing studies have evaluated the impact of a therapeutic exercise program incorporating IVR with modified visual feedback on FM symptoms. Therefore, the primary goal of the present study is to determine the efficacy of an IVR system that adjusts the patient’s visual perception, creating the illusion of reduced bodily movement compared to actual movement during resistance training. This manipulation may enable an increased range of motion without heightening perceived effort or pain, thereby optimizing overall treatment efficacy.

**3.Objectives**
**Objective 1:** Evaluate the effectiveness of immersive virtual reality combined with resistance exercises in alleviating fibromyalgia-related pain in patients’ daily lives.
**Objective 2:** Determine the capacity of immersive virtual reality combined with resistance exercises to improve the quality of life and functional capacity of patients with fibromyalgia.
**Objective 3:** Examine the effectiveness of immersive virtual reality combined with resistance exercises in bringing about changes in the degree of central sensitization among fibromyalgia patients.

**4. Methodology:**

**4.1 Study Design**
This Randomized Clinical Trial (RCT) is designed to evaluate the efficacy of immersive virtual reality in patients with fibromyalgia. Participants will be randomly assigned to two groups. The intervention group will receive therapeutic exercise combined with immersive virtual reality, while the control group will perform only resistance exercise. The goal is to determine whether immersive virtual reality leads to a significant improvement in outcomes for the intervention group compared to the control group.

**4.2 Target Population and Study Population**
Adults of both sexes, aged 18 or older, diagnosed with fibromyalgia (according to ACR 1990, 2010, 2011 and 2016 criteria).

**4.3 Selection Criteria**
The selection criteria for this study follow the standardized guidelines commonly used in research involving therapeutic exercise and/or immersive virtual reality for patients with fibromyalgia.

Participants will be included if they meet the following requirements: adults of both sexes, over 18 years of age, diagnosed with fibromyalgia based on any of the American College of Rheumatology (ACR) criteria (1990, 2010, 2011 or 2016), able to communicate with the study staff, willing to participate by signing the informed consent form, and reporting a perceived pain score of 3 or higher on a 11-point Numerical Pain Rating Scale (NPRS-11)

Exclusion criteria include the presence of additional conditions and/or symptoms contraindicating immersive virtual reality and exercise, pathologies that could interfere with study outcomes (e.g., visual, auditory, perceptual, or sensory disorders), the use of medication that could affect study results, concurrent therapeutic physical activity treatment during the intervention, or any changes to usual treatments—be they pharmacological or physical therapies—during the intervention.

**4.4 Sample size calculation**

The primary outcome is FIQR. The between-group MCID is ≈14% of the FIQR total score. Assuming SD=20, α=0.05 and power=80% with ANCOVA adjusted for baseline, 32 per group are required (total 64). Allowing 25% attrition, the target sample size is 80.

**4.5 Participant Recruitment Procedure**

Participant recruitment will be conducted in collaboration with the Valencian Association of Fibromyalgia Patients (AVAFI), which will disseminate pertinent information via various media to facilitate volunteer enrollment. In addition to a detailed explanation of the research, a specific time will be allotted to address any questions and provide individualized consultations. This approach ensures that potential participants have a thorough understanding of the study and the intervention before making an informed decision about participating

**4.6 Type of Sampling and Randomization**

Participant recruitment and written informed consent will be obtained by physicians from the “Estilo de vida y salud” research group at CEU Cardenal Herrera University. Randomisation will be conducted by an independent researcher using a computer-generated 1:1 sequence with stratified permuted blocks of random sizes (2–4) within eight strata defined by sex (male/female) and FM severity (four FIQR clusters per Pérez-Aranda et al., 2019 [32]). Allocation will be concealed with sequentially numbered, opaque, sealed envelopes (SNOSE) prepared off-site with tamper-evident seals and carbonless copies. After baseline assessment, site staff will open the next envelope in numerical order to assign the experimental group (EG) or control group (CG). The randomisation list will be held by an independent data manager with no role in enrolment or assessment. Any deviation from sequence or envelope integrity will be recorded and investigated. Outcome assessors and the trial statistician will be blinded to group allocation. Participants and intervention providers cannot be blinded due to the nature of immersive VR and will be instructed not to disclose allocation during assessments. Assessments will be performed by personnel without access to scheduling or allocation logs; groups will be labelled A/B until database lock. Unblinding will occur only to address serious safety concerns or protocol deviations with safety implications, following written authorization from the Principal Investigator; all unblinding events will be logged. The allocation list will remain with the independent data manager.

**4.7 Type of Blinding**

This study will be conducted as a single-blind design, in which the evaluators are unaware of participants’ group assignments. This approach minimizes detection bias, thereby ensuring objective outcome evaluation and enhancing the study’s validity.

**4.8 Intervention**

The intervention programme will last a total of six weeks, with two weekly sessions of 60 minutes each. Sessions will take place in AVAFI’s facilities, in a climate-controlled room that meets the necessary space and privacy requirements. Each session will be led by a researcher responsible for ensuring proper exercise execution and participant well-being. The session will be divided into three distinct phases:

- **Warm-Up Phase (5 minutes):**

Joint mobility exercises combined with breathing techniques to prepare the body for physical activity. These exercises will focus on the cervical and lumbopelvic areas and the upper and lower extremities, without additional load.

- **Main Intervention Phase (50 minutes):**

Participants will carry out a therapeutic exercise programme tailored to their group (intervention or control), with intensity regulated through the Borg Scale (6–20). Sessions will be supervised by a researcher to ensure proper exercise execution and participant safety. Exercises are illustrated in Figure 2 and will include trunk flexion, extension, rotation, and side bending in various positions (standing, seated, and lying down). Participants will be instructed to synchronize the concentric and eccentric phases with their breathing to maintain a consistent execution speed.
The programme will focus on exercises using body weight, and starting from the sixth session, ankle weights and some dumbbell exercises will be added. Participants will perform between 1 and 2 sets of each exercise, adjusting intensity and repetitions according to their perceived exertion, assessed using the Borg Scale (6–20), aiming for an intensity range between 13 and 17. During each session, pain intensity (NPRS-11), perceived exertion (RPE), and well-being associated with Virtual Reality will be recorded.

- **Cool-Down Phase (5 minutes):**

Stretching and breathing exercises to reduce muscle tension and promote relaxation. These will include stretches for the lumbar region and lower extremities, along with deep breathing exercises performed in a supine position.

**Intervention Group: Virtual Reality + Exercise**

Participants assigned to this group will perform the aforementioned therapeutic exercise programme while simultaneously using an immersive virtual reality device (HTC Vive Pro). The VR system will modify the patient’s visual and proprioceptive information, allowing them to reduce the perception of movement, thereby improving pain tolerance and body awareness.

**Control Group: Exercise Without Virtual Reality**

Participants in the control group will follow the same therapeutic exercise programme as the intervention group but without using virtual reality technology. The exercises will be identical in terms of type, progression, and load.

 ***Figure 2: Exercise programme with virtual reality.*** *1) Trunk flexion in standing position; 2) Trunk extension in sitting position; 3) Trunk flexion in sitting position; 4) Glute bridge; 5) Crunch; 6) Trunk inclination in standing position; 7) Trunk rotation in lying position 8) Trunk extension in lying position.*

***Figure 3:*** *VR set up and virtual environment.*

4.9 Outcomes and measurement instruments

1. The FIQR is a validated, widely used instrument for assessing the impact of fibromyalgia on patients’ daily lives. It evaluates three key dimensions: functionality (9 items, 0–30 points), symptoms (10 items, 0–50 points), and overall impact (2 items, 0–20 points), yielding a total score from 0 to 100. Higher scores reflect a greater impact of the disease. Each item is rated from 0 to 10, indicating no impairment to maximum impairment. The Spanish version of the FIQR has demonstrated high reliability (Cronbach’s α = 0.91–0.95) and is considered the reference instrument for evaluating the effectiveness of fibromyalgia interventions, given its sensitivity to detect change and its strong support in the scientific literature ^61,62^.
2. The EQ-5D-5L is a validated questionnaire translated into Spanish for assessing quality of life. Developed by the EuroQol Group as an enhancement to the EQ-5D-3L, it evaluates five key dimensions—mobility, self-care, usual activities, pain/discomfort, and anxiety/depression—using five response levels (1 = best health state, 5 = worst health state). It also includes a visual analog scale (VAS) from 0 to 100 for rating overall health status on the day of evaluation. Test-retest reliability ranges from 0.69 to 0.94 (ICC) ^63^, and it is widely employed in fibromyalgia and chronic pain studies for its capacity to provide an objective, comparative assessment of quality of life ^44,51,63–66^.
3. The participants’ levels of anxiety and depression will be evaluated using the Hospital Anxiety and Depression Scale (HADS), a validated instrument for both clinical and non-clinical populations. It consists of 14 items divided into two subscales—Anxiety (HADS-A) and Depression (HADS-D)—each scored from 0 to 21, where higher scores indicate greater severity. The Spanish version has shown high reliability (Cronbach’s α = 0.83–0.86 for anxiety and 0.82–0.84 for depression; ICC = 0.76–0.93) and approximately 80% sensitivity and specificity. In patients with fibromyalgia, it has demonstrated significant concurrent validity with measures of pain and quality of life, making it an appropriate instrument for this population since it evaluates affective symptoms without confounding somatic symptoms of the disease. Given that fibromyalgia is associated with high rates of anxiety and depression, assessing these factors is crucial for understanding the emotional impact of the condition and determining the effectiveness of therapeutic interventions ^69,70^.
4. Sleep quality will be assessed using the Spanish version of the Pittsburgh Sleep Quality Index (PSQI), a validated instrument for measuring sleep quality and patterns over the past month. It consists of 19 items grouped into seven components, yielding a global score from 0 to 21, where higher scores indicate poorer sleep quality. The Spanish version has demonstrated high reliability (α = 0.805; test-retest r = 0.773, p < 0.001) and convergent validity with the FIQR and SF-36. Its use is widely supported in fibromyalgia research, serving as a sensitive tool for detecting sleep disturbances in this population^71,72^.
5. Pain catastrophizing will be evaluated using the Pain Catastrophizing Scale (PCS), a questionnaire designed to measure the tendency toward negative, exaggerated thoughts in response to pain. It consists of 13 items that assess rumination, magnification, and helplessness, each scored from 0 (never) to 4 (always), yielding a total score of 0 to 52, where higher scores indicate greater catastrophizing. The Spanish version has demonstrated high reliability (Cronbach’s α = 0.79; test-retest ICC = 0.84) and retains the original three-factor structure. It has also shown sensitivity to change with an effect size of up to 2, making it a valid and reliable tool in fibromyalgia and chronic pain research. Additionally, it is useful for predicting pain intensity, disability, and response to therapeutic interventions ^73–75^.
6. Central sensitization will be assessed using the Central Sensitization Inventory (CSI), an instrument designed to identify symptoms related to central sensitization and their impact on daily life. It consists of 25 items evaluating widespread pain, fatigue, concentration problems, sensory hypersensitivity, and sleep disturbances, yielding a total score from 0 to 100. Higher scores indicate a greater burden of symptoms, and scores above 40 are considered indicative of a central sensitization syndrome such as fibromyalgia. The Spanish version has shown high test-retest reliability (ICC = 0.82–0.91) and is widely used in chronic pain research, providing an effective measure of central sensitization in various clinical populations ^76^.
7. Nervous system function will be evaluated through Quantitative Sensory Testing (QST), a set of standardized assessments that measure sensory perception and pain modulation using mechanical, thermal, and pressure stimuli. QST examines key parameters such as detection and tolerance thresholds for pressure pain, thermal detection (heat/cold), and the presence of mechanical and thermal hyperalgesia or allodynia, thus distinguishing between peripheral and central sensitization. Test-retest reliability varies by parameter (ICC = 0.75–0.95), and QST has been validated in fibromyalgia and chronic pain studies. It is a critical tool for characterizing dysfunctions in pain modulation and for assessing the effectiveness of therapeutic interventions ^77,78^.
8. Kinesiophobia will be evaluated using the Tampa Scale for Kinesiophobia (TSK), a questionnaire developed to measure fear of movement and avoidance of physical activity in individuals with chronic pain. The Spanish version has been validated in a reduced 11-item format (TSK-11), assessing two factors: Avoidance of Activity (AA) and Harm (H). Each item is rated on a scale from 1 (strongly disagree) to 4 (strongly agree), yielding a total score ranging from 11 to 44, where higher values reflect greater kinesiophobia. Its test-retest reliability is moderate to high (Cronbach’s α = 0.79–0.81; ICC = 0.55–0.91) and shows significant associations with catastrophizing, anxiety, and depression. The TSK-11 is widely used in fibromyalgia and chronic pain research, serving as a key instrument to evaluate the impact of fear of movement on functionality and adherence to exercise-based treatments ^58,79,80^.
9. Lower-limb strength and endurance will be assessed using the 30-Second Sit-to-Stand Test (STS-30), a functional test that measures one’s ability to rise repeatedly from a chair over 30 seconds without using the arms. It is widely used to evaluate muscular strength, endurance, and functionality in individuals with chronic pain and reduced mobility. The total number of repetitions completed reflects functional performance, with higher scores indicating greater capacity. Test-retest reliability is high (ICC = 0.84–0.96), and the Spanish version has been validated in fibromyalgia and aging studies, making it essential for assessing functionality and progression in exercise-based intervention programmes ^81–83^.
10. Functional mobility will be assessed using the Timed Up and Go (TUG) Test, a widely used measure of walking speed, dynamic balance, and functional capacity in individuals with fibromyalgia. It involves timing how long it takes a participant to stand from a chair, walk 3 meters, turn 180 degrees, walk back, and sit down again. Its test-retest reliability is high, with an ICC of 0.935 when using a manual stopwatch and 0.955 when using an automatic device; the latter is recommended to improve precision and reduce variability in measurement. The TUG has been previously evaluated and validated in fibromyalgia patients and serves as a key tool for assessing clinical progression and the response to exercise-based therapeutic interventions ^46,84,85^.
11. Isometric quadriceps strength will be evaluated using dynamometry, a technique widely employed to quantify neuromuscular function in individuals with fibromyalgia. A portable dynamometer will be placed at the ankle with the knee flexed at 90° to measure the maximum voluntary isometric contraction of the quadriceps muscle. In fibromyalgia patients, a significant reduction in muscle strength has been reported, potentially associated with fatigue, chronic pain, and decreased functional capacity. Quadriceps strength measurement is a key indicator of lower-limb functionality and disability risk in this population. The test-retest reliability of isometric dynamometry in fibromyalgia ranges from 0.85 to 0.96 (ICC), and its use has been validated in previous studies, providing an objective assessment of muscular response to exercise-based therapeutic interventions.
12. Handgrip strength will be assessed using manual dynamometry, a validated method for measuring isometric hand strength and a clinically relevant marker in individuals with fibromyalgia. Three maximal attempts will be conducted for each limb using a handheld dynamometer, following standardized protocols with the elbow flexed at 90° and the forearm in a neutral position. In fibromyalgia patients, handgrip strength can be reduced by up to 40% compared to healthy individuals, which correlates with decreased functionality, widespread pain, and fatigue. This reduction also reflects potential neuromuscular dysfunction and central sensitization—key features of fibromyalgia. Test-retest reliability is high (ICC = 0.85–0.98), and the measure has been validated in previous studies, serving as a key tool for monitoring changes in functionality and evaluating therapeutic intervention outcomes ^82,86^.
13. Exercise behavior regulation will be assessed using the Behavioral Regulation in Exercise Questionnaire-3 (BREQ-3), an instrument grounded in Self-Determination Theory that evaluates various types of motivation for exercise. It consists of 24 items distributed across six dimensions, allowing analysis of the degree of self-determination in physical activity. The Spanish version has been validated, demonstrating satisfactory reliability (Cronbach’s α = 0.81 for intrinsic regulation and 0.70 for amotivation) and a solid factorial structure, supporting its construct validity. Among individuals with fibromyalgia, motivation is critical for exercise programme adherence, influencing functionality and quality of life. Consequently, BREQ-3 is a fundamental tool for assessing these factors in the present study ^87^.
14. Participants’ experience with virtual reality (VR) will be evaluated using NPRS-11scales for nausea intensity and satisfaction, ensuring the intervention’s tolerability and acceptance. VR has demonstrated efficacy as a tool in chronic pain management, including fibromyalgia, and its use in this study will be monitored to ensure participants’ comfort. Additionally, an NPRS-11will be employed to measure perceived pain before and after each session, providing an objective assessment of treatment impact.
15. Fear-avoidance beliefs regarding pain will be evaluated using the Fear-Avoidance Beliefs Questionnaire (FABQ), a widely used instrument in fibromyalgia and chronic pain studies to measure the impact of fear on physical activity and work. It comprises 16 items divided into two subscales: physical activity (FABQ-PA) and work (FABQ-W), with responses scored from 0 (strongly disagree) to 6 (strongly agree). In individuals with fibromyalgia, higher FABQ scores have been associated with increased disability, greater pain, and lower adherence to exercise programmes. The Spanish version has been validated, demonstrating high test-retest reliability (ICC = 0.84 for FABQ-W and 0.80 for FABQ-PA) and good internal consistency (Cronbach’s α = 0.88 for FABQ-W and 0.79 for FABQ-PA). Moreover, factorial analysis confirmed its bidimensional structure, further consolidating its utility as a key tool for assessing the psychological impact of pain on functionality and recovery ^88^.
16. The extent of pain and symptom severity will be evaluated using the Widespread Pain Index (WPI) and the Symptom Severity Scale (SSS), instruments used in the diagnosis and monitoring of fibromyalgia in accordance with the American College of Rheumatology (ACR) criteria. The WPI measures the distribution of pain across 19 body regions (scored 0–19), while the SSS assesses the severity of symptoms such as fatigue, sleep disturbances, and cognitive problems (scored 0–12). Both instruments will be administered before and after the intervention to assess the treatment’s impact on pain distribution and symptom severity.
17. Body perception will be evaluated using the adapted version of the Fremantle Body Awareness Questionnaire (FBAQ-B), an instrument designed to identify distortions in body representation—a phenomenon documented in individuals with fibromyalgia. These distortions include sensations of disconnection, changes in body shape or size, and difficulty localizing body parts, which are linked to dysfunctions in somatosensory integration and central sensitization. The Spanish version of the FBAQ-B has been validated, demonstrating a unidimensional structure, high reliability (α = 0.82), and good test-retest reliability (ICC = 0.78), as well as both discriminant and convergent validity, thus effectively differentiating between patients and controls. Given that virtual reality (VR) has the potential to modulate these mechanisms by providing an immersive environment that enhances body awareness, its impact will be analyzed before and after the intervention. Evaluating these changes will help determine whether combining VR with therapeutic exercise improves sensory integration and reduces perceptual distortions, thereby providing novel insights into its potential in fibromyalgia management ^89,90^.

**4.10 Information on the Proposed Data Analysis**

All analyses will follow the intention-to-treat principle.

Primary outcome (FIQR). Intervention effects will be assessed using a two-way repeated-measures ANCOVA with one within-subject factor (Time: t0, t1) and one between-subject factor (Group: IVR+exercise vs exercise). The dependent variable will be the post-intervention score (t1), adjusted for its baseline value (t0). Sex and baseline FIQR will be included as prespecified covariates. Results will be reported as adjusted mean differences with 95% confidence intervals. Statistical significance will be set at two-sided p<0.05.

Secondary outcomes will be analyzed using the same ANCOVA framework (t1 adjusted for t0), with Group as the between-subject factor and the same covariates (sex and baseline FIQR). Within-group standardized effect sizes (Cohen’s d) will be presented descriptively.

All analyses will be performed in SPSS v27.0 (IBM Corp., Armonk, NY, USA).

**5. Data Processing:**

All data processing will comply with the General Data Protection Regulation (RGPD 2016/679) and the Organic Law on Data Protection and Guarantee of Digital Rights (LOPD-DGG).

**5.1 Data Collection and Storage**

Data will be collected in person by trained assessors at t0 and t1. Paper forms will be double-entered into a password-protected electronic database. Personally, identifying information (PII) will be recorded only on consent forms and a separate offline linkage log stored in a locked cabinet. De-identified analysis datasets will be retained and shared per the Data Availability plan (Zenodo DOI), while the linkage log will be destroyed within one month after database lock.

**5.2 Identification of the Data Controller**

**PII will be collected on consent forms and in a separate linkage log kept offline; the research dataset for analysis will be fully de-identified.**

**5.3 Timing of Data Processing**

Data will be recorded at two distinct time points (t) throughout the study:

- t₀: Before the intervention
- t₁: Immediately after the intervention

All data will be stored and processed for the strictly necessary duration to complete the study, following these phases:

- **Data Collection and Processing:** Conducted during the intervention period of the study, ensuring that only authorized personnel have access to the information.
- **Storage and Analysis:** Data will be maintained for the time required for analysis and subsequent publication of results, with custody secured through appropriate security measures.
- **Completion and Disposal:** Once the study is concluded and legal retention periods have expired, the data will be anonymized or securely destroyed in accordance with current regulations.

At all times, participants will have the right to access, rectify, restrict, or delete their data as provided by data protection legislation.

**5.5 Participants Involved in Each Phase**

Study participants will be adults diagnosed with fibromyalgia, selected based on the established inclusion and exclusion criteria. Prior to participation, they will receive a detailed information sheet and will sign an informed consent form after any questions have been addressed.
Continuous monitoring will be ensured to observe any potential adverse effects and to safeguard participant well-being. Moreover, participants may withdraw from the study at any time without repercussions.
This procedure guarantees adherence to ethical and methodological standards, ensuring the validity and security of the study.

**5.6 Locations Where Data Will Be Stored**

Online data will be stored in encrypted Excel files by the principal investigator on one of the research group's computers, secured with a password. Paper records will be kept in a designated locker for this study, which will remain locked.

**6. Ethical Considerations**

This study will adhere to the CONSORT guidelines for Randomized Controlled Trials and will be evaluated by the Ethics Committee of CEU Cardenal Herrera University, ensuring compliance with all necessary ethical directives. In addition, the study will follow the principles outlined in the Declaration of Helsinki (2013) and Good Clinical Practice (GCP) standards, thereby ensuring the respect, safety, and well-being of the participants.

Fibromyalgia is a chronic condition that significantly impacts the quality of life of those affected. Consequently, any therapeutic intervention must be carefully designed to maximize benefits while avoiding unnecessary risks. In this study, both immersive virtual reality (IVR) and therapeutic exercise have been extensively researched and demonstrated to be safe in similar populations. Nevertheless, potential transient effects—such as mild fatigue or slight dizziness related to IVR—will be closely monitored to guarantee participant well-being.

All study subjects will receive detailed information about the objectives, methodology, potential benefits, and risks of the intervention. Informed consent will be obtained prior to participation, ensuring that individuals make a free and fully informed decision. Furthermore, participants will have the right to withdraw from the study at any time without any negative consequences.

Data processing will be conducted in full compliance with the Organic Law on Data Protection and Guarantee of Digital Rights (LOPD-GDD) and the General Data Protection Regulation (RGPD 2016/679), thereby ensuring both confidentiality and anonymity. The data will be securely stored on CEU Cardenal Herrera University’s servers, with access restricted exclusively to the principal investigators. Upon completion of the study, the data will be disposed of in accordance with established security protocols.

**Scientific Justification:**

Fibromyalgia is a chronic condition characterized by widespread pain, fatigue, and alterations in body perception, which together severely limit functionality and quality of life. Current scientific evidence supports the use of therapeutic exercise as an effective strategy to ameliorate the symptoms of fibromyalgia. However, adherence to these exercise programmes is often compromised by pain perception, fear of movement (kinesiophobia), and altered body representation.

This study seeks to address these limitations by integrating immersive virtual reality (IVR) with a therapeutic exercise programme. IVR can modify the visual and proprioceptive perception of movement, potentially leading to increased exercise tolerance, reduced threat perception, and decreased pain impact. Additionally, the intervention is expected to help modulate the body schema, thereby promoting better sensorimotor integration and enhancing functionality.

Despite the growing interest in the use of virtual reality for chronic pain management, evidence regarding its effectiveness in fibromyalgia patients remains limited. This study aims to contribute robust data on its applicability in this population by examining not only its effects on pain reduction and functional improvement but also its role in modulating body perception and overall well-being.

**Ethical Justification**

This study complies with the principles established in the Declaration of Helsinki (2013) and adheres to Good Clinical Practice guidelines, ensuring the respect and protection of participants in every phase of the research.

The proposed intervention does not present significant risks for the participants, as both IVR and therapeutic exercise have proven to be safe for those with fibromyalgia. Although transient effects such as mild dizziness or discomfort during IVR exposure may occur, these will be monitored closely to ensure participant safety.

All procedures for data collection and handling will be conducted in accordance with the RGPD and LOPD-GDD, ensuring the confidentiality and anonymization of participant information. Participants will be provided with detailed information regarding the study and will sign an informed consent form prior to enrollment. They will also be assured of their right to withdraw from the study at any point without any adverse consequences.

This approach ensures that the research not only advances scientific knowledge regarding the effectiveness of virtual reality for fibromyalgia management but also rigorously protects the rights, well-being, and autonomy of all participants.

**7. Bibliografía**

1. Clauw DJ. Fibromyalgia. JAMA. 2014 Apr 16;311(15):1547.

2. Sandikci SC, Ozbalkan Z. Fatigue in rheumatic diseases. Eur J Rheumatol. 2015 Aug 27;2(3):109–13.

3. Bennett RM, Jones J, Turk DC, Russell IJ, Matallana L. An internet survey of 2,596 people with fibromyalgia. BMC Musculoskelet Disord. 2007 Dec 9;8(1):27.

4. Kleinman L, Mannix S, Arnold LM, Burbridge C, Howard K, McQuarrie K, et al. Assessment of sleep in patients with fibromyalgia: qualitative development of the fibromyalgia sleep diary. Health Qual Life Outcomes. 2014 Dec 14;12(1):111.

5. WALITT B, FITZCHARLES MA, HASSETT AL, KATZ RS, HÄUSER W, WOLFE F. The Longitudinal Outcome of Fibromyalgia: A Study of 1555 Patients. J Rheumatol. 2011 Oct;38(10):2238–46.

6. Ifergane G, Buskila D, Simiseshvely N, Zeev K, Cohen H. Prevalence of Fibromyalgia Syndrome in Migraine Patients. Cephalalgia. 2006 Apr 1;26(4):451–6.

7. Mathieu N. Comorbidités somatiques dans le Syndrome de l’Intestin Irritable : fibromyalgie, syndrome de fatigue chronique et cystite interstitielle/syndrome de la vessie douloureuse. Gastroenterol Clin Biol. 2009 Feb;33:S17–25.

8. Nickel JC, Tripp DA, Pontari M, Moldwin R, Mayer R, Carr LK, et al. Interstitial Cystitis/Painful Bladder Syndrome and Associated Medical Conditions With an Emphasis on Irritable Bowel Syndrome, Fibromyalgia and Chronic Fatigue Syndrome. Journal of Urology. 2010 Oct;184(4):1358–63.

9. Kalichman L. Association between fibromyalgia and sexual dysfunction in women. Clin Rheumatol. 2009 Apr 23;28(4):365–9.

10. Solano C, Martinez A, Becerril L, Vargas A, Figueroa J, Navarro C, et al. Autonomic Dysfunction in Fibromyalgia Assessed by the Composite Autonomic Symptoms Scale (COMPASS). JCR: Journal of Clinical Rheumatology. 2009 Jun;15(4):172–6.

11. Vincent A, McAllister SJ, Singer W, Toussaint LL, Sletten DM, Whipple MO, et al. A Report of the Autonomic Symptom Profile in Patients With Fibromyalgia. JCR Journal of Clinical Rheumatology. 2014 Mar;20(2):106–8.

12. Wolfe F, Smythe HA, Yunus MB, Bennett RM, Bombardier C, Goldenberg DL, et al. The american college of rheumatology 1990 criteria for the classification of fibromyalgia. Arthritis Rheum. 1990 Feb 9;33(2):160–72.

13. Yunus MB, Aldag JC. Restless legs syndrome and leg cramps in fibromyalgia syndrome: a controlled study. BMJ. 1996 May 25;312(7042):1339–1339.

14. Viola-Saltzman M, Watson NF, Bogart A, Goldberg ; Jack, Buchwald D, Watson SM;, et al. High Prevalence of Restless Legs Syndrome among Patients with Fibromyalgia: A Controlled Cross-Sectional Study. Vol. 6, Journal of Clinical Sleep Medicine. 2010.

15. Stehlik R, Arvidsson L, Ulfberg J. Restless Legs Syndrome Is Common among Female Patients with Fibromyalgia. Eur Neurol. 2009;61(2):107–11.

16. Jones KD, Horak FB, Winters-Stone K, Irvine JM, Bennett RM. Fibromyalgia Is Associated With Impaired Balance and Falls. JCR: Journal of Clinical Rheumatology. 2009 Jan;15(1):16–21.

17. Kessler RC, Berglund P, Demler O, Jin R, Koretz D, Merikangas KR, et al. The Epidemiology of Major Depressive Disorder. JAMA. 2003 Jun 18;289(23):3095.

18. González E, Elorza J, Failde I. Fibromyalgia and psychiatric comorbidity: their effect on the quality of life patients. Actas Esp Psiquiatr. 2010;38(5):295–300.

19. Galvez-Sánchez CM, Duschek S, Reyes del Paso GA. <p>Psychological impact of fibromyalgia: current perspectives</p>. Psychol Res Behav Manag. 2019 Feb;Volume 12:117–27.

20. Branco JC, Bannwarth B, Failde I, Abello Carbonell J, Blotman F, Spaeth M, et al. Prevalence of Fibromyalgia: A Survey in Five European Countries. Semin Arthritis Rheum. 2010 Jun;39(6):448–53.

21. White KP, Speechley M, Harth M, Ostbye T. The London Fibromyalgia Epidemiology Study: the prevalence of fibromyalgia syndrome in London, Ontario. J Rheumatol. 1999 Jul;26(7):1570–6.

22. Spaeth M. Epidemiology, costs, and the economic burden of fibromyalgia. Arthritis Res Ther. 2009;11(3):117.

23. Häuser W, Sarzi-Puttini P, Fitzcharles MA. Fibromyalgia syndrome: under-, over- and misdiagnosis. Clin Exp Rheumatol. 2019;37 Suppl 116(1):90–7.

24. Lachaine J, Beauchemin C, Landry PA. Clinical and Economic Characteristics of Patients With Fibromyalgia Syndrome. Clin J Pain. 2010 May;26(4):284–90.

25. Berger A, Dukes E, Martin S, Edelsberg J, Oster G. Characteristics and healthcare costs of patients with fibromyalgia syndrome. Int J Clin Pract. 2007 Jul 26;61(9):1498–508.

26. Knight T, Schaefer C, Chandran, Zlateva G, Winkelmann, Perrot. Health-resource use and costs associated with fibromyalgia in France, Germany, and the United States. ClinicoEconomics and Outcomes Research. 2013 Apr;171.

27. Lacasse A, Bourgault P, Choinière M. Fibromyalgia-related costs and loss of productivity: a substantial societal burden. BMC Musculoskelet Disord. 2016 Dec 16;17(1):168.

28. Guymer EK, Littlejohn GO, Brand CK, Kwiatek RA. Fibromyalgia onset has a high impact on work ability in Australians. Intern Med J. 2016 Sep 16;46(9):1069–74.

29. Taylor SJ, Steer M, Ashe SC, Furness PJ, Haywood-Small S, Lawson K. Patients’ perspective of the effectiveness and acceptability of pharmacological and non-pharmacological treatments of fibromyalgia. Scand J Pain. 2019 Jan 28;19(1):167–81.

30. Taylor SJ, Steer M, Ashe SC, Furness PJ, Haywood-Small S, Lawson K. Patients’ perspective of the effectiveness and acceptability of pharmacological and non-pharmacological treatments of fibromyalgia. Scand J Pain. 2019 Jan 1;19(1):167–81.

31. Kayo AH, Peccin MS, Sanches CM, Trevisani VFM. Effectiveness of physical activity in reducing pain in patients with fibromyalgia: a blinded randomized clinical trial. Rheumatol Int. 2012 Aug 19;32(8):2285–92.

32. Bircan Ç, Karasel SA, Akgün B, El Ö, Alper S. Effects of muscle strengthening versus aerobic exercise program in fibromyalgia. Rheumatol Int. 2008 Apr 3;28(6):527–32.

33. Assumpção A, Matsutani LA, Yuan SL, Santo AS, Sauer J, Mango P, et al. Muscle stretching exercises and resistance training in fibromyalgia: which is better? A three-arm randomized controlled trial. Eur J Phys Rehabil Med. 2018 Sep;54(5).

34. Larsson A, Palstam A, Löfgren M, Ernberg M, Bjersing J, Bileviciute-Ljungar I, et al. Resistance exercise improves muscle strength, health status and pain intensity in fibromyalgia—a randomized controlled trial. Arthritis Res Ther. 2015 Jun 18;17(1):161.

35. Gavi MBRO, Vassalo DV, Amaral FT, Macedo DCF, Gava PL, Dantas EM, et al. Strengthening Exercises Improve Symptoms and Quality of Life but Do Not Change Autonomic Modulation in Fibromyalgia: A Randomized Clinical Trial. PLoS One. 2014 Mar 20;9(3):e90767.

36. HÃ¤kkinen A, HÃ¤kkinen K, Hannonen P, Alen M. Strength training induced adaptations in neuromuscular function of premenopausal women with fibromyalgia: comparison with healthy women. Ann Rheum Dis. 2001 Jan;60(1):21–6.

37. Sañudo B, Galiano D, Carrasco L, Blagojevic M, de Hoyo M, Saxton J. Aerobic Exercise Versus Combined Exercise Therapy in Women With Fibromyalgia Syndrome: A Randomized Controlled Trial. Arch Phys Med Rehabil. 2010 Dec;91(12):1838–43.

38. Gowans SE, deHueck A, Voss S, Silaj A, Abbey SE, Reynolds WJ. Effect of a randomized, controlled trial of exercise on mood and physical function in individuals with fibromyalgia. Arthritis Rheum. 2001 Dec;45(6):519–29.

39. Wang C, Schmid CH, Fielding RA, Harvey WF, Reid KF, Price LL, et al. Effect of tai chi versus aerobic exercise for fibromyalgia: comparative effectiveness randomized controlled trial. BMJ. 2018 Mar 21;k851.

40. Gupta A, Scott K, Dukewich M. Innovative Technology Using Virtual Reality in the Treatment of Pain: Does It Reduce Pain via Distraction, or Is There More to It? Pain Medicine. 2018 Jan 1;19(1):151–9.

41. Li L, Yu F, Shi D, Shi J, Tian Z, Yang J, et al. Application of virtual reality technology in clinical medicine [Internet]. Vol. 9, Am J Transl Res. 2017. Available from: www.ajtr.org/ISSN:1943-8141/AJTR0055713

42. Lee SH, Jung H, Yun SJ, Oh B, Seo HG. Upper Extremity Rehabilitation Using Fully Immersive Virtual Reality Games With a Head Mount Display: A Feasibility Study. PM&R. 2020 Mar 3;12(3):257–62.

43. Carvalho MS de, Carvalho LC, Menezes F da S, Frazin A, Gomes E da C, Iunes DH. Effects of Exergames in Women with Fibromyalgia: A Randomized Controlled Study. Games Health J. 2020 Oct 1;9(5):358–67.

44. Collado-Mateo D, Dominguez-Muñoz FJ, Adsuar JC, Garcia-Gordillo MA, Gusi N. Effects of Exergames on Quality of Life, Pain, and Disease Effect in Women With Fibromyalgia: A Randomized Controlled Trial. Arch Phys Med Rehabil. 2017 Sep;98(9):1725–31.

45. Villafaina S, Collado-Mateo D, Fuentes JP, Rohlfs-Domínguez P, Gusi N. Effects of Exergames on Brain Dynamics in Women with Fibromyalgia: A Randomized Controlled Trial. J Clin Med. 2019 Jul 11;8(7):1015.

46. Collado-Mateo D, Dominguez-Muñoz FJ, Adsuar JC, Merellano-Navarro E, Gusi N. Exergames for women with fibromyalgia: a randomised controlled trial to evaluate the effects on mobility skills, balance and fear of falling. PeerJ. 2017 Apr 20;5:e3211.

47. Garcia-Palacios A, Herrero R, Vizcaíno Y, Belmonte MA, Castilla D, Molinari G, et al. Integrating Virtual Reality With Activity Management for the Treatment of Fibromyalgia. Clin J Pain. 2015 Jun;31(6):564–72.

48. Leon-Llamas JL, Villafaina S, Murillo-Garcia A, Dominguez-Muñoz FJ, Gusi N. Effects of 24-Week Exergame Intervention on the Gray Matter Volume of Different Brain Structures in Women with Fibromyalgia: A Single-Blind, Randomized Controlled Trial. J Clin Med. 2020 Jul 30;9(8):2436.

49. Martín‐Martínez JP, Villafaina S, Collado‐Mateo D, Pérez‐Gómez J, Gusi N. Effects of 24‐week exergame intervention on physical function under single‐ and dual‐task conditions in fibromyalgia: A randomized controlled trial. Scand J Med Sci Sports. 2019 Oct 3;29(10):1610–7.

50. Polat M, Kahveci A, Muci B, Günendi Z, Kaymak Karataş G. The Effect of Virtual Reality Exercises on Pain, Functionality, Cardiopulmonary Capacity, and Quality of Life in Fibromyalgia Syndrome: A Randomized Controlled Study. Games Health J. 2021 Jun 1;10(3):165–73.

51. Villafaina S, Collado-Mateo D, Domínguez-Muñoz FJ, Fuentes-García JP, Gusi N. Benefits of 24-Week Exergame Intervention on Health-Related Quality of Life and Pain in Women with Fibromyalgia: A Single-Blind, Randomized Controlled Trial. Games Health J. 2019 Dec 1;8(6):380–6.

52. Villafaina S, Borrega-Mouquinho Y, Fuentes-García JP, Collado-Mateo D, Gusi N. Effect of Exergame Training and Detraining on Lower-Body Strength, Agility, and Cardiorespiratory Fitness in Women with Fibromyalgia: Single-Blinded Randomized Controlled Trial. Int J Environ Res Public Health. 2019 Dec 24;17(1):161.

53. Cortés-Pérez I, Zagalaz-Anula N, Ibancos-Losada M del R, Nieto-Escámez FA, Obrero-Gaitán E, Osuna-Pérez MC. Virtual Reality-Based Therapy Reduces the Disabling Impact of Fibromyalgia Syndrome in Women: Systematic Review with Meta-Analysis of Randomized Controlled Trials. J Pers Med. 2021 Nov 9;11(11):1167.

54. Rose T, Nam CS, Chen KB. Immersion of virtual reality for rehabilitation - Review. Appl Ergon. 2018 May;69:153–61.

55. Kozhevnikov M, Kosslyn S, Shephard J. Spatial versus object visualizers: A new characterization of visual cognitive style. Mem Cognit. 2005 Jun;33(4):710–26.

56. Shahrbanian S, Ma X, Aghaei N, Korner-Bitensky N, Moshiri K, Simmonds MJ. Use of virtual reality (immersive vs. non immersive) for pain management in children and adults: A systematic review of evidence from randomized controlled trials [Internet]. Vol. 2012, Pelagia Research Library European Journal of Experimental Biology. Available from: www.pelagiaresearchlibrary.com

57. Darnall BD, Krishnamurthy P, Tsuei J, Minor JD. Self-Administered Skills-Based Virtual Reality Intervention for Chronic Pain: Randomized Controlled Pilot Study. JMIR Form Res. 2020 Jul 7;4(7):e17293.

58. Gulsen C, Soke F, Eldemir K, Apaydin Y, Ozkul C, Guclu-Gunduz A, et al. Effect of fully immersive virtual reality treatment combined with exercise in fibromyalgia patients: a randomized controlled trial. Assistive Technology. 2022 May 4;34(3):256–63.

59. Venuturupalli RS, Chu T, Vicari M, Kumar A, Fortune N, Spielberg B. Virtual Reality–Based Biofeedback and Guided Meditation in Rheumatology: A Pilot Study. ACR Open Rheumatol. 2019 Dec 11;1(10):667–75.

60. Gulsen C, Soke F, Eldemir K, Apaydin Y, Ozkul C, Guclu-Gunduz A, et al. Effect of fully immersive virtual reality treatment combined with exercise in fibromyalgia patients: a randomized controlled trial. Assistive Technology. 2020;1–8.

61. Monterde S, Salvat I, Montull S, Fernández-Ballart J. Validación de la versión española del Fibromyalgia Impact Questionnaire. Revista Española de Reumatología [Internet]. 2004 Nov 1 [cited 2025 Jan 29];31(9):507–13. Available from: http://www.elsevier.es/es-revista-revista-espanola-reumatologia-29-articulo-validacion-version-espanola-del-fibromyalgia-13068512

62. Salgueiro M, García-Leiva JM, Ballesteros J, Hidalgo J, Molina R, Calandre EP. Validation of a Spanish version of the Revised Fibromyalgia Impact Questionnaire (FIQR). Health Qual Life Outcomes. 2013 Dec 1;11(1):132.

63. van Hout B, Janssen MF, Feng YS, Kohlmann T, Busschbach J, Golicki D, et al. Interim Scoring for the EQ-5D-5L: Mapping the EQ-5D-5L to EQ-5D-3L Value Sets. Value in Health. 2012 Jul;15(5):708–15.

64. Herdman M, Gudex C, Lloyd A, Janssen MF, Kind P, Parkin D, et al. Development and preliminary testing of the new five-level version of EQ-5D (EQ-5D-5L). Quality of Life Research. 2011 Dec 9;20(10):1727–36.

65. Hernandez G, Garin O, Pardo Y, Vilagut G, Pont À, Suárez M, et al. Validity of the EQ–5D–5L and reference norms for the Spanish population. Quality of Life Research. 2018 Sep 16;27(9):2337–48.

66. Mendoza-Muñoz M, Morenas-Martín J, Rodal M, García-Matador J, García-Gordillo MÁ, Calzada-Rodríguez JI. Knowledge about Fibromyalgia in Fibromyalgia Patients and Its Relation to HRQoL and Physical Activity. Biology (Basel). 2021 Jul 16;10(7):673.

67. Vallejo MA, Rivera J, Esteve-Vives J, Rodríguez-Muñoz MF. Uso del cuestionario Hospital Anxiety and Depression Scale (HADS) para evaluar la ansiedad y la depresión en pacientes con fibromialgia. Rev Psiquiatr Salud Ment. 2012 Apr;5(2):107–14.

68. Herrero MJ, Blanch J, Peri JM, De Pablo J, Pintor L, Bulbena A. A validation study of the hospital anxiety and depression scale (HADS) in a Spanish population. Gen Hosp Psychiatry. 2003 Jul;25(4):277–83.

69. Munguía-Izquierdo D, Segura-Jiménez V, Camiletti-Moirón D, Pulido-Martos M, Alvarez-Gallardo IC, Romero A, et al. Multidimensional Fatigue Inventory: Spanish adaptation and psychometric properties for fibromyalgia patients. The Al-Andalus study. Clin Exp Rheumatol. 2012;30(6 Suppl 74):94–102.

70. Williams DA, Arnold LM. Measures of fibromyalgia: Fibromyalgia Impact Questionnaire (FIQ), Brief Pain Inventory (BPI), Multidimensional Fatigue Inventory (MFI‐20), Medical Outcomes Study (MOS) Sleep Scale, and Multiple Ability Self‐Report Questionnaire (MASQ). Arthritis Care Res (Hoboken). 2011 Nov 7;63(S11).

71. Hita-Contreras F, Martínez-López E, Latorre-Román PA, Garrido F, Santos MA, Martínez-Amat A. Reliability and validity of the Spanish version of the Pittsburgh Sleep Quality Index (PSQI) in patients with fibromyalgia. Rheumatol Int. 2014 Jul 8;34(7):929–36.

72. Osorio CD, Gallinaro AL, Lorenzi-Filho G, Lage L V. Sleep quality in patients with fibromyalgia using the Pittsburgh Sleep Quality Index. J Rheumatol. 2006 Sep;33(9):1863–5.

73. García Campayo J, Rodero B, Alda M, Sobradiel N, Montero J, Moreno S. Validación de la versión española de la escala de la catastrofización ante el dolor (Pain Catastrophizing Scale) en la fibromialgia. Med Clin (Barc). 2008 Oct;131(13):487–92.

74. Morris LD, Grimmer-Somers KA, Spottiswoode B, Louw QA. Virtual reality exposure therapy as treatment for pain catastrophizing in fibromyalgia patients: proof-of-concept study (Study Protocol). BMC Musculoskelet Disord. 2011 Dec 30;12(1):85.

75. Sullivan MJ. The Pain Catastrophizing Scale User Manual. 1995.

76. Cuesta-Vargas AI, Roldan-Jimenez C, Neblett R, Gatchel RJ. Cross-cultural adaptation and validity of the Spanish central sensitization inventory. Springerplus. 2016 Dec 21;5(1):1837.

77. Weaver KR, Griffioen MA, Klinedinst NJ, Galik E, Duarte AC, Colloca L, et al. Quantitative Sensory Testing Across Chronic Pain Conditions and Use in Special Populations. Frontiers in pain research (Lausanne, Switzerland). 2021;2:779068.

78. Wodehouse T, Poply K, Ramaswamy S, Snidvongs S, Bourke J, Tahir H, et al. A pilot study investigating whether quantitative sensory testing alters after treatment in patients with fibromyalgia. Br J Pain. 2018 Nov 15;12(4):250–6.

79. Gómez-Pérez L, López-Martínez AE, Ruiz-Párraga GT. Psychometric Properties of the Spanish Version of the Tampa Scale for Kinesiophobia (TSK). J Pain. 2011 Apr;12(4):425–35.

80. Roelofs J, Goubert L, Peters ML, Vlaeyen JWS, Crombez G. The Tampa Scale for Kinesiophobia: further examination of psychometric properties in patients with chronic low back pain and fibromyalgia. European Journal of Pain. 2004 Oct 11;8(5):495–502.

81. Martín-Martínez JP, Collado-Mateo D, Domínguez-Muñoz FJ, Villafaina S, Gusi N, Pérez-Gómez J. Reliability of the 30 s Chair Stand Test in Women with Fibromyalgia. Int J Environ Res Public Health. 2019 Jul 2;16(13):2344.

82. Carbonell-Baeza A, Álvarez-Gallardo I, Segura-Jiménez V, Castro-Piñero J, Ruiz J, Delgado-Fernández M, et al. Reliability and Feasibility of Physical Fitness Tests in Female Fibromyalgia Patients. Int J Sports Med. 2014 Oct 20;36(02):157–62.

83. Rikli RE, Jones CJ. Development and Validation of a Functional Fitness Test for Community-Residing Older Adults. J Aging Phys Act. 1999 Apr;7(2):129–61.

84. Podsiadlo D, Richardson S. The Timed “Up &amp; Go”: A Test of Basic Functional Mobility for Frail Elderly Persons. J Am Geriatr Soc. 1991 Feb 27;39(2):142–8.

85. Collado-Mateo D, Domínguez-Muñoz FJ, Adsuar JC, Merellano-Navarro E, Olivares PR, Gusi N. Reliability of the Timed Up and Go Test in Fibromyalgia. Rehabilitation Nursing. 2018 Jan;43(1):35–9.

86. Nordenskiöld UM, Grimby G. Grip Force in Patients with Rheumatoid Arthritis and Fibromyalgia and in Healthy Subjects. A Study with the Grippit Instrument. Scand J Rheumatol. 1993 Jan 12;22(1):14–9.

87. 3810.

88. Kovacs FM, Muriel A, Medina JM, Abraira V, Sánchez MDC, Jaúregui JO. Psychometric Characteristics of the Spanish Version of the FAB Questionnaire. Spine (Phila Pa 1976). 2006 Jan;31(1):104–10.

89. García-Dopico N, De La Torre-Luque A, Wand BM, Velasco-Roldán O, Sitges C. The cross-cultural adaptation, validity, and reliability of the Spanish version of the Fremantle Back Awareness Questionnaire. Front Psychol. 2023;14.

90. Świdrak J, Rodriguez T, Polino L, Arias A, Torres X, Sanchez-Vives M V. Drawing the lines of fibromyalgia: a mixed-methods approach to mapping body image, body schema, and emotions in patient subtypes. Psychol Health Med. 2024 Nov 18;1–21.

**7. ANEXOS**

**ANEXO I: Fibromyalgia Impact Questionnaire Revised (FIQR).**

**ANEXO II: EQ-5D-5L**

**ANEXO III: Hospital Anxiety and Depression Scale (HADS).**

**ANEXO IV:** **Multidimensional Fatigue Inventory (MFI-20)**.

**ANEXO V: Pittsburgh Sleep Quality Index (PSQI).**

**ANEXO VI: Pain Catastrophizing Scale (PCS).**

**ANEXO VII: Central Sensitization Inventory (CSI).**

**ANEXO VIII: Tampa Scale for Kinesiophobia (TSK).**

**ANEXO IX: Behavioral Regulation in Exercise Questionnaire-3 (BREQ-3).**

**ANEXO X: Fear-Avoidance Beliefs Questionnaire (FABQ).**

**ANEXO XI: Fremantle Back Awareness Questionnaire (FBAQ).**

**ANEXO I**

**
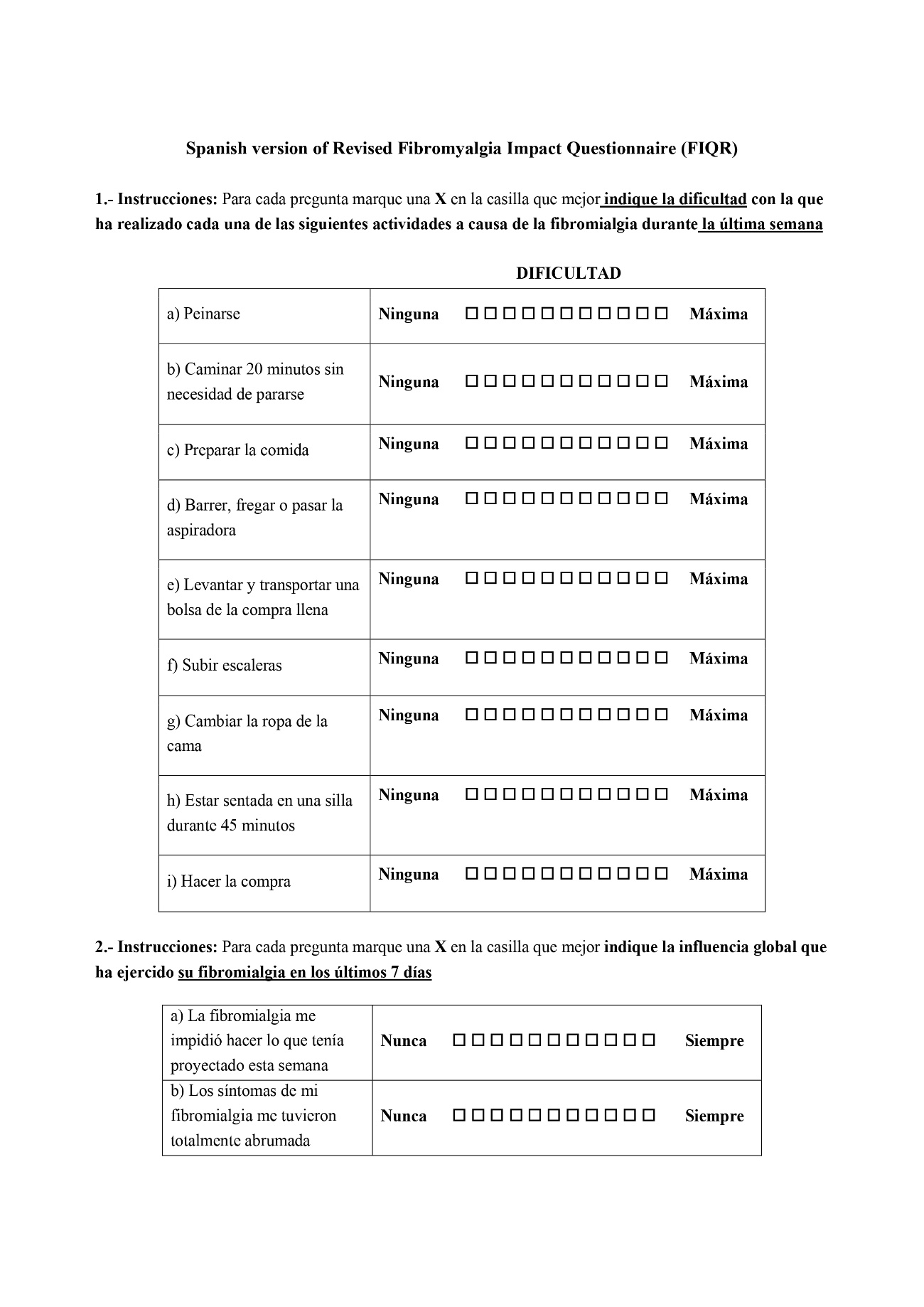
**

**
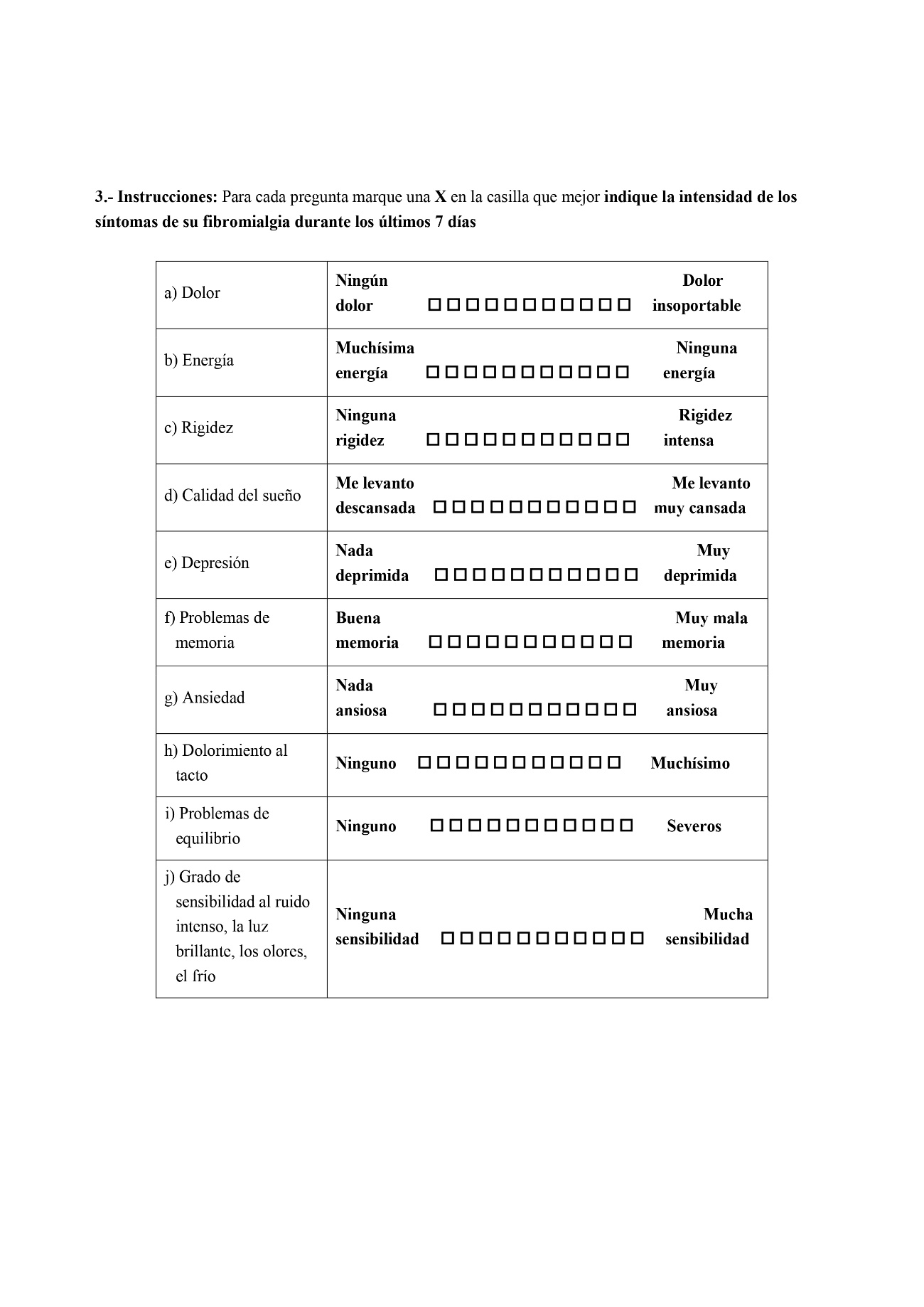
**

**ANEXO II**

**(EQ-5D-5L)**

**
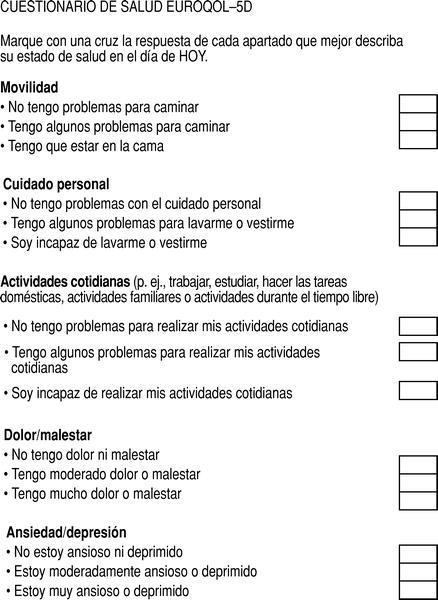
**

**
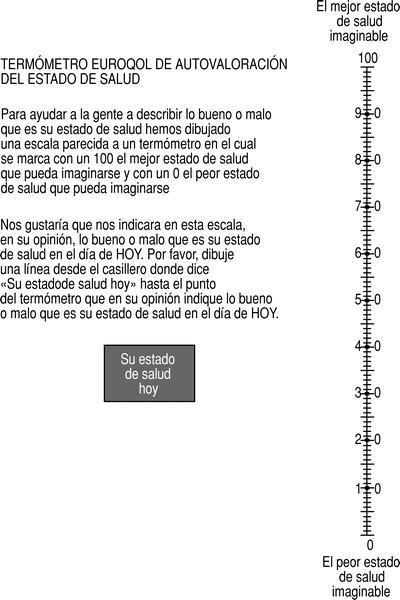
**

**ANEXO III
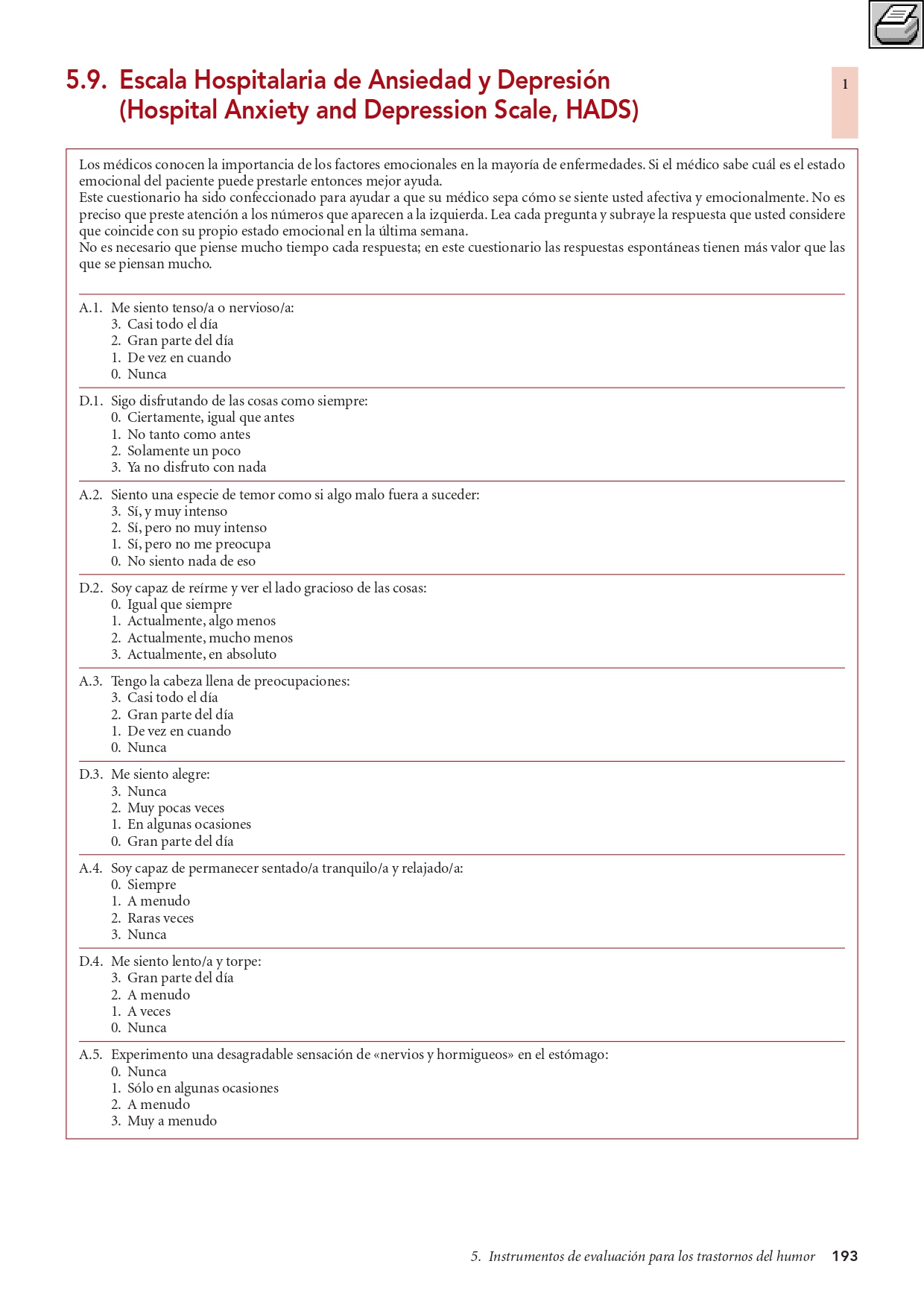
**

**
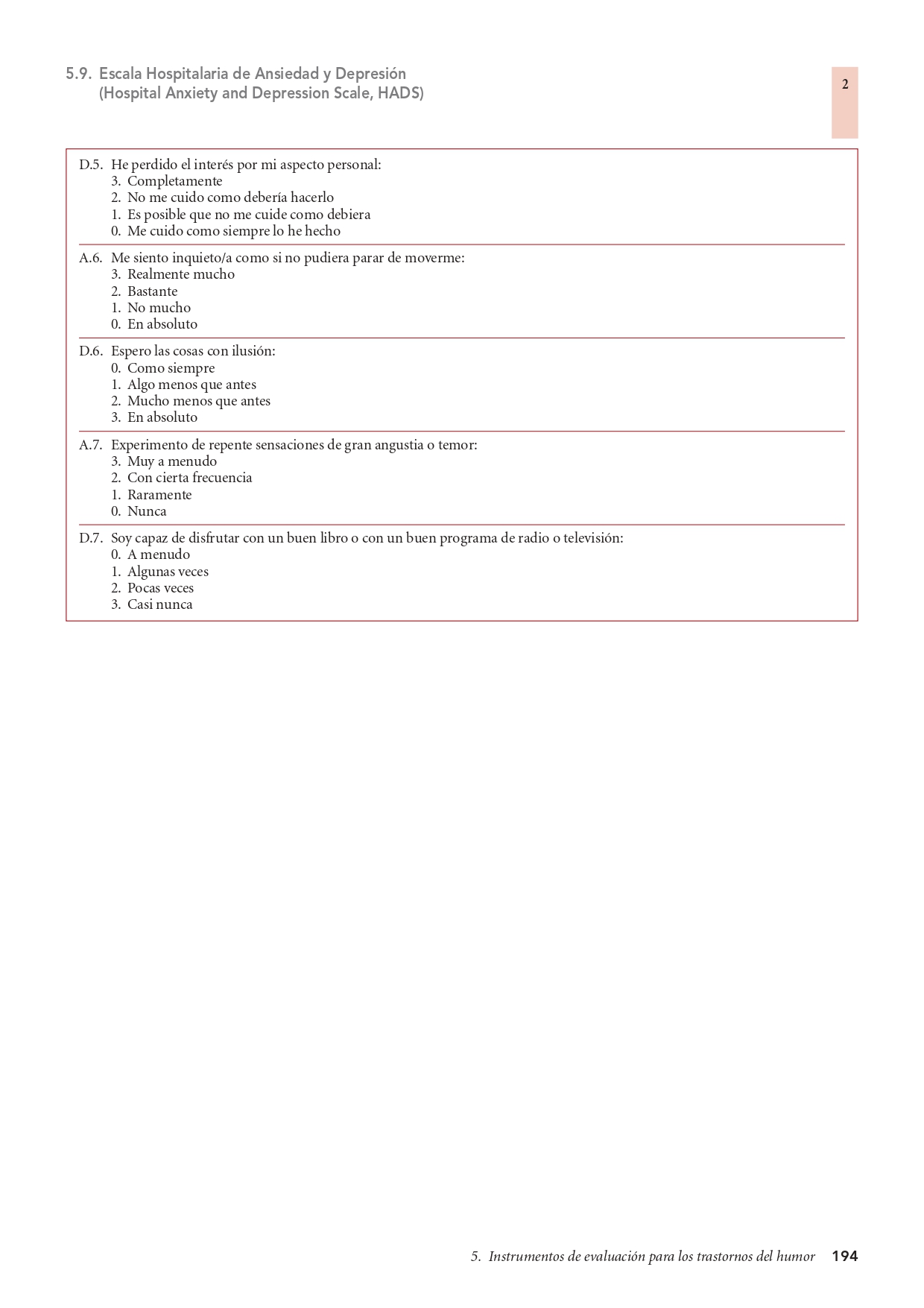
**

**
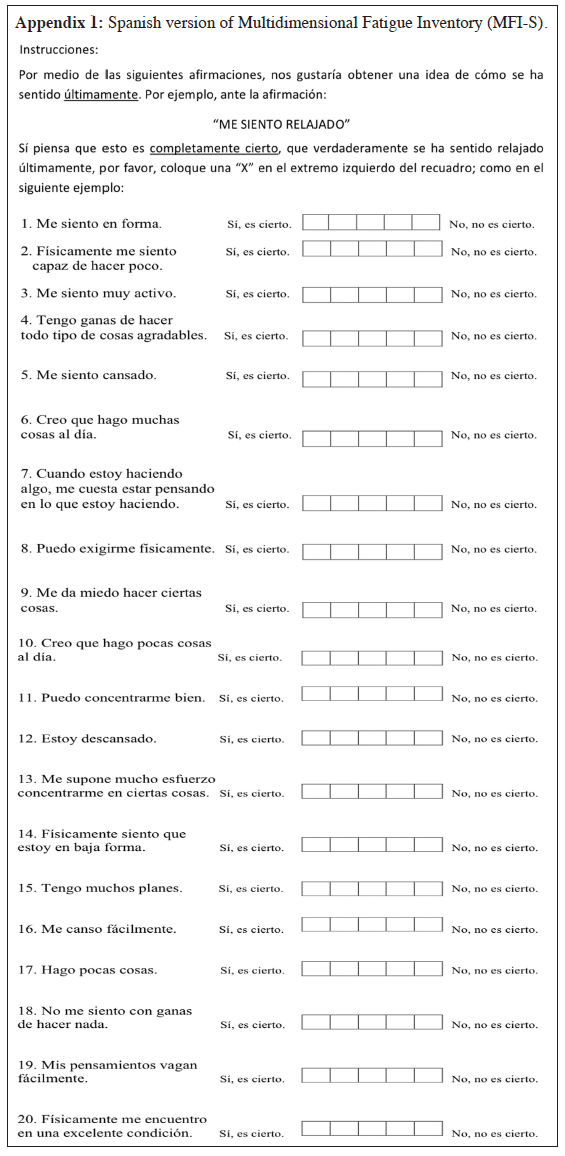
ANEXO IV**

**ANEXO V**

Instrucciones

Las siguientes preguntas se refieren a la forma en que normalmente ha dormido únicamente durante el último mes. Sus respuestas intentarán ajustarse de la manera más exacta a lo ocurrido durante la mayor parte de los días y noches del último mes.

Por favor, conteste a TODAS las preguntas.

1. Durante el último mes: ¿Cuál ha sido, normalmente, su hora de acostarse? _____

2. ¿Cuánto tiempo ha tardado en dormirse, normalmente, las noches, del último mes? _____

3. ¿A qué hora se ha levantado habitualmente por la mañana durante el último mes? _____

4. ¿Cuántas horas calcula que habrá dormido verdaderamente cada noche durante el último mes? (El tiempo puede ser diferente al que usted permanezca en la cama). _____

Para cada una de las siguientes preguntas, elija la repuesta que más se ajuste a su case. Intente contestar a TODAS las preguntas.

5. *Durante el último mes, cuántas veces ha tenido usted problemas para dormir a causa de:*

5a. No poder conciliar el sueño en la primera media hora

_ 0. Ninguna vez en el último mes

_ 1. Menos de una vez a la semana

_ 2. Una o dos veces a la semana

_ 3. Tres o más veces a la semana

5b. Despertarse durante la noche o de madrugada

_ 0. Ninguna vez en el último mes

_ 1. Menos de una vez a la semana

_ 2. Una o dos veces a la semana

_ 3. Tres o más veces a la semana

5c. Tener que levantarse para ir al servicio

_ 0. Ninguna vez en el último mes

_ 1. Menos de una vez a la semana

_ 2. Una o dos veces a la semana

_ 3. Tres o más veces a la semana

5d. No poder respirar bien

_ 0. Ninguna vez en el último mes

_ 1. Menos de una vez a la semana

_ 2. Una o dos veces a la semana

_ 3. Tres o más veces a la semana

5e. Toser o roncar ruidosamente

_ 0. Ninguna vez en el último mes

_ 1. Menos de una vez a la semana

_ 2. Una o dos veces a la semana

_ 3. Tres o más veces a la semana

5f. Sentir frío

_ 0. Ninguna vez en el último mes

_ 1. Menos de una vez a la semana

_ 2. Una o dos veces a la semana

_ 3. Tres o más veces a la semana

5g. Sentir demasiado calor

_ 0. Ninguna vez en el último mes

_ 1. Menos de una vez a la semana

_ 2. Una o dos veces a la semana

_ 3. Tres o más veces a la semana

5h. Tener pesadillas o *malos sueños*

_ 0. Ninguna vez en el último mes

_ 1. Menos de una vez a la semana

_ 2. Una o dos veces a la semana

_ 3. Tres o más veces a la semana

5i. Sufrir dolores

_ 0. Ninguna vez en el último mes

_ 1. Menos de una vez a la semana

_ 2. Una o dos veces a la semana

_ 3. Tres o más veces a la semana

5j. Otras razones. (por favor, descríbalas a continuación): _____

6. Durante el *último mes,* ¿cuántas veces habrá tomado medicinas (por su cuenta o recetadas por al médico) para dormir?

_ 0. Ninguna vez en el último mes

_ 1. Menos de una vez a la semana

_ 2. Una o dos veces a la semana

_ 3. Tres o más veces a la semana

7. Durante el *último mes,* ¿cuántas veces ha sentido somnolencia mientras conducía, comía, o desarrollaba alguna otra actividad?

_ 0. Ninguna vez en el último mes

_ 1. Menos de una vez a la semana

_ 2. Una o dos veces a la semana

_ 3. Tres o más veces a la semana

8. Durante el *último mes,* ¿ha representado para usted mucho problema el *tener ánimos* para realizar alguna de las actividades detalladas en la pregunta anterior?

_0. Ningún problema

_1. Sólo un leve problema

_2. Un problema

_3. Un grave problema

9. Durante el *último mes,* ¿cómo valoraría, en conjunto, la calidad de su sueño?

_ 0. Bastante Buena

_ 1. Buena

_ 2. Mala

_ 3. Bastante mala

**
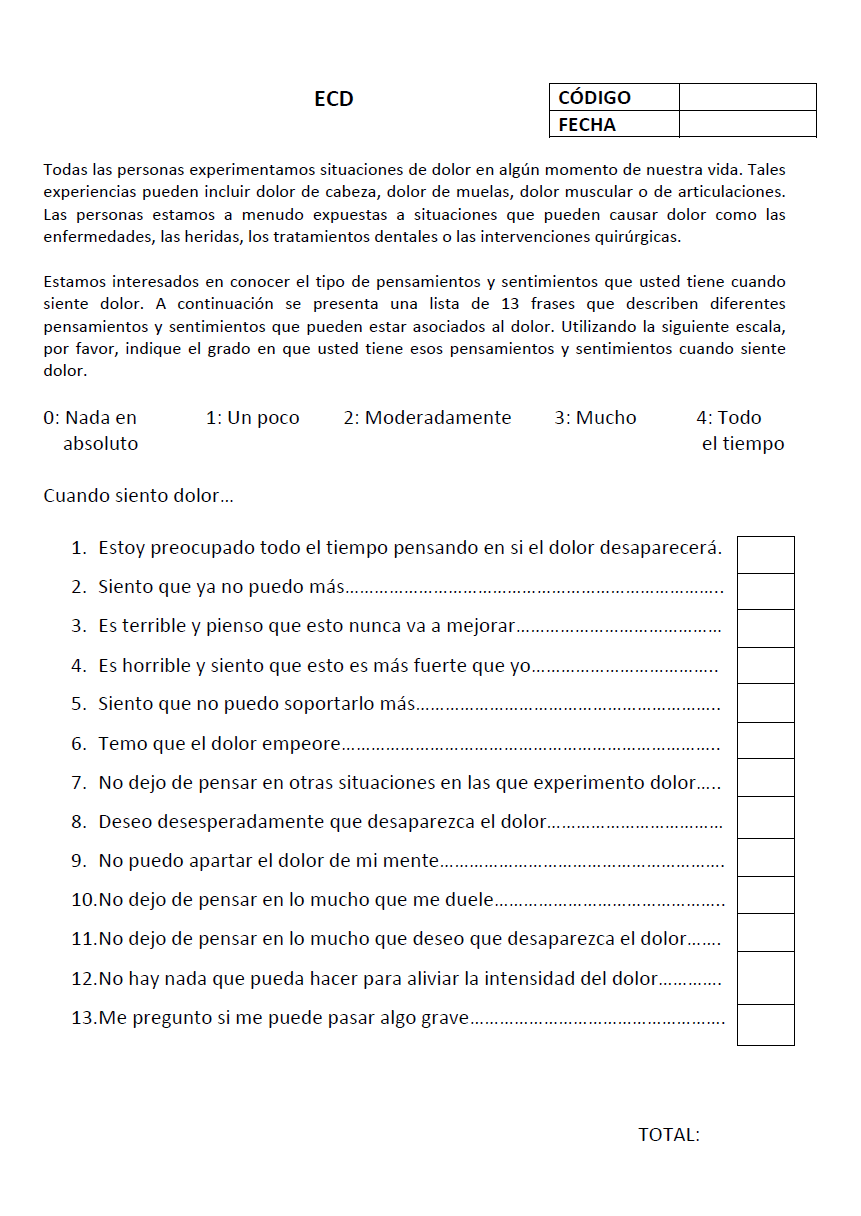
ANEXO VI**

**
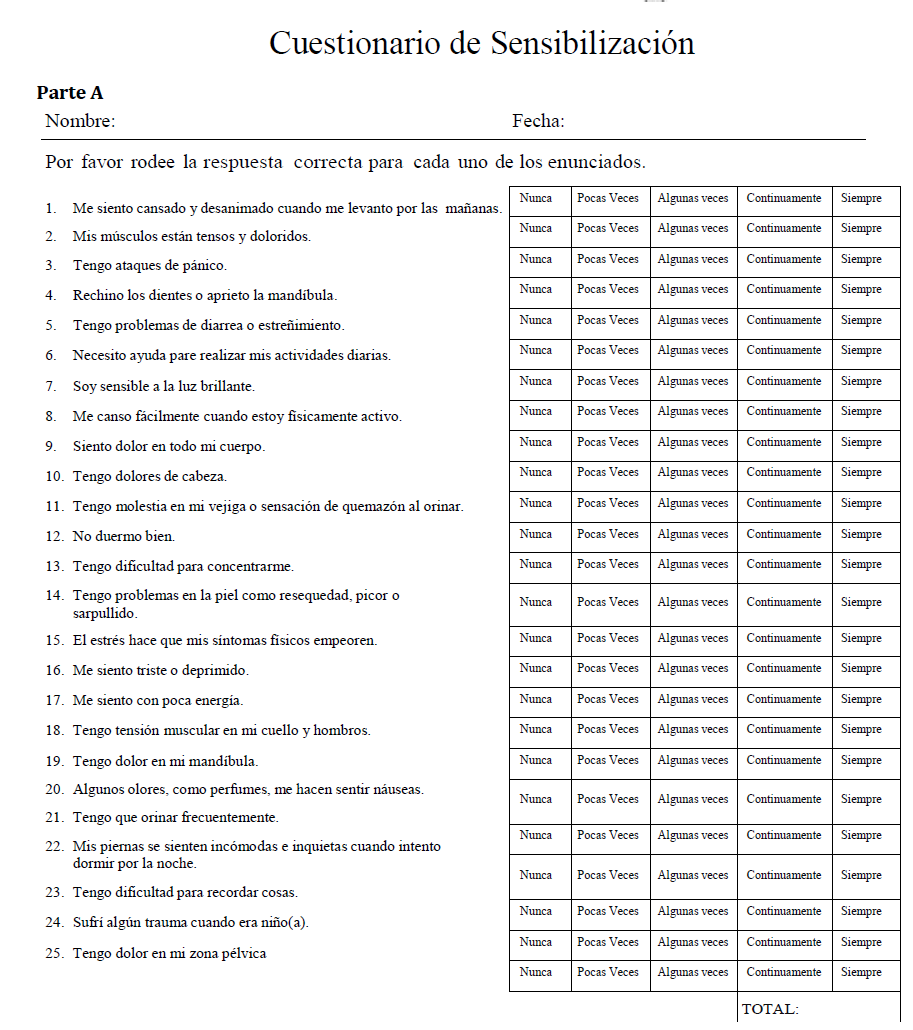
ANEXO VII**

**
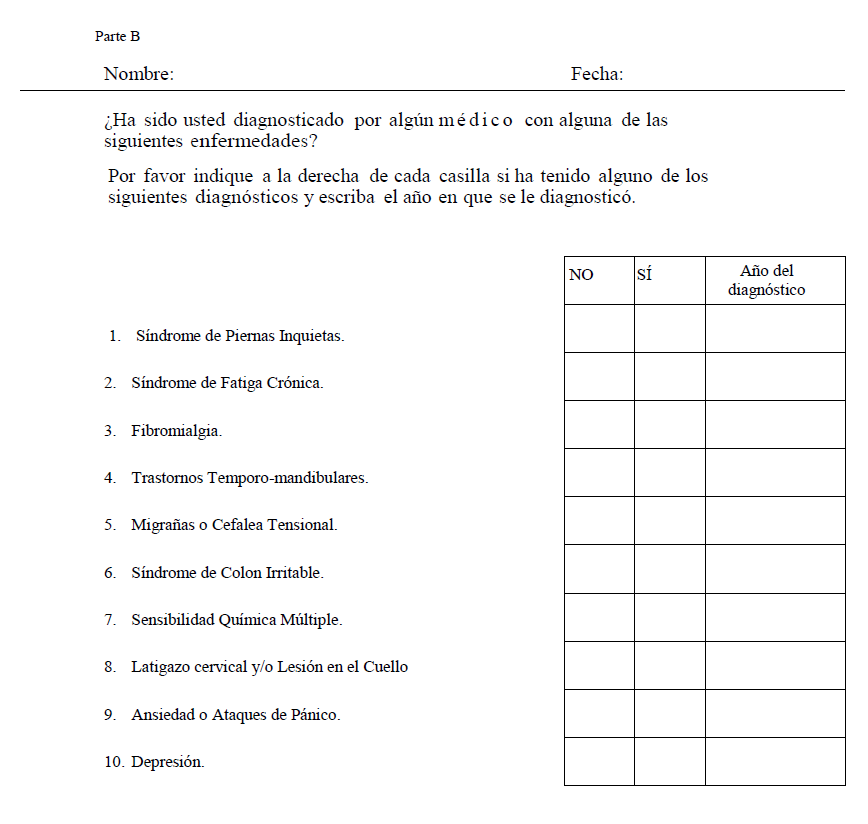
**

**ANEXO VIII**

**
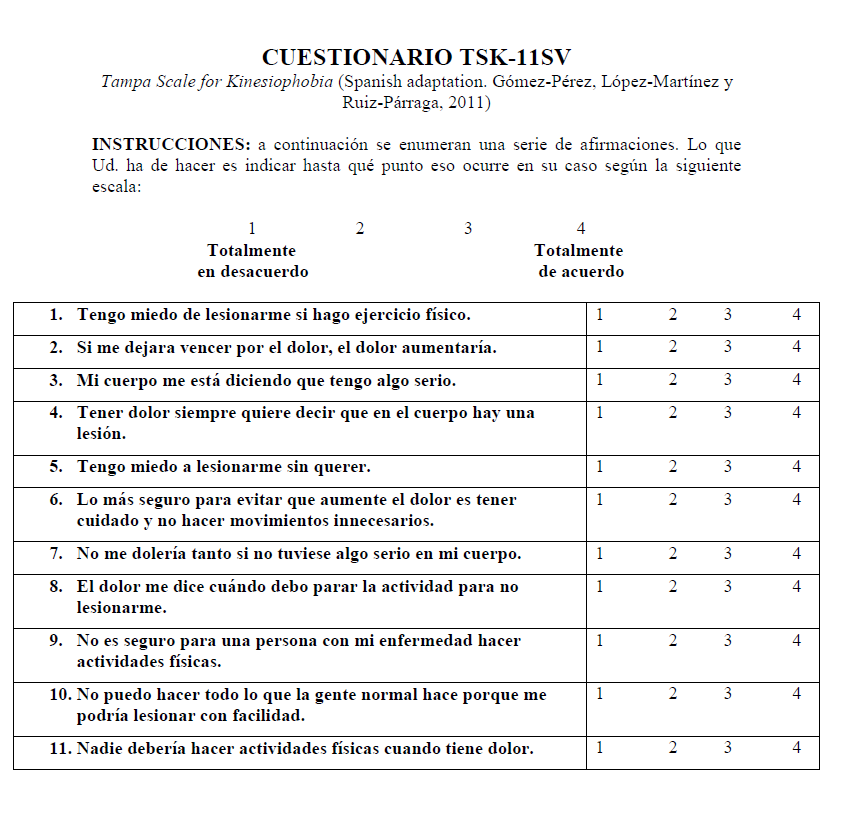
**

**ANEXO IX**

**
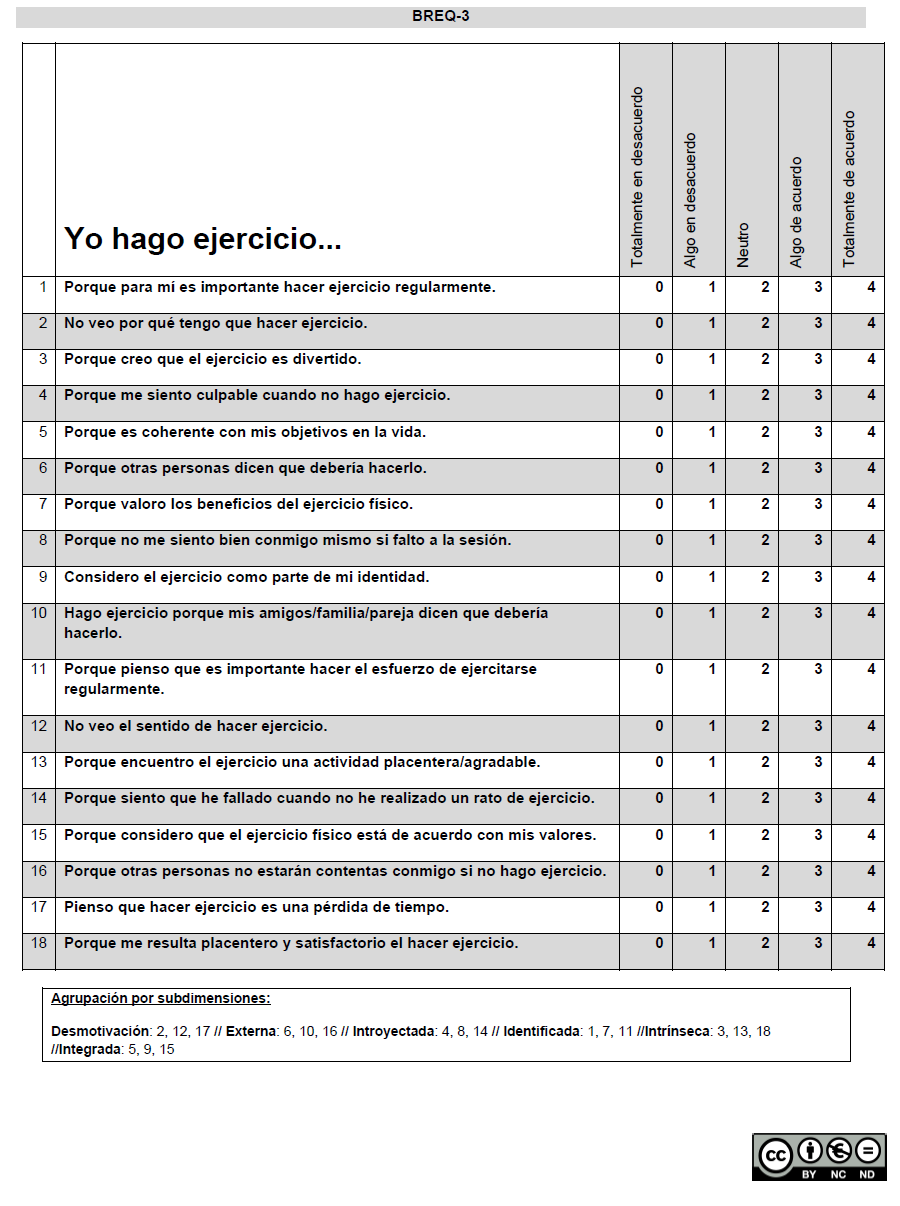
**

**
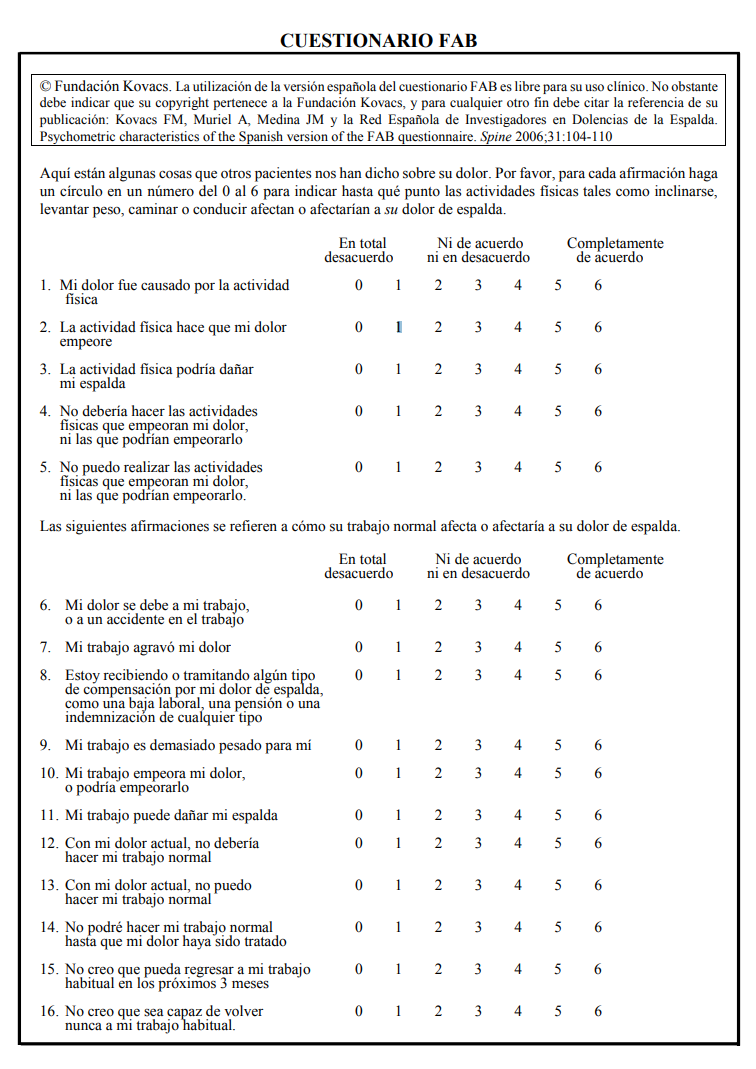
ANEXO X**

**ANEXO X**

**
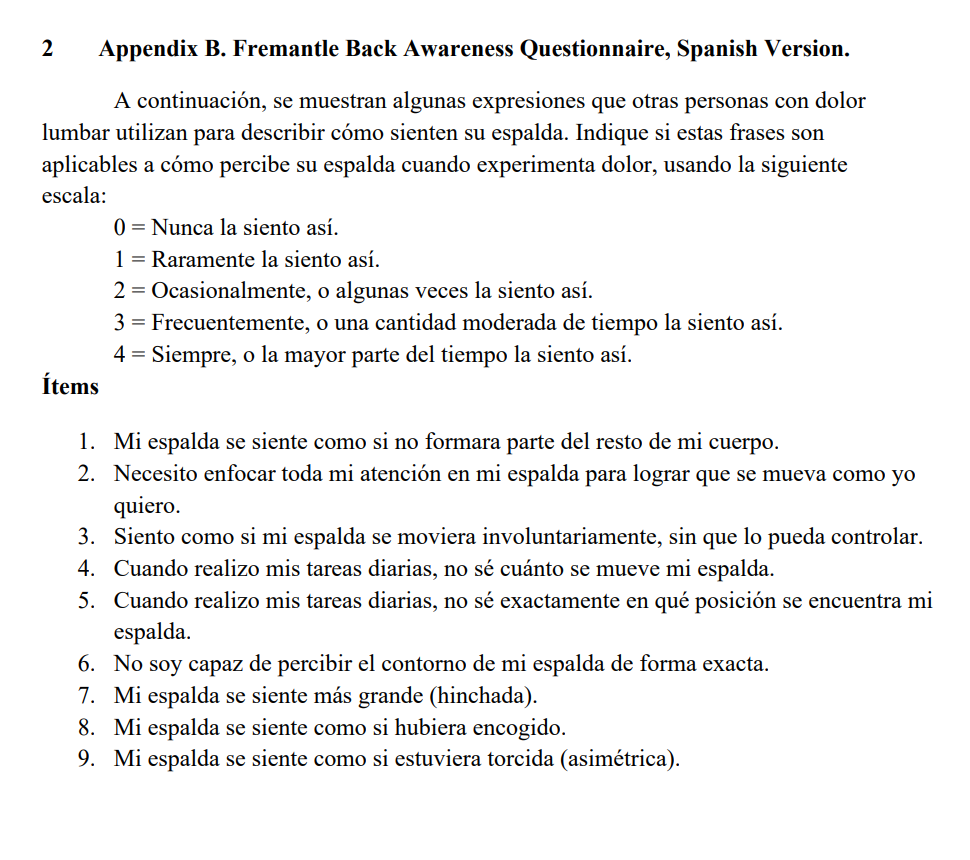
**

**ANEXO XII**

**
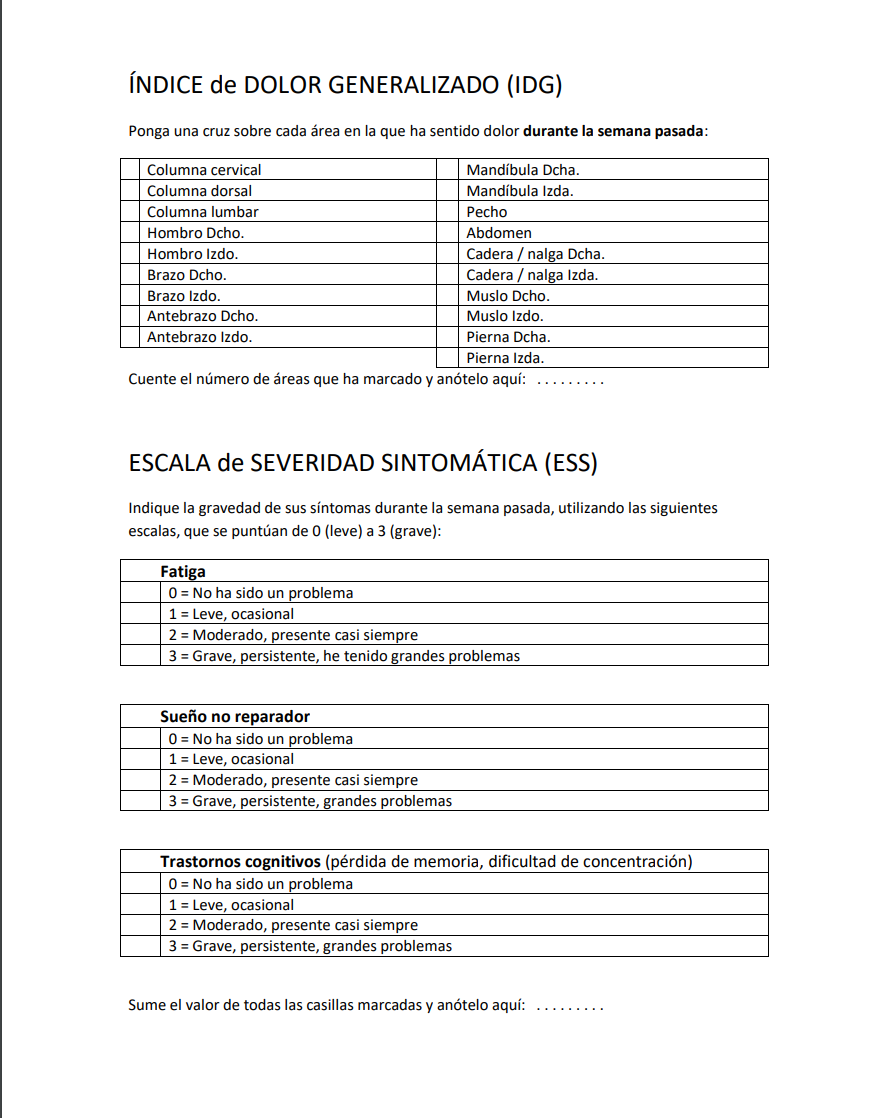
**

**
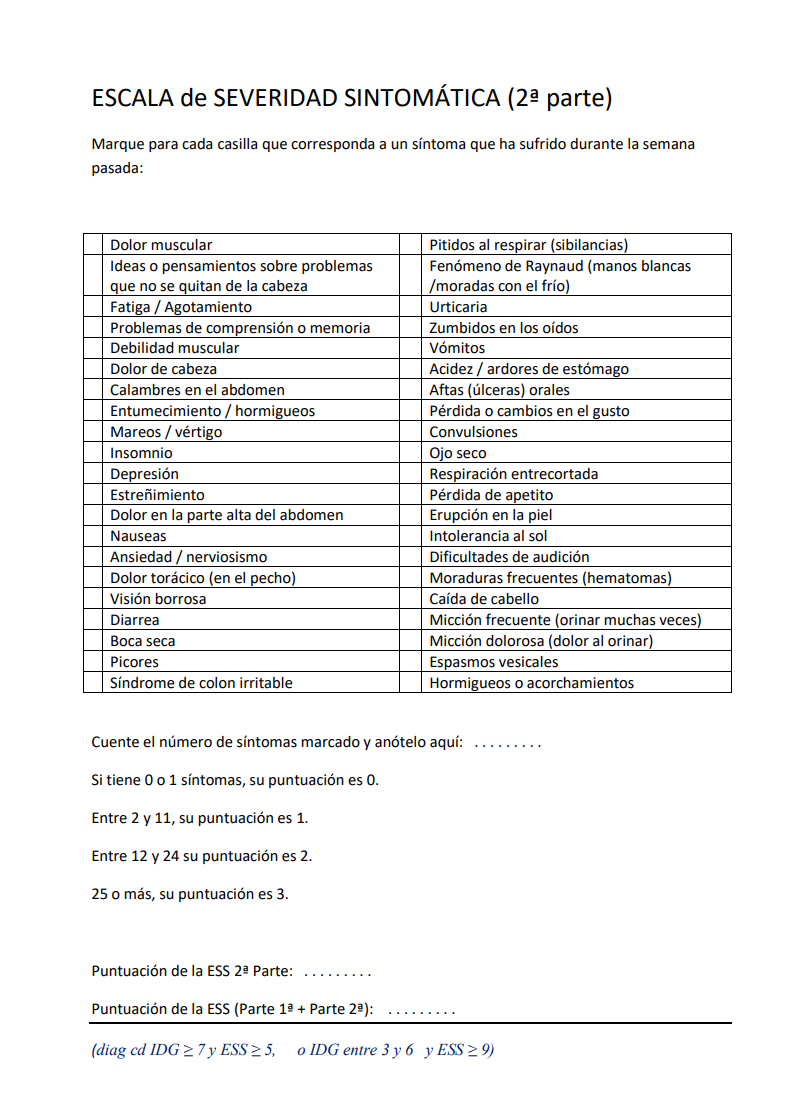
**
