# Supplementary material for: Effectiveness of a fully immersive virtual reality-based therapeutic exercise programme with altered visual feedback in patients with fibromyalgia: A study protocol for a randomised controlled trial
Source: PLoS One. 2026 Jun 4;21(6):e0348346. doi: 10.1371/journal.pone.0348346 (PMC13235889; doi:10.1371/journal.pone.0348346)
Supplement: S3 Appendix — (DOCX) [file pone.0348346.s004.docx]

# **FULLY IMMERSIVE VIRTUAL REALITY INTERVENTION**

## **FULLY IMMERSIVE VIRTUAL REALITY AND VISUAL MANIPULATION SYSTEM**

During the intervention phase, participants allocated in the experimental group will engage with a Fully Immersive Virtual Reality (FIVR) system utilizing the HTC Vive Pro (HTC Corporation), accompanied by motion trackers affixed to the waist, hands, and feet. This configuration will allow for the generation of a gender- and skin tone-matched avatar within the virtual environment, which will be programmed to faithfully mirror the participants’ real-world movements from a first-person perspective, thereby enhancing the sense of embodiment and immersive realism.

To further promote embodiment, participants will observe themselves through a virtual mirror integrated into the environment (Figure 1). Prior to the commencement of the exercises, a calibration procedure will be conducted to align the avatar with the user’s anthropometric parameters, followed by an introductory tutorial designed to acclimate participants to the FIVR setting. Standardized task instructions will be delivered through built-in speakers using pre-recorded audio files to ensure consistency across sessions.

***Figure 1:*** *VR set up and virtual environment.*

For each exercise included in the FIVR program, the system will incorporate subtle visual-proprioceptive feedback alterations intended to manipulate the perceived range of lumbar motion. Two distinct sensorimotor illusions will be implemented to achieve this effect:

## **Lumbar Flexion Illusion:**


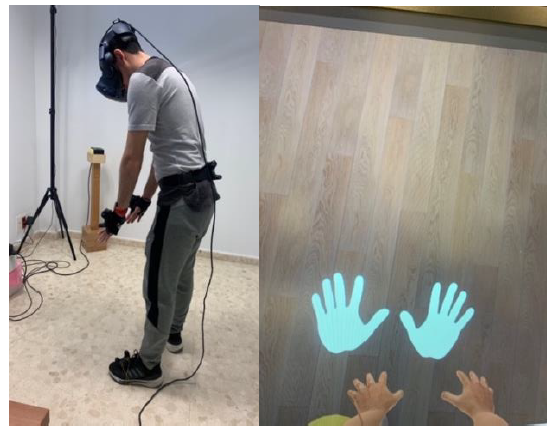
During exercises involving lumbar flexion, the avatar’s arm length will be visually shortened by approximately 20% relative to the participants’ actual limb length (Figure 2). This intentional visual distortion led participants to underestimate the extent of their lumbar flexion, thereby facilitating a greater range of motion during task performance.

**Figure 2**: Lumbar flexion (handprints): the left image shows the view of the researcher and right image shows the view of the participant within the virtual reality space.

## **Lumbar extension illusion:**


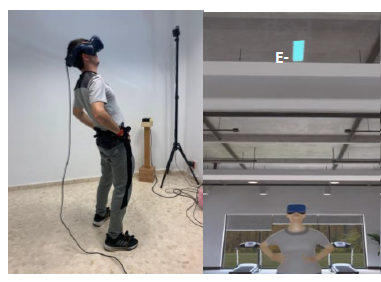
For lumbar extension exercises, a virtual bar will be positioned in front of the participant within the mirrored virtual environment (Figure 3). As participants extend their lumbar spine, the height of the bar will increase in real-time to visually represent movement. However, the system will be calibrated to underestimate the actual range of motion by approximately 10%, subtly inducing the perception that less extension has occurred. This manipulation is anticipated to facilitate a greater range of motion during exercise performance.

**Figure 3: Lumbar extension: the left image shows the view of the researcher and right image shows the view of the participant within the virtual reality space.**

These perceptual manipulations will be applied consistently across twelve intervention sessions, each comprising a structured set of exercises. Through the controlled introduction of these illusions, it is hypothesized that participants will progressively increase their joint range of motion per repetition, while maintaining perceived exertion within tolerable limits. This process is expected to promote motor adaptation and functional improvement.

The strategic use of visual-proprioceptive illusions aims to delay the onset of movement-evoked pain, enhance pain thresholds, reduce kinesiophobia, and improve proprioceptive acuity. These outcomes will be targeted by refining sensorimotor control and spatial awareness of trunk movements. The magnitude and parameters of these illusions will be based on previously validated methodologies used in studies involving individuals with chronic low back pain (cLBP), such as those described by Jaime et al.[1,2] . At S1 video you can view a video in which the procedure for both visual illusions are demonstrated.

## **INTERVENTION DESIGN**


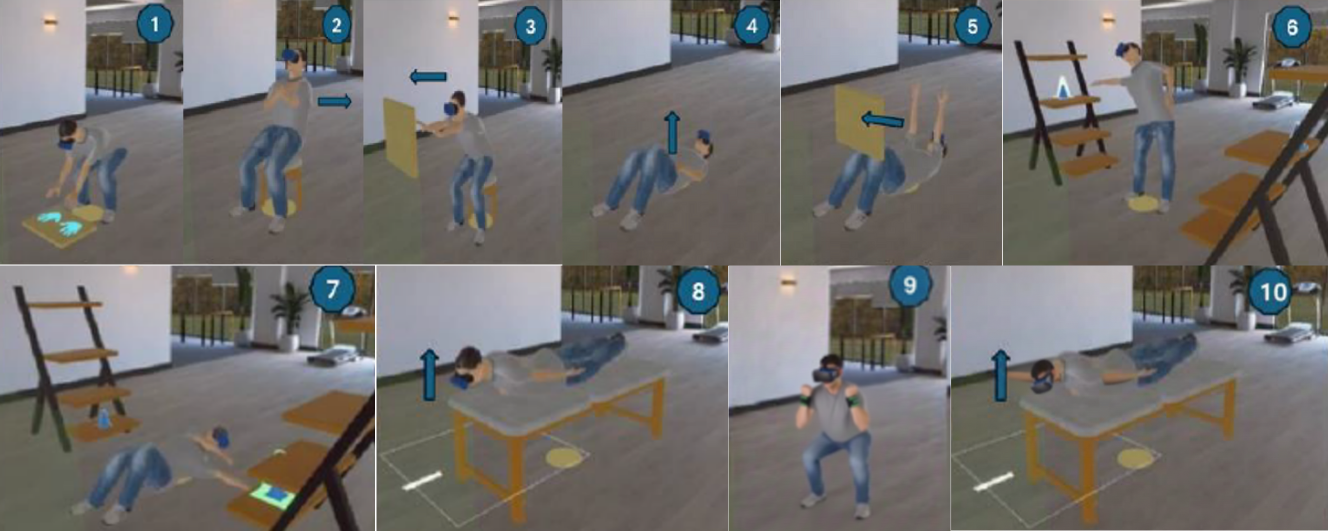
Patients will undergo 12 rehabilitative sessions, each lasting approximately 1 hour, over a 6-week period (2 sessions per week). The treatment regimen will consist of a program of 10 therapeutic exercises utilizing FIVR technology with visual feedback manipulation integrated into each exercise (Figure 4).

***Figure 4:*** *Exercise program with virtual reality****.*** *1) Trunk flexion in standing position; 2) Trunk extension in sitting position; 3) Trunk flexion in sitting position; 4) Glute bridge; 5) Crunch; 6) Trunk inclination in standing position; 7) Trunk rotation in lying position; 8) Trunk extension in lying position; 9) Push press; 10)* Bilateral prone shoulder abduction at 90° with elbows extended (scapular retraction focus).

The exercises are specifically selected to enhance strength, stability, mobility, and flexibility within the abdomino-lumbo-pelvic region and lower extremities [3].

All movements will require synchronization of concentric and eccentric phases with the participant’s respiratory rhythm to standardize exercise pace. Initially, exercises will be performed using body weight alone; however, from the sixth session onward, weights will be progressively introduced, either attached to the wrists or used as dumbbells. Exercise intensity and repetitions will be individualized and adjusted according to the Borg Rating of Perceived Exertion (RPE) scale (6–20), with a recommended intensity between scores of 13 and 17 [4–7]. Table 1 outlines the detailed progression of the VR exercise program, indicating sets, repetitions, intensity levels, and the introduction of weights.


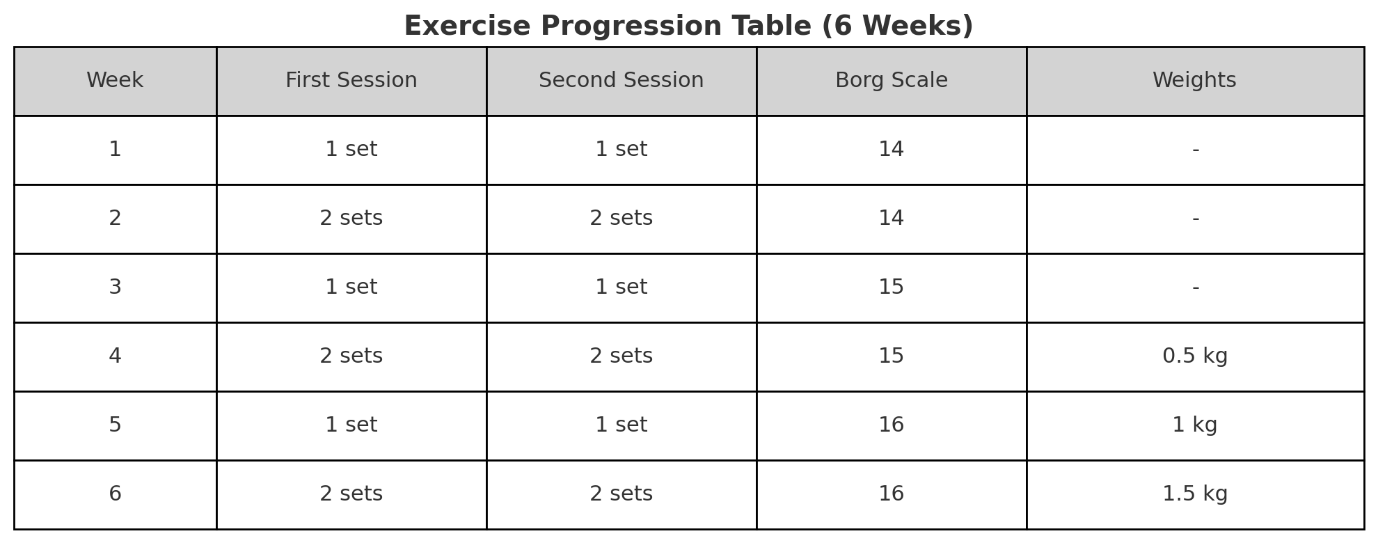
Throughout each session, patients reported pain intensity via a 11-point Numerical Pain Rating Scale (NPRS-11), their RPE, and their subjective feeling of well-being associated with VR usage, with data systematically documented in training logs. Sessions included a consistent 10-minute warm-up and cool-down period and were supervised by a single researcher to maintain methodological consistency.

**Table 1: Exercise progression (sets, Borg 6-20 scale and weights**
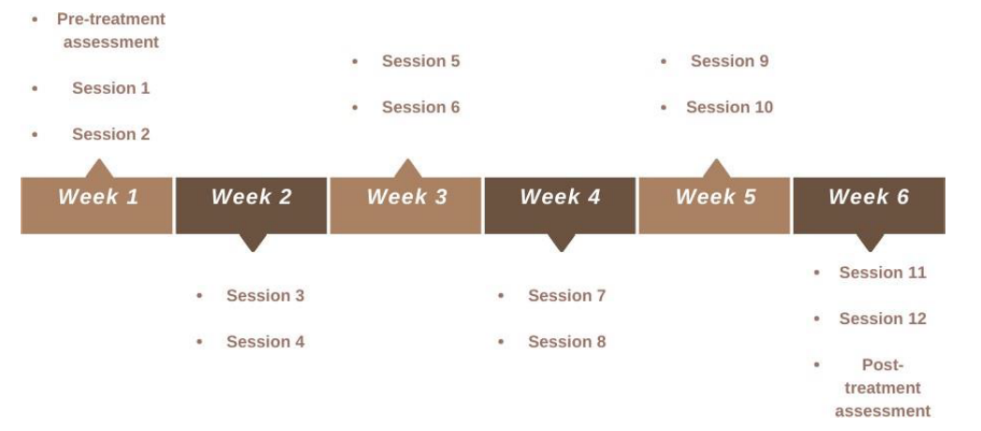


## **FIVR PROGRAM**

The exercise protocol will remain consistent for both groups throughout the 6-week intervention period. The only difference between groups will be the incorporation of FIVR with visual feedback adjustments in one group. Across sessions, the only planned modifications will involve a progressive increase in exercise parameters—including sets, intensity, and weights—intended to incrementally elevate exercise demand and promote physiological adaptations (Table 1).

Each session will be structured into three distinct phases: warm-up, main intervention, and cool-down. Each phase will be described in detail and supported by illustrative images to enhance comprehension and facilitate reproducibility of the study protocol.

### **Warm up**

All these mobility exercises will be performed without weight, free of pain, with respiratory coordination, and with an approximate duration of 5 minutes.

1. **Cervical Mobility:**

- **Double chin/deep flexors (15 seconds):** In a standing position, the participant will be asked to bring their chin inward, "creating a double chin," hold for one second, and then return to the initial position.

**
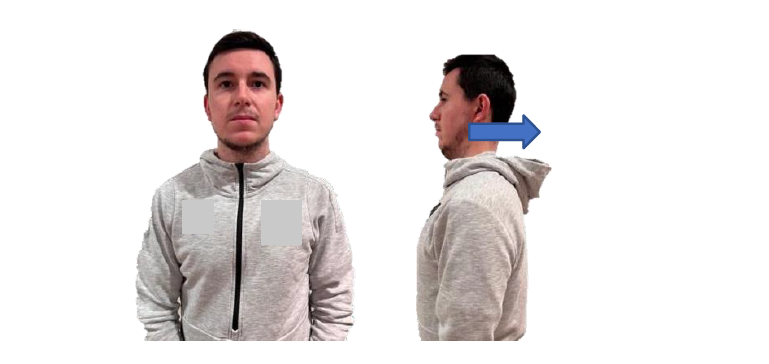
**

- **Cervical inclinations and rotations (15 seconds):** This can be done in both standing and sitting positions.


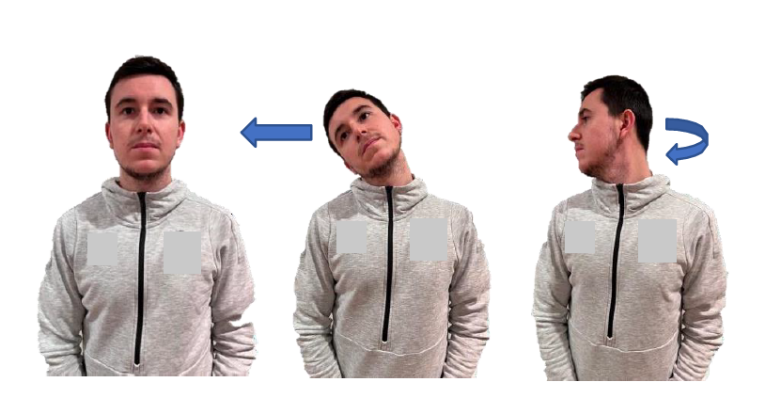


1. **Upper Extremity Mobility**

- **Shoulder flexion-extension/circumduction (15 seconds):** In a standing position, with hands on both sides of the hips, the participant will be asked to bring their arms forward until the hand is above the head. On the return, the arms will be brought backward, and after 2 repetitions, a complete circle will be made.

**
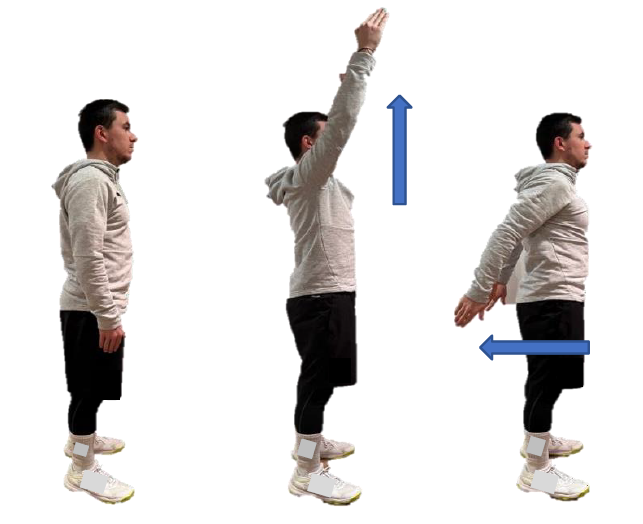
**

-
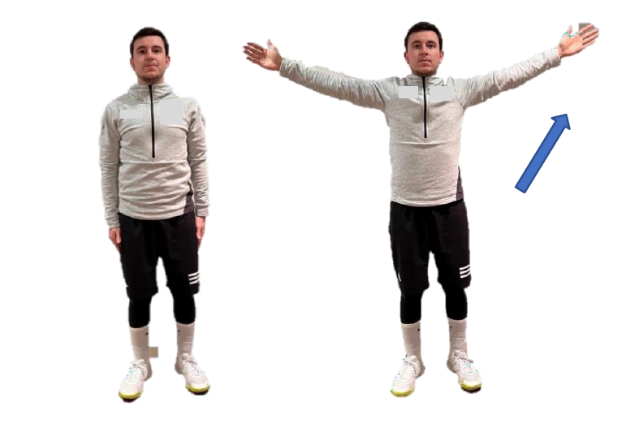
**Abductions/adductions in the frontal and sagittal planes of the shoulder (15 seconds):** In a standing position, with hands on both sides of the hips, the participant will be asked to move their arms laterally until the hand is above the head. Subsequently, they will return to the initial position.

1. **Lumbo-pelvic Mobility:**

-
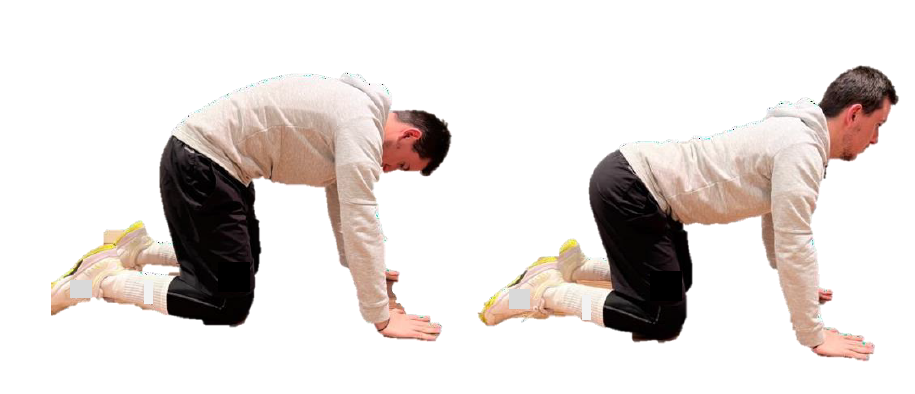
**Cat-Cow Stretch (20 seconds):** In quadruped position, start with pelvic anteversion (tilting the pelvis forward) and slightly lifting the head to eliminate dorsal kyphosis. Then, perform pelvic retroversion (tilting the pelvis backward) while lowering the head and looking toward the navel, arching and increasing dorsal kyphosis.
- **
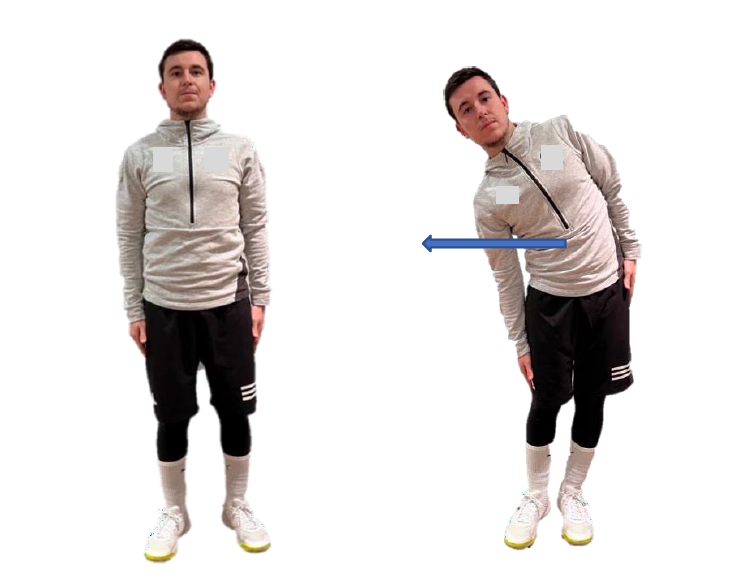
Lumbar inclinations (20 seconds):** In a standing position, with hands on both sides of the hips, the participant will lean one arm toward the knee, performing that lumbar inclination.
- **Lumbar Rotations (20 seconds):** In a standing position, with hands on hips, perform a rotation while keeping the pelvic area fixed.


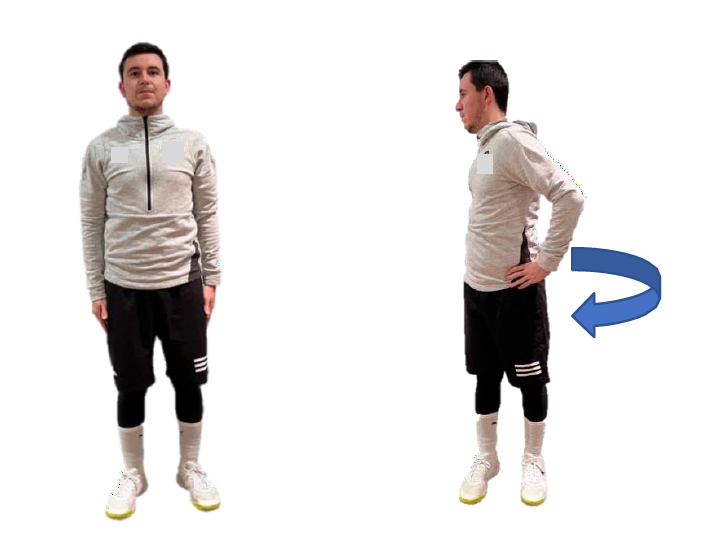


1. **Lower Extremity Mobility**

- **Hip/knee flexion (20 seconds):** In a standing position with or without wall support, bring the knees up toward the chest. Alternate between legs.


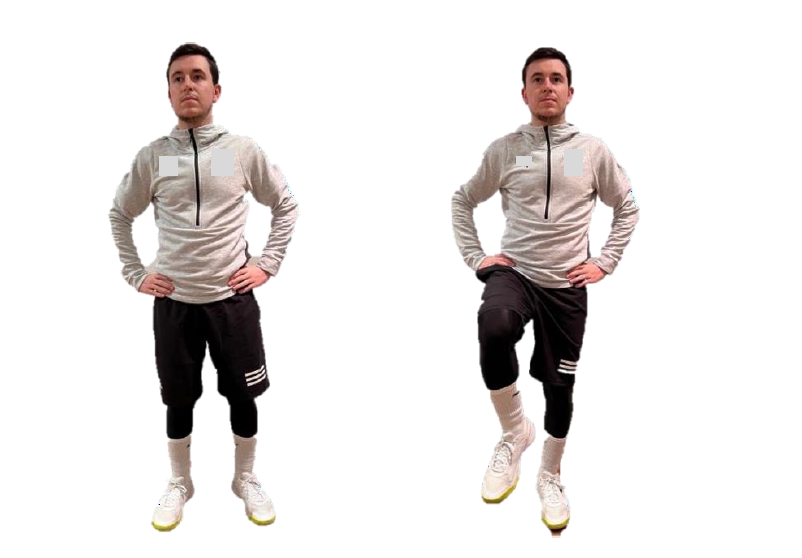


- **Hip abductions (20 seconds):** In a standing position with or without wall support, move one leg laterally and return to the initial position. Alternate between legs.


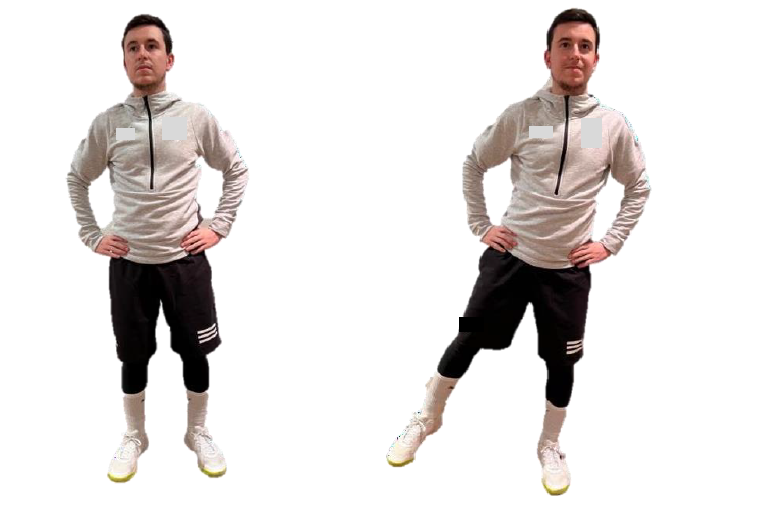


- **Hip extensions (20 seconds):** In a standing position with or without wall support, extend one leg backward. Alternate between legs.


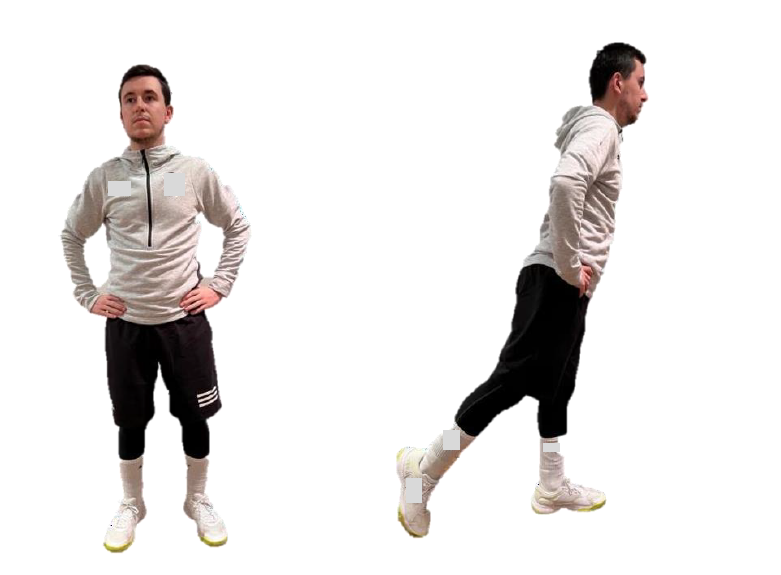


### **Main Intervention**

The main intervention will consist of 10 exercises performed either with or without FIVR, depending on the participant's assigned group. Below, we will provide a detailed description of each exercise. Standardized instructions will be delivered to participants via pre-recorded video demonstrations and audio explanations through speakers. The exercises can be viewed in the following video: <https://drive.google.com/file/d/1bszDrgOKrwQzkJtK3Di_bNA8g0HGn7iV/view?usp=sharing>. The exercises included in the program will be the following ones:

1.
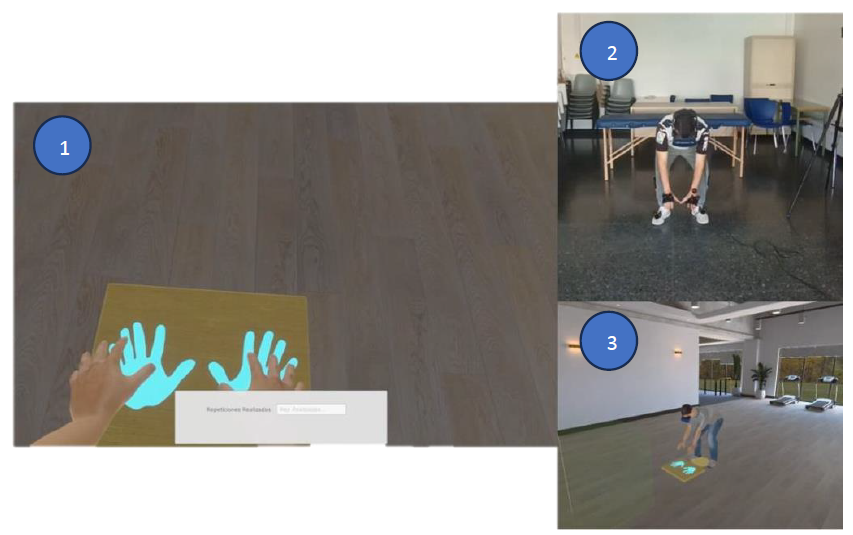
**TRUNK FLEXION IN STANDING POSITION:** In a standing position, perform a maximum knee flexion of 90 degrees, and along with trunk flexion, try to touch virtual footprints that will appear on both the ground (at 0 degrees) and the wall (at 45 degrees). As you approach them, gradually they will increase their size. Here, you will work on the lumbar muscles, glutes, quadriceps, and hamstrings. (See the following figure in 3 perspectives: 1) patient perspective in first-person, 2) real life and 3) research perspective watching the screen.
2.
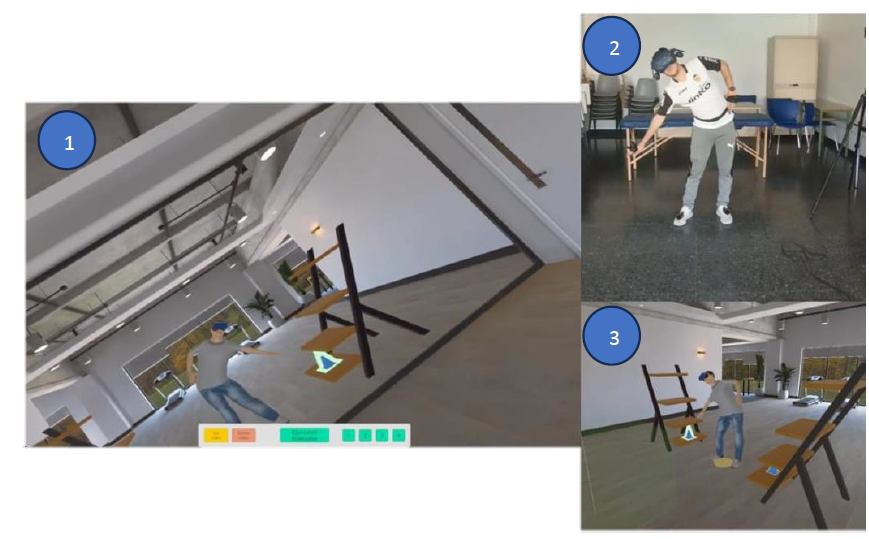
 **LUMBAR INCLINATIONS IN STANDING POSITION:** In a standing position, perform a lateral lumbar flexion while observing the arm throughout the movement. The arm on the side not involved in the lateral flexion will be placed in a jar position. During each lateral flexion, the arm will be positioned at different angles: 0 degrees, 45 degrees, 90 degrees, and 105 degrees. In VR, a cone will appear at each angle, increasing in size as you approach it with your hand. Here, you will work on unilateral lumbar muscles primarily. (See the following figure in 3 perspectives: 1) patient perspective in first-person, 2) real life and 3) research perspective watching the screen.
3. **LUMBAR EXTENSION IN SITTING POSITION:** In a seated position on a stool, with arms crossed on the chest, you will be asked to perform a lumbar extension, hold for one second, and return to the initial position. In VR, as the trunk moves backward, a virtual bar will rise toward the ceiling. Here, you will work on the lumbar and abdominal muscles. (See the following figure in 3 perspectives: 1) patient perspective in first-person, 2) real life and 3) research perspective
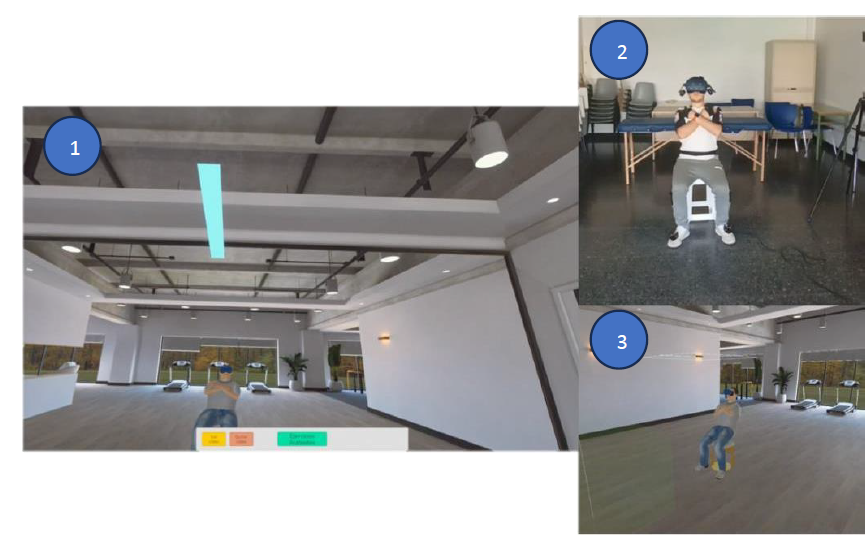
watching the screen.
4. **LUMBAR FLEXION IN SITTING POSITION:** In a seated position on a stool, with arms fully extended, you will be asked to perform a lumbar flexion, hold for one second, and return to the initial position. In VR, as the trunk moves forward, attempting to touch footprints with the hands, they will progressively increase in size. Here, you will work on the lumbar and abdominal muscles. (See the following figure in 3 perspectives: 1) patient perspective in first-person, 2) real life and 3) research perspective watching the screen.


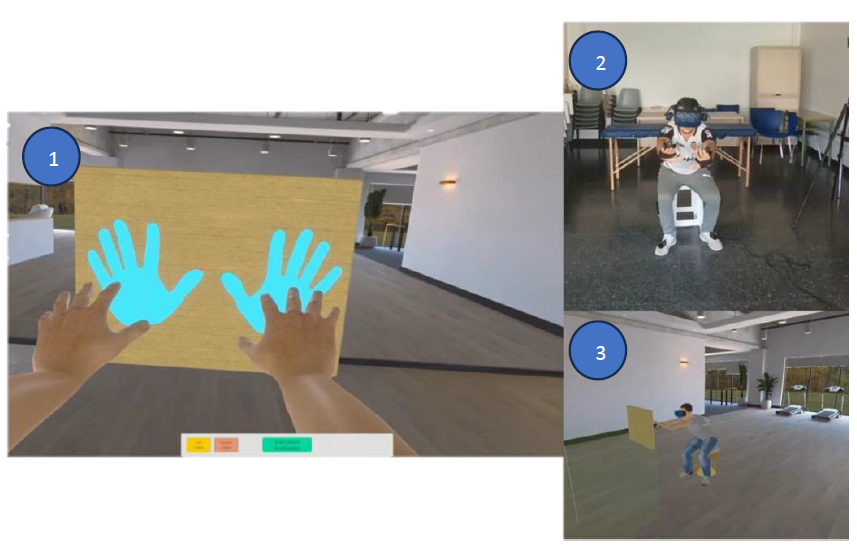


1.
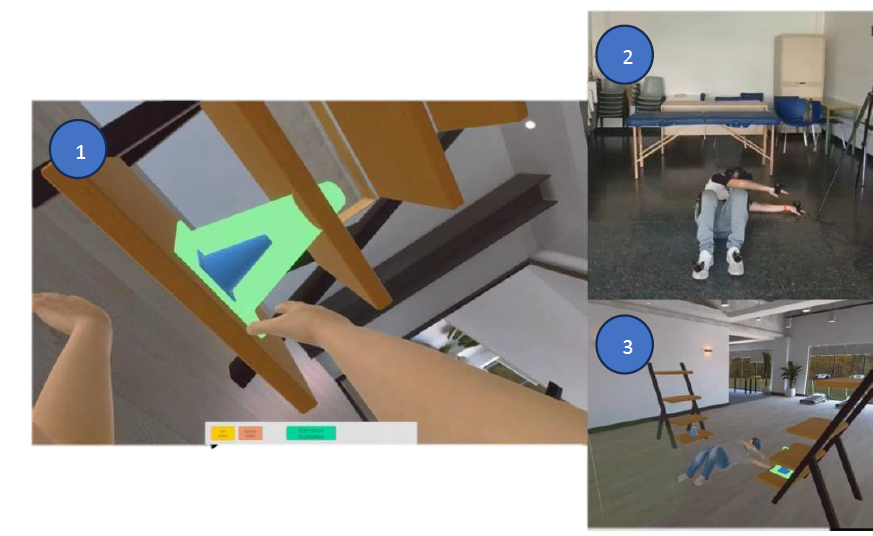
**LUMBAR ROTATION IN LYING POSITION:** You will lie down in a supine position with knees bent and the arm that will perform the rotation flexed. The other arm will be supported on the ground. Next, with the neck in a neutral position, they will lift the trunk and perform a trunk rotation, bringing the elevated hand to the opposite side. In VR, a cone will appear diagonally, progressively increasing in size as the patient approaches. This exercise targets the oblique abdominal muscles. (See the following figure in 3 perspectives: 1) patient perspective in first-person, 2) real life and 3) research perspective watching the screen.
2. **GLUTE BRIDGE:** You will lie down in a supine position with knees bent, a band between the knees, and arms on both sides of the hips. With the neck in a neutral position, they will be asked to elevate the hip as high as possible and hold for a few seconds. In VR, as the hip rises, a virtual bar displayed in the goggles will also rise. In this exercise targets the lumbar muscles, glutes, and hamstrings. (See the following figure in 3 perspectives: 1) patient perspective in first-person, 2) real life and 3) research perspective watching the screen.
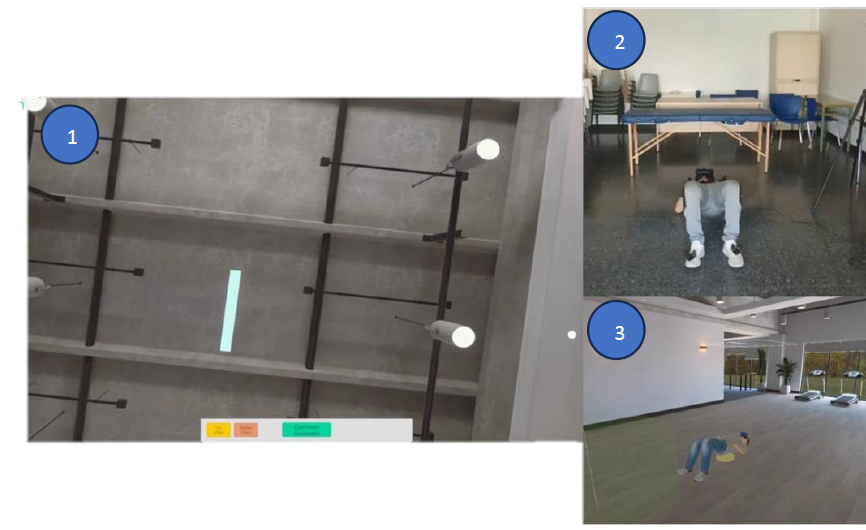

3.
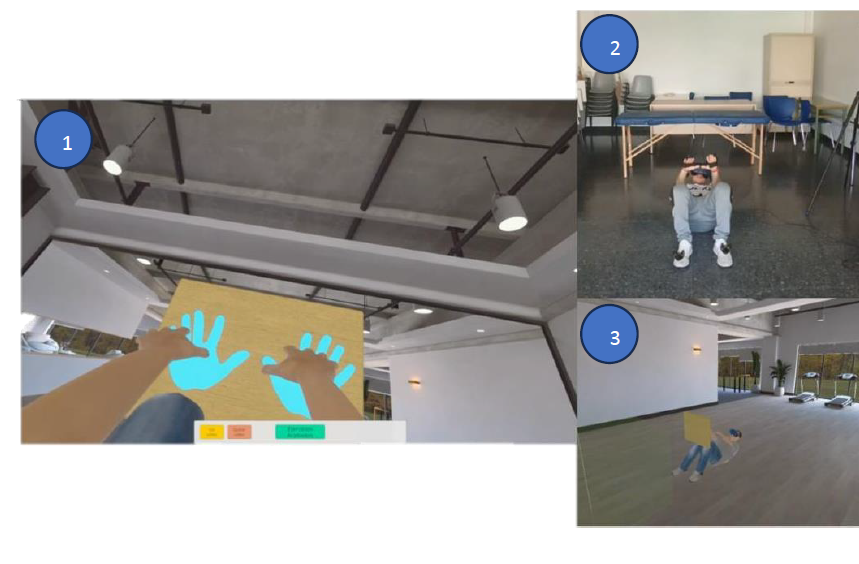
**CRUNCH:** You will lie down in a supine position with knees bent and arms raised. With the neck in a neutral position, they will slightly lift the trunk and try to touch some footprints above their knees which progressively will be increasing in size as the patient approaches with their hands. The descent should always be slow. This exercise primarily targets the abdominal muscles. (See the following figure in 3 perspectives: 1) patient perspective in first-person, 2) real life and 3) research perspective watching the screen.
4. **LUMBAR EXTENSION IN LYING POSITION:** You will lie down in a prone position with knees extended and arms on both sides of the hips. With the neck in a neutral position, you will be asked to lift the shoulders, arms, and head off the mat, performing a lumbar extension. In VR, as the trunk and head rise, you will see a virtual bar rising in the goggles. This exercise targets the lumbar muscles, glutes, and hamstrings. (See the following figure in 3 perspectives: 1) patient perspective in first-person, 2) real life and 3) research perspective watching the screen.


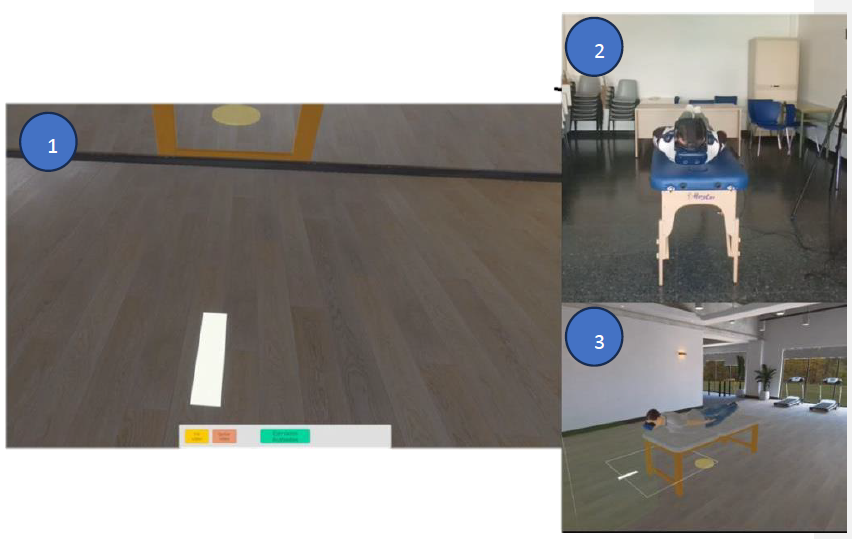


1. **PUSH PRESS:** You will stand with feet shoulder-width apart. With hands at shoulder height and elbows under wrists, perform a partial squat (≈45–60° knee flexion) keeping a neutral spine. As you extend hips and knees to stand, simultaneously press both arms overhead until full elbow extension without lumbar hyperextension or rib flare. Return the hands to shoulder height and repeat. In VR, a first-person avatar mirrors your movement in real time (embodiment with veridical feedback). Primary targets: quadriceps, gluteus maximus, anterior/middle deltoid, triceps; core as stabiliser. *(See figure in two perspectives: 1) first-person patient view, 2) real life.)*


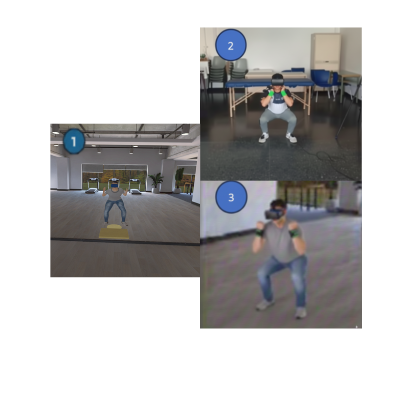


1. **BILATERAL PRONE SHOULDER ABDUCTION AT 90° WITH ELBOWS EXTENDED (scapular retraction focus)**: You will lie prone with legs extended and the forehead supported to keep the neck neutral. Position both arms abducted to ≈90° (T-shape), elbows fully extended. From this start position, lift both straight arms off the surface simultaneously in the frontal/scapular plane while actively retracting and slightly depressing the scapulae (“draw the shoulder blades together and down”). Avoid lumbar extension and rib flare; keep cervical and thoracic alignment neutral. Hold 1–2 seconds at end range, then lower with control. *Primary targets:* middle trapezius and rhomboids; posterior deltoid and supraspinatus as prime movers; lower trapezius and serratus anterior as stabilisers; spinal extensors as synergists. *(See figure in two perspectives: 1) first-person view showing the grey table surface, 2) real-world execution.)*


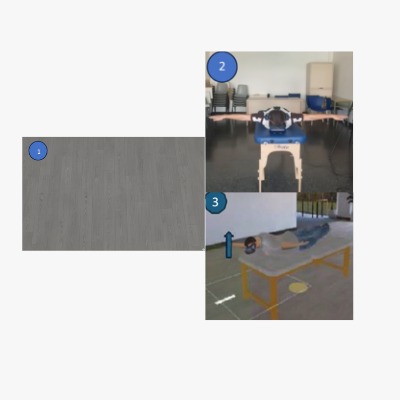


### **COOL-DOWN:**

All these exercises were mobility exercises, stretches, and breathing exercises aimed at relaxing the area and lowering heart rate. It lasts approximately 5 minutes.

- **LUMBAR STRETCH IN ROTATION POSITION (1.5 minutes):** In a supine position with knees bent and arms out to the sides, we bring the knees to one side while looking to the opposite side. You will perform 5 repetitions on each side, and on the last one, hold the stretch for 10 seconds.


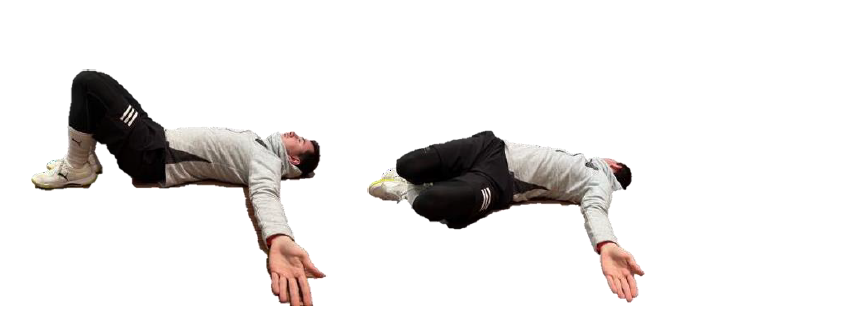


- **LUMBAR STRETCH IN EGG POSITION (1.3 minutes):** In a supine position (lying face-up) with knees bent, hold onto the legs and bring them to the chest, performing 10 bounces, and on the last one, hold the stretch for 10 seconds.


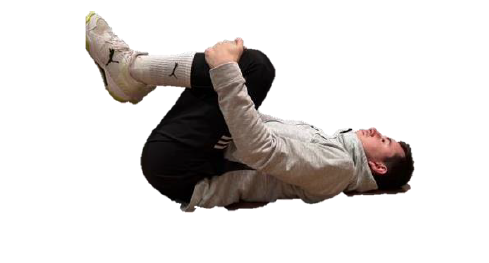


- **HAMSTRING STRETCH (40 seconds)**


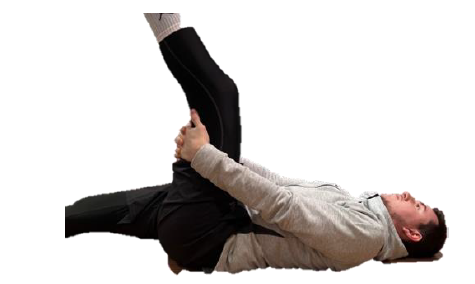


- **BREATHS IN SUPINE POSITION WITH CLOSED EYES (Inhale through the nose and exhale through the mouth).**


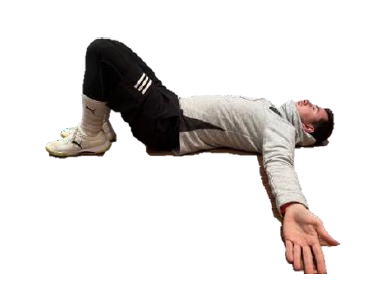


BLIBLIOGRAPHY

1. Jordán-López J, Arguisuelas MD, Doménech J, Peñalver-Barrios ML, Miragall M, Herrero R, et al. Modifying lumbar flexion pain thresholds in patients with chronic low back pain through visual-proprioceptive manipulation with virtual reality: a cross-sectional study. J Neuroeng Rehabil. 2025;22: 138. doi:10.1186/s12984-025-01664-2

2. Jordán-López J, Arguisuelas MD, Doménech J, Peñalver-Barrios ML, Miragall M, Herrero R, et al. Visual feedback manipulation in virtual reality alters movement-evoked pain perception in chronic low back pain. Sci Rep. 2025;15: 20372. doi:10.1038/s41598-025-08094-z

3. Hayden JA, Ellis J, Ogilvie R, Stewart SA, Bagg MK, Stanojevic S, et al. Some types of exercise are more effective than others in people with chronic low back pain: a network meta-analysis. J Physiother. 2021;67: 252–262. doi:10.1016/j.jphys.2021.09.004

4. Tiggemann CL, Pietta-Dias C, Schoenell MCW, Noll M, Alberton CL, Pinto RS, et al. Rating of Perceived Exertion as a Method to Determine Training Loads in Strength Training in Elderly Women: A Randomized Controlled Study. Int J Environ Res Public Health. 2021;18: 7892. doi:10.3390/ijerph18157892

5. Morishita S, Tsubaki A, Nakamura M, Nashimoto S, Fu JB, Onishi H. Rating of perceived exertion on resistance training in elderly subjects. Expert Rev Cardiovasc Ther. 2019;17: 135–142. doi:10.1080/14779072.2019.1561278

6. Row BS, Knutzen KM, Skogsberg NJ. Regulating Explosive Resistance Training Intensity Using the Rating of Perceived Exertion. J Strength Cond Res. 2012;26: 664–671. doi:10.1519/JSC.0b013e31822ac367

7. Row Lazzarini BS, Dropp MW, Lloyd W. Upper-Extremity Explosive Resistance Training With Older Adults Can Be Regulated Using the Rating of Perceived Exertion. J Strength Cond Res. 2017;31: 831–836. doi:10.1519/JSC.0000000000001520
