# Supplementary material for: Effectiveness of a fully immersive virtual reality-based therapeutic exercise programme with altered visual feedback in patients with fibromyalgia: A study protocol for a randomised controlled trial
Source: PLoS One. 2026 Jun 4;21(6):e0348346. doi: 10.1371/journal.pone.0348346 (PMC13235889; doi:10.1371/journal.pone.0348346)
Supplement: S4 Document — (PDF) [file pone.0348346.s005.pdf]

Notification of FAVOURABLE Opinion from the Biomedical Research Ethics Committee for Research Involving Humans:

Reference	CEEI25/643
Principal Investigator	[redacted]
Project Title:	Effectiveness of Immersive Virtual Reality in Patients with Fibromyalgia: A Randomized Clinical Trial
Meeting date:	10 February 2025
Clinical experimentation with human participants	Clinical experimentation with human participants: Favourable
Collection of human biological samples	Not requested
Use of personal data via anonymous questionnaire	Favourable
Use of personal data via interview or non-anonymous questionnaire	Not requested
Review of medical records and/or data repositories	Not requested
Validity	5 years

[redacted]

Signed,
[redacted]
Chair of the Biomedical Research Ethics Committee

As Principal Investigator, you are required to notify the Biomedical Research Ethics Committee of any substantial change (increase in sample size, inclusion of new recruitment centres, inclusion of new researchers in the project, etc.) to the approved research design. To report any of these or other circumstances, you must submit an addendum to this project through the official channels.
