# Supplementary material for: Effectiveness of a fully immersive virtual reality-based therapeutic exercise programme with altered visual feedback in patients with fibromyalgia: A study protocol for a randomised controlled trial
Source: PLoS One. 2026 Jun 4;21(6):e0348346. doi: 10.1371/journal.pone.0348346 (PMC13235889; doi:10.1371/journal.pone.0348346)
Supplement: S5 Document — (PDF) [file pone.0348346.s006.pdf]

Comunicación de dictamen **FAVORABLE** del Comité de Ética para la Investigación con Humanos

Referencia	**INFORME CEEI25/643**
Investigador Principal	[redacted]
Título del Proyecto	EFICACIA DE LA REALIDAD VIRTUAL INMERSIVA EN PACIENTES CON FIBROMIALGIA: UN ENSAYO CLÍNICO ALEATORIZADO
Fecha de la reunión	10 de febrero de 2025
Experimentación Clínica con Seres Humanos	Favorable
Recogida de muestras biológicas de origen humano	No solicitado
Uso de datos personales mediante cuestionario anónimo	Favorable
Uso de datos personales mediante entrevista o cuestionario no anónimo	No solicitado
Consulta de Historias Clínicas y/o repositorio de datos	No solicitado
Vigencia	5 años

[redacted]

Fdo. [redacted]

Presidenta del comité de Ética para la Investigación Biomédica

Como investigador principal, estás obligado a notificar al Comité de Ética de la Investigación Biomédica, cualquier cambio substancial (aumento del tamaño muestral, inclusión de nuevos centros para la captación de participantes, inclusión de nuevos investigadores en el proyecto, etc.) en el diseño de la investigación que ha sido aprobada. Para informar de cualquiera de estas u otras circunstancias, deberás presentar una adenda a este proyecto por los cauces oficiales.
