# Supplementary material for: Effectiveness of a fully immersive virtual reality-based therapeutic exercise programme with altered visual feedback in patients with fibromyalgia: A study protocol for a randomised controlled trial
Source: PLoS One. 2026 Jun 4;21(6):e0348346. doi: 10.1371/journal.pone.0348346 (PMC13235889; doi:10.1371/journal.pone.0348346)
Supplement: Attachment — Submitted filename: Comments.docx [file pone.0348346.s008.docx]

**Effectiveness of an immersive virtual reality-based therapeutic exercise programme with altered visual feedback in patients with fibromyalgia: A study protocol for a randomised controlled trial**

**Summary**

The work is a study protocol of a randomized controlled clinical trial research project. The clinical trial is targeted at potentially improving the nonpharmacologic treatment given to fibromyalgia, a very common disease with a substantial impact on global health care and population. In this study, the IVR is designed to manipulate the vision, making the patient feel as though they are experiencing reduced body movement compared to the actual motion.

**Comments**

1. According to your 5th reference (Cabo-Meseguer A, Cerdá-Olmedo G, Trillo-Mata JL. Fibromialgia: prevalencia, perfiles epidemiológicos y costes económicos. Med Clin (Barc). 2017;149:441–448. doi:10.1016/j.medcli.2017.06.008), the annual expenditure of fibromyalgia treatment in Spain is more than 12,993 million euros, but on line 55, you stated 4.2 billion; you need to clarify the discrepancy. Furthermore, your 5th reference is focused on Spain, so if you want to mention figures related to the USA, I recommend you use studies done in the USA.

2. The abbreviations EG and CG are used in lines 122 and 125, but the extended versions are mentioned in176 and 177; correct that.

3. In the “Ethical approval and registration” section, I recommend you include a statement that shows that participants can drop out of the study at any time without any fear of being denied any health service at your hospital or other health care facilities.

4. The authors need to mention the reason for stratifying the patients by strata, considering sex and FM severity in the manuscript.

5. TENS, AE, and RPE are included only as abbreviations; include the extended versions as well.

6. Your study is going to use fully immersive VR technology, but you described it as immersive VR. According to the classification, immersive can be fully or semi-immersive. Therefore, for better clarity, you should consistently use the term fully immersive VR.

**Questions**

1. Wouldn't using different diagnostic criteria for the diagnosis of fibromyalgia affect your statistical findings?
2. What language are you going to use to assess the FIQR; Central Sensitisation Inventory (CSI); Multidimensional Fatigue Inventory (MFI-20); Pittsburgh Sleep Quality Index (PSQI); EQ-5D-5L? and the other scores?
3. Is the presence of comorbidities like hormonal abnormalities, respiratory illnesses profile of the samples going to be assessed?
4. Did you consider concurrent rheumatological disease, such as rheumatoidarthritis or osteoarthritis, as exclusion criteria?
5. What are the parts of the device to be used in the VR treatment?
6. What types of videos or games are going to be used for VR treatment?
7. What are the operational definitions of the “physiological, psychological, and physical outcomes” of fibromyalgia?
8. Why do you want to include only those patients with a pain intensity score of ≥3?
9. What are the comorbidities and/or symptoms that you will consider as  contraindications for IVR and exercise-based interventions?
10. Why did you select 6 weeks for the duration of therapy? And how did you determine the 2 times per week frequency of sessions? And why did you select 60 minutes for the duration of a session?
11. Why did you choose HTC Vive Pro?
12. Why did you choose 20% arm underestimation during flexion and 10% underestimation during extension specifically?
