# Supplementary material for: Effectiveness of a fully immersive virtual reality-based therapeutic exercise programme with altered visual feedback in patients with fibromyalgia: A study protocol for a randomised controlled trial
Source: PLoS One. 2026 Jun 4;21(6):e0348346. doi: 10.1371/journal.pone.0348346 (PMC13235889; doi:10.1371/journal.pone.0348346)
Supplement: Attachment — Submitted filename: RESPONSE TO REVIEWERS.docx [file pone.0348346.s010.docx]

# **RESPONSES TO JOURNAL REQUIREMENTS**

## **JOURNAL REQUIREMENTS**

### **FIRST REQUIREMENT**

“Please ensure that your manuscript meets PLOS ONE's style requirements, including those for file naming. The PLOS ONE style templates can be found at
[https://journals.plos.org/plosone/s/file?id=wjVg/PLOSOne_formatting_sample_main_body.pdf](https://urldefense.com/v3/__https:/track.editorialmanager.com/CL0/https:*2F*2Fjournals.plos.org*2Fplosone*2Fs*2Ffile*3Fid=wjVg*2FPLOSOne_formatting_sample_main_body.pdf/1/010f019c9274933c-0492bf10-244a-41a3-a410-e3093514f811-000000/6IFvXpT0xYyBgmRqH_IDHPSBNRiL1B7di1HOu13z2ks=252__;JSUlJSUlJQ!!D9dNQwwGXtA!V6serKk1QuYcuBdYtPVpSUjEBsm07cPGA0l7GyBJ-UqrluH0pHQIcPUEHlJXJTMubG__PQK4rZKVBNYzsMc$) and [https://journals.plos.org/plosone/s/file?id=ba62/PLOSOne_formatting_sample_title_authors_affiliations.pdf](https://urldefense.com/v3/__https:/track.editorialmanager.com/CL0/https:*2F*2Fjournals.plos.org*2Fplosone*2Fs*2Ffile*3Fid=ba62*2FPLOSOne_formatting_sample_title_authors_affiliations.pdf/1/010f019c9274933c-0492bf10-244a-41a3-a410-e3093514f811-000000/MSG3eYBAvHOeyIEbvHNHNTpHVRoKhTn5Y0PKduvozbE=252__;JSUlJSUlJQ!!D9dNQwwGXtA!V6serKk1QuYcuBdYtPVpSUjEBsm07cPGA0l7GyBJ-UqrluH0pHQIcPUEHlJXJTMubG__PQK4rZKVonaH5yo$)”

**Response**

We have carefully revised the manuscript to ensure compliance with PLOS ONE’s style requirements, using the journal’s formatting sample files for the main body and for the title, authors, and affiliations page as reference guides. Specifically, we reviewed the manuscript structure, title page, author and affiliation formatting, section layout, and file naming, and we amended these elements where needed so that the revised submission conforms to PLOS ONE formatting requirements.

### **SECOND REQUIREMENT**

“Your ethics statement should only appear in the Methods section of your manuscript. If your ethics statement is written in any section besides the Methods, please delete it from any other section.”

**Response**

We confirm that the ethics statement is presented in the Methods section of the manuscript, in accordance with PLOS ONE’s requirements. We also ensured that the ethics information is reported consistently across the revised submission materials.

### **THIRD REQUIREMENT**

“Please provide a complete Data Availability Statement in the submission form, ensuring you include all necessary access information or a reason for why you are unable to make your data freely accessible. If your research concerns only data provided within your submission, please write "All data are in the manuscript and/or supporting information files" as your Data Availability Statement.”

**Response**

We have provided a complete Data Availability Statement in the submission form. As this manuscript is a study protocol and does not report study results or generated datasets, the statement clarifies that no datasets were generated or analysed for the current article. It also specifies that, upon trial completion, the de-identified dataset, accompanying data dictionary, and analysis code will be deposited in Zenodo and made publicly available under an open licence, with DOIs to be provided upon release.

### **FOURTH REQUIREMENT**

“When completing the data availability statement of the submission form, you indicated that you will make your data available on acceptance. We strongly recommend all authors decide on a data sharing plan before acceptance, as the process can be lengthy and hold up publication timelines. Please note that, though access restrictions are acceptable now, your entire data will need to be made freely accessible if your manuscript is accepted for publication. This policy applies to all data except where public deposition would breach compliance with the protocol approved by your research ethics board. If you are unable to adhere to our open data policy, please kindly revise your statement to explain your reasoning and we will seek the editor's input on an exemption. Please be assured that, once you have provided your new statement, the assessment of your exemption will not hold up the peer review process.”

**Response**

We have revised the Data Availability Statement in the submission form to clarify our data-sharing plan in line with PLOS ONE policy. As this manuscript is a study protocol and does not report study results or generated datasets, the revised statement specifies that no datasets were generated or analysed during the current study. It also states that, upon trial completion, the de-identified dataset, accompanying data dictionary, and analysis code will be deposited in Zenodo and made publicly available under an open licence, with the corresponding DOIs to be provided upon release.

### **FIFTH REQUIREMENT**

“We note that the grant information you provided in the ‘Funding Information’ and ‘Financial Disclosure’ sections do not match.

When you resubmit, please ensure that you provide the correct grant numbers for the awards you received for your study in the ‘Funding Information’ section.”

**Response**

Thank you for highlighting this discrepancy. We acknowledge that the grant information reported in the “Funding Information” and “Financial Disclosure” sections was inconsistent. We have reviewed the funding details and will revise the “Funding Information” section at resubmission so that the correct grant number(s) are provided and both sections are fully aligned.

### **SIXTH REQUIREMENT**

“Thank you for stating the following financial disclosure:

This work was supported by grants from the University CEU Cardenal Herrera (GIR25/41).

Please state what role the funders took in the study. If the funders had no role, please state: "The funders had no role in study design, data collection and analysis, decision to publish, or preparation of the manuscript."

If this statement is not correct you must amend it as needed.
Please include this amended Role of Funder statement in your cover letter; we will change the online submission form on your behalf.”

**Response**

Thank you for this clarification. We confirm that the funder’s role was limited to financial support only. The funders had no role in study design, data collection and analysis, decision to publish, or preparation of the manuscript. As requested, we have included the amended Role of Funder statement in the cover letter.

### **SEVENTH AND EIGHTH REQUIREMENT**

“Please amend your authorship list in your manuscript file to include author Juan Francisco Lisón Párraga”

“Please amend the manuscript submission data (via Edit Submission) to include author Juan Francisco Lisón”

**Response**

We have reviewed and updated the authorship information across the submission materials to ensure consistency between the manuscript file and the online submission data. In accordance with the author’s preferred publication name, this author is listed as Juan Francisco Lisón in the manuscript, and the submission metadata have been amended accordingly so that the author details are complete, accurate, and fully aligned across the revised submission.

### **NINTH REQUIREMENT**

“Please amend either the abstract on the online submission form (via Edit Submission) or the abstract in the manuscript so that they are identical.”

**Response**

We have reviewed the abstract in both the manuscript and the online submission form and ensured that the two versions are identical across the revised submission.

### **TENTH REQUIREMENT**

“We note that there is identifying data in the Supporting Information file <S4 Document. Ethics approval (EN).pdf, S5 Document. Ethic approval (ES).pdf>. Due to the inclusion of these potentially identifying data, we have removed this file from your file inventory. Prior to sharing human research participant data, authors should consult with an ethics committee to ensure data are shared in accordance with participant consent and all applicable local laws.

Data sharing should never compromise participant privacy. It is therefore not appropriate to publicly share personally identifiable data on human research participants. The following are examples of data that should not be shared:

- Name, initials, physical address
- Ages more specific than whole numbers
- Internet protocol (IP) address
- Specific dates (birth dates, death dates, examination dates, etc.)
- Contact information such as phone number or email address
- Location data
- ID numbers that seem specific (long numbers, include initials, titled “Hospital ID”) rather than random (small numbers in numerical order)

Data that are not directly identifying may also be inappropriate to share, as in combination they can become identifying. For example, data collected from a small group of participants, vulnerable populations, or private groups should not be shared if they involve indirect identifiers (such as sex, ethnicity, location, etc.) that may risk the identification of study participants.

Additional guidance on preparing raw data for publication can be found in our Data Policy (<https://journals.plos.org/plosone/s/data-availability#loc-human-research-participant data-and-other-sensitive-data>) and in the following article: [http://www.bmj.com/content/340/bmj.c181.long](https://urldefense.com/v3/__http:/www.bmj.com/content/340/bmj.c181.long__;!!D9dNQwwGXtA!V6serKk1QuYcuBdYtPVpSUjEBsm07cPGA0l7GyBJ-UqrluH0pHQIcPUEHlJXJTMubG__PQK4rZKVFhDtVDw$).

Please remove or anonymize all personal information (<specific identifying information in file to be removed>), ensure that the data shared are in accordance with participant consent, and re-upload a fully anonymized data set. Please note that spreadsheet columns with personal information must be removed and not hidden as all hidden columns will appear in the published file.”

**Response**

We have reviewed the supporting ethics approval documents and removed non-essential identifying information prior to re-upload. Specifically, personal names and handwritten signatures have been anonymised in the revised files, while retaining the ethics approval information necessary to document institutional approval. The revised supporting files are therefore fully anonymised for public sharing.

# **RESPONSE TO REVIEWERS**

We would like to express our sincere gratitude to both reviewers for the time, dedication, and thoughtful consideration they have devoted to evaluating our manuscript. We truly appreciate the depth and quality of their comments, which have been highly constructive and insightful.

Their observations and recommendations have allowed us to carefully re-examine several aspects of the protocol, clarify key methodological points, improve the precision of our reporting, and strengthen the scientific rationale underlying our decisions. We believe that addressing these comments has significantly enhanced the clarity, transparency, and overall methodological rigor of the manuscript.

We are grateful for the opportunity to revise our work in light of these valuable suggestions, which have undoubtedly contributed to improving the quality and robustness of the article. For ease of review, we note that all references cited throughout the document are fully listed in the final bibliography section.

## **REVIEWER 1:**

### **MAYOR COMMENTS:**

#### **FIRST MAYOR COMMENT**

“Sample Size Justification: Effect Size Assumptions Need Clarification

The sample size uses an assumed SD = 20 and an MCID of ≈14% on the FIQR to detect between-group differences using ANCOVA.

However:

- The protocol does not state the absolute expected mean FIQR score on which the 14% MCID is computed.
- It is unclear whether the authors powered the study for between-group mean difference or group × time interaction.

Recommendation:
Explicitly report the assumed baseline FIQR mean, the corresponding absolute MCID value, the expected post-intervention difference, and whether the model used in the power calculation aligns with the final analysis approach (ANCOVA vs. repeated-measures ANCOVA). Provide the full numeric parameters used in G*Power for reproducibility.”

**Response**

We thank the reviewer for this important methodological observation. We have revised the manuscript to clarify the assumptions and ensure full reproducibility of the sample size calculation.

Specifically, we now report the assumed baseline FIQR mean used to operationalise the ≈14% MCID, and we translate this to an absolute between-group difference. Assuming a baseline mean FIQR score of 65 points, a 14% difference corresponds to 9.1 FIQR points. With an assumed SD of 20, this corresponds to an effect size of d ≈ 0.46 and Cohen’s f = 0.23.

We also clarify that the trial was powered to detect the baseline-adjusted between-group difference at the post-intervention assessment (i.e., not a group × time interaction). Although the a priori calculation was performed using an ANCOVA framework, the primary analysis will use a prespecified two-timepoint linear mixed-effects model; the post-intervention between-group contrast from this model targets the same baseline-adjusted post-intervention comparison as the ANCOVA used for the sample size calculation.

For reproducibility, the G*Power inputs are now explicitly provided: G*Power 3.1.9.2; test family: F tests; statistical test: ANCOVA: fixed effects, main effects and interactions; number of groups: 2; number of covariates: 1 (baseline FIQR); effect size f = 0.23; α = 0.05 (two-sided); power (1–β) = 0.80; allocation ratio 1:1; total N = 64 (32 per group), inflated to N = 80 to allow for 20% dropout.

Accordingly, the revised manuscript text now reads as follows:

“Sample size was calculated using G*Power 3.1.9.2 based on standard methodological guidelines. The primary outcome is the impact of FM on patients' lives, assessed with FIQR. The minimum clinically important difference between groups was set at ≈14%, based on previous research [37]. Assuming a baseline mean FIQR score of 65 points, this corresponds to an absolute between-group difference of 9.1 FIQR points. With α = 0.05 (two-sided), 80% power, and an assumed SD of 20, the trial was powered to detect this baseline-adjusted post-intervention between-group difference using an ANCOVA framework with baseline FIQR as a covariate. This corresponds to d ≈ 0.46 (9.1/20), equivalent to Cohen’s f = 0.23. G*Power inputs were: test family = F tests; statistical test = ANCOVA: fixed effects, main effects and interactions; number of groups = 2; number of covariates = 1 (baseline FIQR); effect size f = 0.23; α = 0.05; power (1−β) = 0.80. This yielded 32 participants per group (64 total). Allowing for 20% dropout, the target sample was increased to 80 participants. This calculation was designed to detect the baseline-adjusted between-group difference in FIQR at the post-intervention assessment and corresponds to the post-intervention between-group contrast estimated from the prespecified two-time point LMM.”

#### **SECOND MAYOR COMMENT**

“Choice of Analysis Method: Repeated-Measures ANCOVA May Not Be Appropriate
The plan states that two-way repeated-measures ANCOVA will be used, adjusting for covariates (sex, FIQR level, baseline scores).

Two key concerns:

- Repeated-measures ANCOVA is not a standard or well-defined model.
- Adjusting for baseline within a repeated-measures framework typically leads to statistical redundancy and may violate model assumptions.”

Recommendation:

Use a linear mixed-effects model (LMM), which is the standard for RCTs with repeated measures, missing data handled under MAR, and covariate adjustment. LMMs also allow random intercepts (and slopes if appropriate), avoid sphericity assumptions, and appropriately model within-subject correlation.

**Response**

We thank the reviewer for this valuable statistical recommendation. We agree that the term “two-way repeated-measures ANCOVA” is not a standard, well-defined framework for longitudinal RCT data and that adjusting for baseline within a repeated-measures ANCOVA structure may be statistically redundant and conceptually unclear.

In response, we have revised the analysis plan to use linear mixed-effects models (LMMs), which are the standard approach for repeated measures in RCTs. The LMM will include fixed effects for group, time, and the group × time interaction, with a participant-specific random intercept to account for within-subject correlation, and adjustment for prespecified covariates (sex and baseline FM severity level, where applicable).

Given that outcomes are assessed at baseline and post-intervention only, the primary treatment effect is the between-group difference in change from baseline to post-intervention (i.e., the group × time interaction). For this two-timepoint setting, we specify a constrained longitudinal data analysis (cLDA) parameterisation (baseline means constrained equal), in which the treatment effect can be expressed as the post-intervention between-group contrast and aligns with the baseline-adjusted ANCOVA estimand used for the sample size calculation.

Accordingly, we have revised the “Statistical methods” section, and the manuscript now reads as follows:

“Primary and secondary outcomes will be analysed using linear mixed-effects models (LMMs), including fixed effects for group, time, and the group × time interaction, with a random intercept for participants to account for within-subject correlation. Outcomes are assessed at baseline and post-intervention only. Models will be adjusted for prespecified covariates (sex and FM severity level), with covariate adjustment intentionally restricted to this small prespecified set to minimise overfitting and preserve model stability given the planned sample size. Baseline outcome values are accommodated within the two-time point longitudinal model, rather than entered as separate baseline covariates. Other baseline variables (e.g., medication use and other clinical characteristics) will be summarised descriptively and explored only in prespecified sensitivity analyses if substantial baseline imbalance is observed; covariates will not be selected data-driven based on statistical significance.

The primary estimand will be the between-group treatment effect from baseline to post-intervention for FIQR. For the two-time point setting, we will use a constrained longitudinal data analysis (cLDA) parametrisation (baseline means constrained equal), in which the treatment effect (difference in change) can be expressed as the post-intervention between-group contrast; this aligns with the baseline-adjusted post-intervention treatment effect used for the sample size calculation.”

#### **THIRD MAYOR COMMENT**

“Multiple Secondary Outcomes: No Adjustment Strategy Specified

The study includes an exceptionally large set of secondary outcomes (psychological scales, sensory testing, mobility tests, physiological measures, IVR tolerance, motivation, and more).

Given the number of endpoints, there is a substantial risk of false positives.
Recommendation:

Specify a plan for multiplicity control, such as:

- Designating key secondary outcomes a priori and controlling FDR across them, or
- Applying hierarchical testing, or
- Clearly stating that secondary outcomes are exploratory without inferential claim.

PLOS ONE’s policies permit exploratory outcomes, but this must be stated unambiguously.”

**Response**

We thank the reviewer for highlighting the important risk of false-positive findings given the large number of secondary endpoints. In response, we have revised the “Statistical methods” section to state unambiguously that the primary outcome (FIQR) is the only confirmatory endpoint and will be assessed at p < 0.05 (two-sided). All secondary outcomes will be treated as exploratory and will be reported with effect estimates and 95% confidence intervals, without confirmatory inferential claims; any p-values for secondary outcomes, if reported, will be interpreted descriptively. This approach directly addresses multiplicity by avoiding confirmatory hypothesis testing across the large set of secondary endpoints.

Accordingly, the revised manuscript text now reads as follows:

“Given the large number of secondary outcomes, all secondary outcomes will be treated as exploratory and will be reported with effect estimates and 95% confidence intervals, without confirmatory inferential claims.”

#### **FOURTH MAYOR COMMENT**

“Handling of Missing Data: Multiple Imputation Needs Additional Specification
The protocol states that multiple imputations will be used assuming data missing at random (MAR).
Issues requiring clarification:
• The number of imputations is not specified.
• The imputation model variables are not described.
• MAR assumption justification is absent.
• For longitudinal outcomes, multilevel imputation or model-based approaches are usually preferred.
Recommendation:
Specify:
1. Number of imputations (≥20 recommended).
2. Variables included in the imputation model (all predictors, outcomes, and auxiliary variables).
3. Whether imputation will be performed separately by randomized group.
4. Whether imputation will incorporate repeated-measures structure (e.g., MICE with time indicators).”

**Response**

We thank the reviewer for this important request for additional detail regarding our missing-data strategy. We agree that the multiple imputation (MI) procedure should be fully specified for reproducibility and that, for longitudinal outcomes, model-based approaches are generally preferred.

Accordingly, we have revised the “Statistical methods” section to clarify that missing data will be handled primarily using a likelihood-based approach: maximum likelihood estimation within the prespecified linear mixed-effects model (LMM), which uses all available repeated-measures data and yields valid inference under the missing-at-random (MAR) assumption. To make the MAR assumption more explicit, we also added that we will (i) document reasons for missingness when available and (ii) compare baseline characteristics between participants with complete versus incomplete follow-up.

In addition, we have specified MI as a sensitivity analysis and now provide the full MI parameters requested. Specifically:

1. We will generate m = 30 imputed datasets (≥20 as recommended).
2. The imputation model will include all variables used in the primary analyses, namely outcomes at all assessment timepoints, baseline values, prespecified covariates (sex and FM severity level), and time indicators, as well as auxiliary variables plausibly predictive of missingness and/or outcomes (e.g., baseline symptom burden and other baseline measures collected in the trial).
3. To preserve the randomisation structure, MI will be performed separately within each randomised group.
4. To incorporate the repeated-measures nature of the data, MI will be implemented using fully conditional specification / MICE with time indicators and outcome values from other timepoints included as predictors, thereby respecting within-subject information across assessments. Each imputed dataset will be analysed using the prespecified LMM, and results will be combined using Rubin’s rules.

Accordingly, we have revised the “Statistical methods” section, and the updated text now reads as follows:

“Missing data will be handled primarily via maximum likelihood estimation within the LMM framework under the missing-at-random (MAR) assumption, using all available observations. To support the MAR assumption, we will document reasons for missingness when available and compare baseline characteristics of participants with complete versus incomplete follow-up. As a sensitivity analysis, we will perform multiple imputation using fully conditional specification (FCS/MICE) with m = 30 imputed datasets. The imputation model will include outcomes at both timepoints, baseline values, prespecified covariates (sex and FM severity level), time indicators, and relevant auxiliary variables predictive of missingness and/or outcomes. Imputation will be conducted separately within each randomised group and will incorporate the repeated-measures structure by including time indicators and the corresponding outcome measurement at the other timepoint as predictors. Estimates across imputations will be pooled using Rubin’s rules.”

#### **FIFTH MAYOR COMMENT**

“Randomisation: Stratification and Block Sizes Are Appropriate but Require Clarification
Randomisation uses stratified block permutation across eight strata (sex × FIQR severity clusters) with block sizes 2–4.

Concerns:

- FIQR cluster derives from Pérez-Aranda et al., but the method for real-time assignment of clusters in the trial is not fully described.
- The number of strata (8) for a target N=80 may result in unstable allocation within small strata.

Recommendation:
Report:

- The decision process and data required to assign FIQR cluster at baseline.
- Expected sample per stratum and any minimum-size rule.
- Whether randomisation performance (e.g., imbalance assessment) will be monitored.”

**Response**

We thank the reviewer for this helpful request for clarification regarding our stratified block randomisation.

We have now described the real-time decision process for FIQR severity classification at baseline. Specifically, baseline FIQR will be completed prior to randomisation and scored immediately using item-level responses. FIQR severity level will be assigned at baseline according to Pérez-Aranda et al. (2019) and then collapsed into two prespecified severity levels for stratification (clusters 1–2 vs clusters 3–4), implemented using a prespecified scoring tool (e.g., a pre-programmed spreadsheet/REDCap instrument) to enable real-time classification.

To address the concern that eight strata may be unstable for N = 80, we have reduced the stratification to four strata defined by sex (male/female) × baseline FM severity level (two FIQR-based severity levels). We also clarified that no minimum-size rule will be applied; if a stratum is sparse, the prespecified sequence will still be used, and any residual imbalance will be transparently reported and addressed through the prespecified adjusted analyses.

Finally, we added that randomisation performance will be monitored descriptively by periodically summarising allocation counts by arm overall and within strata (without examining outcomes), and we clarified allocation concealment using SNOSE with an independent data manager holding the randomisation list.

Accordingly, we have revised the “Randomisation and blinding” section, and the updated text now reads as follows:

“Participant recruitment and written informed consent will be obtained by physicians from the “Lifestyle and Health” research group at CEU Cardenal Herrera University. Randomisation will be conducted by an independent researcher using a computer-generated 1:1 sequence with stratified permuted blocks (block size = 2) within strata defined by sex (male/female) and baseline FM severity level (two FIQR-based severity levels derived from clusters 1–2 vs clusters 3–4) [34].

Baseline FIQR will be completed prior to randomisation and scored immediately using item-level responses. FIQR severity level will be assigned at baseline according to Pérez-Aranda et al. (2019) using a prespecified scoring tool (e.g., a pre-programmed spreadsheet/REDCap instrument) to enable real-time classification.

Stratification by sex was chosen because FM is substantially more prevalent in women than in men and, given the planned sample size, chance imbalances could otherwise occur. Stratification by FM severity was prioritised to ensure comparability in baseline symptom burden, an important prognostic factor, and to minimise potential shifts in severity distribution arising from recruitment through a single patient association.

Given the target sample size (N = 80) and the expected predominance of women in fibromyalgia, we anticipate that most participants will fall within the female strata, while male strata may be comparatively small. We do not apply a minimum-size rule for strata; if recruitment within a stratum is sparse, the prespecified sequence will still be applied, and final allocation counts by arm overall and within strata will be reported.

Randomisation performance will be monitored descriptively by periodically summarising allocation counts by arm overall and within strata (without examining outcomes), to identify any unexpected allocation irregularities.

Allocation will be concealed with sequentially numbered, opaque, sealed envelopes (SNOSE) prepared off-site with tamper-evident seals and carbonless copies. After baseline assessment, site staff will open the next envelope in numerical order to assign the EG or CG. The randomisation list will be held by an independent data manager with no role in enrolment or assessment. Any deviation from sequence or envelope integrity will be recorded and investigated.

Outcome assessors and the trial statistician will be blinded to group allocation. Participants and intervention providers cannot be blinded due to the nature of FIVR and will be instructed not to disclose allocation during assessments. Assessments will be performed by personnel without access to scheduling or allocation logs; groups will be labelled A/B until database lock. Unblinding will occur only to address serious safety concerns or protocol deviations with safety implications, following written authorisation from the Principal Investigator; all unblinding events will be logged. The randomisation list will remain with the independent data manager.”

#### **SIXTH MAYOR COMMENT**

“Quantitative Sensory Testing (QST): Statistical Plan Requires Additional Detail

The QST section is detailed in measurement procedures, but statistical treatment lacks specification (PPT, TS, CPM outcomes).

Required clarifications:

- Will PPT, TS, and CPM be analysed as continuous outcomes in ANCOVA/LMM?
- Will outliers (common in QST data) be handled through transformation or robust methods?
- How will bilateral PPT sites be modelled (averaged, modelled separately, or nested within subject)”

**Response**

We thank the reviewer for this helpful request for additional detail regarding the statistical treatment of quantitative sensory testing (QST) outcomes (PPT, TS, and CPM).

We have revised the “Statistical methods” section to explicitly specify that PPT, TS, and CPM will be analysed as continuous outcomes using the prespecified linear mixed-effects model (fixed effects: group, time, and group × time; random intercept for participants; covariate adjustment as prespecified). We also clarified how each QST outcome will be derived: PPT will be summarised per site as the mean of three trials, while TS and CPM will be computed from pain ratings recorded at the first and last repetition (last − first).

Regarding the modelling of measurement sites, we clarified that QST is obtained unilaterally on the dominant side at the hand and lumbar region; therefore, bilateral modelling is not applicable. We also added an explicit plan for handling features common in QST data, including potential skewness and extreme values: distributions and model diagnostics will be inspected, transformations (e.g., log-transformation for PPT) will be used if assumptions are not met, and sensitivity analyses will be conducted to assess robustness. Finally, we clarified that refusals to complete QST will be treated as missing and that algometer ceiling values will be recorded at the device maximum and flagged, with sensitivity analyses to evaluate their impact.

Accordingly, we have revised the “Statistical methods” section to incorporate these clarifications, and the updated text now reads as follows:

“Quantitative sensory testing (QST) outcomes (pressure pain threshold [PPT], temporal summation [TS], and conditioned pain modulation [CPM]) will be analysed as continuous outcomes using the prespecified LMM (fixed effects: group, time, and group × time; random intercept for participants), with covariate adjustment as prespecified. QST will be obtained unilaterally on the dominant side at the hand and lumbar region; therefore, bilateral modelling is not applicable. PPT will be summarised per site as the mean of three trials. TS and CPM outcomes will be derived from pain ratings collected at the first and last repetition, computed as (last − first). If a participant declines QST testing or a measure cannot be completed, the corresponding QST outcome will be treated as missing. If the maximum limit of the algometer is reached, the value will be recorded at the device maximum and flagged; sensitivity analyses will assess the impact of these ceiling values. Distributions and model diagnostics will be inspected; if assumptions are not met, appropriate transformations (e.g., log-transformation for PPT) will be applied, and sensitivity analyses will be conducted (e.g., repeating analyses after excluding extreme values identified by a prespecified rule, such as >3 SD from the mean) to evaluate robustness.”

#### **SEVENTH MAYOR COMMENT**

“Per-Protocol vs. Intention-to-Treat

While ITT analysis is planned, the protocol does not describe conditions for per-protocol analysis or adherence thresholds.

Recommendation:

Specify whether:

- A per-protocol set will also be reported.
- Exercise dose or session attendance will be incorporated into sensitivity analyses.”

**Response**

We thank the reviewer for this helpful suggestion regarding the specification of per-protocol analyses and adherence thresholds.

We have revised the “Statistical methods” section to explicitly state that, in addition to the primary intention-to-treat (ITT) analyses, we will report a prespecified per-protocol (PP) analysis as a sensitivity analysis. We defined the PP set a priori as participants who receive their allocated condition, attend at least 75% of the scheduled sessions (i.e., ≥9 of 12 sessions over 6 weeks), and have no major protocol deviations. We also clarified that PP analyses will use the same linear mixed-effects modelling framework as the ITT analyses.

Finally, we added that session attendance (exercise dose) will be incorporated in exploratory sensitivity analyses (e.g., using number/percentage of sessions completed) to assess robustness, with cautious interpretation given that adherence is a post-randomisation variable.

Accordingly, we have revised the “Statistical methods” section, and the updated text now reads as follows:

“Analyses will follow an intention-to-treat (ITT) approach. In addition to the ITT analyses, we will report a prespecified per-protocol (PP) analysis as a sensitivity analysis. The PP set will include participants who attend at least 75% of the scheduled sessions (i.e., ≥9 of 12 sessions over 6 weeks) and who do not present major protocol deviations. PP analyses will use the same linear mixed-effects modelling approach as the primary ITT analysis.”

### **MINOR COMMENTS:**

#### **FIRST MINOR COMMENT**

“Effect Size Reporting Partial eta-squared is appropriate, but confidence intervals for effect sizes should also be reported when possible.”

**Response**

We thank the reviewer for this suggestion. We agree that reporting confidence intervals for effect sizes improves interpretability. We have revised the “Statistical methods” section to specify that, in addition to reporting model-based effect estimates with 95% confidence intervals from the LMM, we will provide 95% confidence intervals for standardised effect sizes (e.g., Hedges’ g) for the primary between-group contrasts when feasible. Where partial eta-squared (ηp²) is reported (where applicable), corresponding 95% confidence intervals will also be provided when available.

Accordingly, we have revised the “Statistical methods” section to incorporate this change, and the updated text now reads as follows:

“Given the large number of secondary outcomes, all secondary outcomes will be treated as exploratory and will be reported with effect estimates and 95% confidence intervals, without confirmatory inferential claims. When feasible, standardised effect sizes (e.g., Hedges’ g) will be reported for the primary between-group contrasts together with 95% confidence intervals; where partial eta-squared (ηp²) is reported for any model terms, corresponding 95% confidence intervals will also be provided when available.

#### **SECOND MINOR COMMENT**

“Covariate Inclusion Adjusting only for sex, baseline FIQR, and baseline outcome may be insufficient if strong prognostic variables exist (e.g., medication use, FM symptom severity, kinesiophobia). Consider prespecifying additional adjustment factors or providing a rationale for the limited covariate set. Covariate adjustment was intentionally restricted to a small set of prespecified variables to avoid overfitting and preserve model stability given the planned sample size. The selected covariates include key prognostic factors and design variables: sex, baseline FIQR, and baseline value of the corresponding outcome.”

**Response**

We thank the reviewer for this helpful comment. We agree that additional prognostic variables (e.g., medication use or other baseline clinical characteristics) may be important in some settings. However, given the planned sample size, we intentionally prespecified a parsimonious covariate set to minimise overfitting and preserve model stability. Accordingly, we clarified that the primary LMMs will adjust only for the prespecified design/prognostic variables used in the trial design (sex and FM severity level).

Baseline values of each outcome are accounted for within the two-timepoint longitudinal model (baseline and post-intervention; cLDA parameterisation), rather than being added as separate baseline covariates. Other baseline variables (including medication use and other clinical characteristics) will be summarised descriptively and explored only in prespecified sensitivity analyses if clinically meaningful baseline imbalance is observed; covariates will not be selected data-driven based on statistical significance.

Accordingly, we have revised the “Statistical methods” section to incorporate this rationale, and the updated text now reads as follows:

“Models will be adjusted for prespecified covariates (sex and FM severity level), with covariate adjustment intentionally restricted to this small prespecified set to minimise overfitting and preserve model stability given the planned sample size. Baseline outcome values are accommodated within the two-timepoint longitudinal model (baseline and post-intervention), rather than entered as separate baseline covariates. Other baseline variables (e.g., medication use and other clinical characteristics) will be summarized descriptively and explored only in prespecified sensitivity analyses if substantial baseline imbalance is observed; covariates will not be selected data-driven based on statistical significance.”

#### **THIRD MINOR COMMENT**

“Rescue Medication The plan instructs participants to avoid analgesics 24 hours before assessments. Clarify whether medication use will be recorded and included as a covariate or descriptive variable.”

**Response**

We thank the reviewer for this helpful clarification. We have revised the manuscript to explicitly state that concomitant medication use will be recorded, including rescue analgesic intake and the timing of the last dose within the preceding 24 hours at each assessment timepoint. Medication use will be summarised descriptively by group and timepoint. In line with our prespecified approach to restrict covariate adjustment and avoid overfitting given the planned sample size, medication use will not be included in the primary adjusted models; however, it will be explored in prespecified sensitivity analyses if substantial between-group imbalance is observed.

Accordingly, we have revised the “Concomitant care” description, and the updated text now reads as follows:

“Medication use, including rescue analgesic intake and the timing of the last dose within the preceding 24 h, will be recorded at each assessment timepoint and summarised descriptively by group and timepoint.”

#### **FOURTH MINOR COMMENT**

“Intervention Fidelity Metrics Session-level variables (e.g., RPE, repetitions, pain pre/post) are being collected but are not included in the analysis plan. Clarify whether these will inform adherence, dose-response exploration, or moderation analysis”.

**Response**

Thank you for this important comment. We agree that this point required clarification. Session-level variables such as RPE, repetitions, and pain before/after each session are being collected to monitor intervention delivery and participant safety/tolerance during the programme. However, these variables are not prespecified as endpoints and will not be included in the inferential statistical analysis plan (e.g., adherence modelling, dose-response exploration, or moderation analyses). In this study, adherence will be operationalised solely as attendance, defined as the number of completed sessions relative to the total number of planned sessions. To avoid overstating the role of these session-level metrics, we have revised the manuscript accordingly and removed wording that could imply that they will be formally analysed statistically.

Accordingly, we have revised the “Intervention” description, and the updated text now reads as follows:

“Main phase (50 min): multi-joint exercises for trunk, upper, and lower limbs in standing, seated, supine, and prone positions. Movements synchronised with breathing to enhance motor control. Intensity regulated by the Borg 6–20 scale targeting RPE 13–17. Sessions 1–5: bodyweight only; from session 6: progressive external loads. Per-exercise volume adjusted to rate of perceived exertion (RPE). At each session we will record NPRS-11, RPE, repetitions, and FIVR-related well-being. These session-level variables will be recorded solely to monitor session delivery and participant response and will not be included in the statistical analysis plan.”

#### **FIFTH MINOR COMMENT**

“Adverse Event Statistical Reporting Although AEs will be reported descriptively, specify whether incidence rates between groups will be compared statistically (risk ratios or risk differences).”

**Response**

We thank the reviewer for this helpful suggestion. We have revised the “Statistical methods” section to explicitly specify how adverse events (AEs) will be reported and compared between groups. In addition to descriptive summaries by group (number of participants with ≥1 AE, total events, severity and relatedness), we will estimate between-group differences in the incidence proportion (participants with ≥1 AE) using risk ratios and risk differences with 95% confidence intervals. Where appropriate, Fisher’s exact test will be used to obtain p-values given the expected small event counts.

Accordingly, the revised manuscript text now reads as follows:

Accordingly, we have revised the “Statistical methods” section, and the updated text now reads as follows:

“Adverse events will be summarised by group (number of participants with ≥1 adverse event, total events, severity and relatedness). Between-group differences in the incidence proportion (participants with ≥1 adverse event) will be estimated using risk ratios and risk differences with 95% confidence intervals; where appropriate, Fisher’s exact test will be used to obtain p-values given expected small counts.”

## **REVIEWER 2:**

### **COMMENTS:**

#### **FIRST COMMENT**

“According to your 5th reference (Cabo-Meseguer A, Cerdá-Olmedo G, Trillo-Mata JL. Fibromialgia: prevalencia, perfiles epidemiológicos y costes económicos. Med Clin (Barc). 2017;149:441–448. doi: 10.1016/j.medcli.2017.06.008), the annual expenditure of fibromyalgia treatment in Spain is more than 12,993 million euros, but on line 55, you stated 4.2 billion; you need to clarify the discrepancy. Furthermore, your 5th reference is focused on Spain, so if you want to mention figures related to the USA, I recommend you use studies done in the USA.”

**Response**

We thank the reviewer for identifying this inconsistency. We have corrected the Spanish estimate to match our cited source (Cabo-Meseguer et al.), which reports an annual cost of €12,993 million, replacing the previously incorrect figure. In addition, we revised the U.S. cost statement to rely on a U.S.-based reference, and we updated the reference list accordingly. These changes improve accuracy and ensure that country-specific estimates are supported by country-specific sources. Accordingly, the relevant sentence in the manuscript has been revised; the text now reads as follows:

“FM prevalence significantly varies by diagnostic criteria, remaining underrecognized clinically [2]. Despite this, FM is the second most common rheumatic condition after osteoarthritis [2,3], with a global prevalence of 2%–8% [3,4]. FM substantially impairs quality of life and functional capacity, significantly increasing healthcare demand and costs [5]. Annual costs associated with FM have been estimated at €12,993 million in Spain [5]. In the United States, an estimated economic burden of over US$20 billion annually has been reported [7]. Indirect societal costs, primarily from lost productivity, are also considerable; approximately 24.3% of patients stop working within five years post-diagnosis [8].”

#### **SECOND COMMENT**

“The abbreviations EG and CG are used in lines 122 and 125, but the extended versions are mentioned in176 and 177; correct that.”

**Response**

Thank you for this observation. We corrected the order of abbreviation use by defining “experimental group (EG)” and “control group (CG)” at their first occurrence (lines 122 and 125) and revised the later text (lines 176–177) to avoid redundant redefinition. This ensures consistent and standard abbreviation usage throughout the manuscript.

#### **THIRD COMMENT**

“In the “Ethical approval and registration” section, I recommend you include a statement that shows that participants can drop out of the study at any time without any fear of being denied any health service at your hospital or other health care facilities.”

**Response**

Thank you for this recommendation. We have added a statement in the “Ethical approval and registration” section clarifying that participation is voluntary and that participants may withdraw at any time without any penalty or impact on their current or future access to healthcare services at any healthcare facility. Given that recruitment and study procedures take place in a patient association (rather than a hospital), we specified that withdrawal will not affect participants’ access to any usual care or services to which they are otherwise entitled. This reinforces participants’ rights and aligns the protocol with ethical best practices.

Accordingly, the “Ethical approval and registration” section has been revised as follows:

“This study complies with the Declaration of Helsinki. The protocol was approved by the Research Ethics Committee of Universidad CEU Cardenal Herrera (CEEI25/643). Participation is voluntary, and written informed consent will be obtained before any study procedure. Participants may withdraw from the study at any time without providing a reason and without any penalty or loss of access to current or future healthcare services at the “Asociación Valenciana de Afectados de Fibromialgia” (AVAFI) or any other healthcare facility. Eligible patients will be informed of objectives, procedures, potential risks, and expected benefits before starting the exercise programme. The protocol is registered at ClinicalTrials.gov (NCT06948500). Protocol version v1.0 (18 September 2025). Personal data will be collected by clinical research coordinators and stored in a secure, password-protected database with access limited to authorised personnel.”

#### **FOURTH COMMENT**

“The authors need to mention the reason for stratifying the patients by strata, considering sex and FM severity in the manuscript.”

**Response**

We thank the reviewer for this suggestion. We have revised the “Randomisation and blinding” description to explicitly justify the choice of stratification factors. Specifically, we added that sex stratification was selected due to the marked sex imbalance typically observed in fibromyalgia and our estimated sample size, which increases the risk of chance imbalance, and that severity stratification was prioritised to ensure baseline comparability in symptom burden (a key prognostic factor) and to mitigate potential recruitment-related shifts when recruiting from a patient association. We also clarified the blocking approach by stating a fixed block size of 2.

Accordingly, the “Randomisation and blinding” section has been revised as follows:

“Participant recruitment and written informed consent will be obtained by physicians from the “Lifestyle and Health” research group at CEU Cardenal Herrera University. Randomisation will be conducted by an independent researcher using a computer-generated 1:1 sequence with stratified permuted blocks (block size =2) within strata defined by sex (male/female) and baseline FM severity level (two FIQR-based severity levels derived from Pérez-Aranda et al., 2019 [34]: clusters 1–2 vs clusters 3–4).

Baseline FIQR will be completed prior to randomisation and scored immediately using item-level responses. FIQR severity level will be assigned at baseline according to Pérez-Aranda et al. (2019) and then collapsed into two prespecified severity levels for stratification: severity level 1 (clusters 1–2) and severity level 2 (clusters 3–4). The data required for this assignment are the baseline FIQR item responses, implemented using a prespecified scoring tool (e.g., a pre-programmed spreadsheet/REDCap instrument) to enable real-time classification.

Stratification by sex was chosen because FM is substantially more prevalent in women than in men and, given the planned sample size, chance imbalances could otherwise occur. Stratification by FM severity was prioritised to ensure comparability in baseline symptom burden, an important prognostic factor, and to minimise potential shifts in severity distribution arising from recruitment through a single patient association.”

#### **FIFTH COMMENT**

“TENS, AE, and RPE are included only as abbreviations; include the extended versions as well.”

**Response**

Thank you for pointing this out. We have revised the manuscript to define these abbreviations at first use by adding the extended terms for transcutaneous electrical nerve stimulation (TENS), adverse events (AEs), and rating of perceived exertion (RPE). We then retained the abbreviations throughout the remainder of the text for consistency.

#### **SIXTH COMMENT**

“Your study is going to use fully immersive VR technology, but you described it as immersive VR. According to the classification, immersive can be fully or semi-immersive. Therefore, for better clarity, you should consistently use the term fully immersive VR.”

**Response**

Thank you for this important clarification. We agree that the term “immersive VR” can encompass both fully immersive and semi-immersive systems. As our intervention uses a head-mounted display and therefore corresponds to fully immersive VR, we have revised the manuscript to use the term “fully immersive VR” consistently throughout (and defined it at first mention). This improves terminological precision and avoids ambiguity.

### **QUESTIONS**:

#### **FIRST QUESTION**

Wouldn't using different diagnostic criteria for the diagnosis of fibromyalgia affect your statistical findings?

**Response**

Using different ACR diagnostic criteria can change the case definition and therefore the clinical profile of included participants; however, we deliberately accepted any established ACR criteria set (1990/2010/2011/2016) to maximise comparability with the existing exercise and VR/exergaming fibromyalgia literature, which spans several decades and does not rely on a single uniform diagnostic framework. [8–12]

Across published trials, eligibility has been based on different ACR versions depending on the period (e.g., major exercise RCTs have used ACR 1990 and/or 2010, [13] while immersive VR plus exercise research has commonly relied on ACR 2010 [14] and more recent immersive VR-based exercise studies use ACR 2016) [15]. Restricting eligibility to only one criteria set would therefore reduce external validity and hinder direct comparison with prior evidence. [13–15]

From a statistical perspective, allowing multiple ACR criteria primarily affects findings through increased clinical heterogeneity rather than directional bias. In a randomised design, criteria should not systematically favour one arm over the other; thus, the main impact is expected to be greater outcome variance and potential attenuation of detectable effects, rather than inflation of treatment effects.

#### **SECOND QUESTION**

What language are you going to use to assess the FIQR; Central Sensitisation Inventory (CSI); Multidimensional Fatigue Inventory (MFI-20); Pittsburgh Sleep Quality Index (PSQI); EQ-5D-5L? and the other scores?

**Response**

We will administer the FIQR, CSI, MFI-20, PSQI, EQ-5D-5L, and the remaining patient-reported outcomes in Spanish (Spain), using validated/cross-culturally adapted Spanish versions whenever available. This is appropriate because the study is conducted in Spain and participants are Spanish-speaking; using validated Spanish versions preserves the instruments’ psychometric properties and reduces measurement error due to translation/comprehension. The Spanish versions of the FIQR, CSI, MFI-20 and PSQI have been validated (including fibromyalgia samples), and the EQ-5D-5L has Spanish population validity/norms; EQ-5D-5L index scores will be derived using the Spanish EQ-5D-5L value set.[16–21]

#### **THIRD QUESTION**

Is the presence of comorbidities like hormonal abnormalities, respiratory illnesses profile of the samples going to be assessed?

**Response**

Thank you very much for raising this important point.

We would like to clarify that, during the screening process, we systematically collect detailed information on all relevant comorbidities, including hormonal abnormalities (e.g., thyroid disorders), respiratory diseases, and other medical conditions. These data are recorded as part of the baseline clinical profile of each participant to ensure a comprehensive characterisation of the sample and to account for potential confounding factors where appropriate.

#### **FOURTH QUESTION**

Did you consider concurrent rheumatological disease, such as rheumatoidarthritis or osteoarthritis, as exclusion criteria?

**Response**

Thank you for this thoughtful question. You are right that, in the submitted version, this exclusion criterion was not stated with sufficient clarity in the “Participants: Recruitment and eligibility criteria” section.

In our protocol, concurrent rheumatological diseases, such as rheumatoid arthritis and osteoarthritis, are part of the exclusion criteria. We adopted this criterion to avoid clinical and statistical confounding, because inflammatory and degenerative rheumatological conditions can independently drive pain, stiffness, fatigue, functional limitation, and health-related quality of life. These domains overlap substantially with our primary and secondary outcomes in fibromyalgia and could obscure intervention effects.

In addition, we sought to improve diagnostic specificity and sample homogeneity. Contemporary fibromyalgia diagnostic approaches are designed to distinguish fibromyalgia from other rheumatologic conditions such as rheumatoid arthritis and osteoarthritis, supporting the need to minimise diagnostic overlap in efficacy trials.

Finally, we aimed to maintain safety and interpretability of the exercise response. Active inflammatory disease, osteoarthritis flares, or related treatments, including corticosteroids, disease-modifying antirheumatic drugs, or analgesic escalation, could alter exercise tolerance and symptom trajectories during follow-up, complicating attribution of changes to the intervention. This approach is consistent with common practice in fibromyalgia exercise and technology trials, where inflammatory rheumatic disease is frequently excluded to reduce confounding and improve internal validity [14,22–25].

We have therefore amended the manuscript to make this explicit. It now reads as follows:

“Participants will be excluded if they meet any of the following criteria:
(1) presence of comorbidities and/or symptoms that constitute a contraindication for FIVR and exercise-based interventions, including: (i) FIVR-related: history of epilepsy or photosensitive seizures; severe vestibular disorders/vertigo or marked susceptibility to motion sickness/cybersickness; acute symptoms increasing the risk of adverse VR effects at the time of a session (e.g., severe headache/migraine, pronounced dizziness, nausea/vomiting, or disorientation); or severe uncorrected visual impairment preventing safe headset use; and (ii) exercise-related: any unstable or acute cardiopulmonary condition (e.g., unstable angina, uncontrolled arrhythmias, decompensated heart failure, acute myocarditis/pericarditis, acute pulmonary embolism), uncontrolled severe hypertension, or acute systemic illness/fever; (2) presence of medical conditions likely to interfere with outcome assessment or interpretation, such as severe auditory, perceptual, or sensory disorders, or concurrent rheumatological diseases (e.g., rheumatoid arthritis or osteoarthritis);(3) use of medications that are likely to materially interfere with the assessment or interpretation of study outcomes;(4) (i) engagement in another therapeutic physical activity programme during the intervention period; and or (ii) initiation of new therapies or substantial modifications to existing therapies(including medication or physical therapy) during the study period, which could materially affect study outcomes; (5) inability to attend the centre where the interventions will take place.”

#### **FIFTH QUESTION**

What are the parts of the device to be used in the VR treatment?

**Response**

Thank you for your follow-up question regarding the technical specifications of the system. To provide further clarity for the manuscript, the following describes the specific components of the device and how they facilitate the therapeutic intervention:

The treatment was administered using a fully immersive virtual reality (FIVR) system specifically configured for the manipulation of visual-propioceptive feedback. The device consists of three primary integrated layers:

1. Hardware and Kinematic Capture System

- Head-Mounted Display (HMD): An HTC Vive Pro headset was used to deliver the fully immersive visual environment, effectively isolating the patient from external visual cues and replacing them with a computer-generated reality.
- Motion Trackers: Three HTC Vive Trackers were utilized to capture real-time movement data. These were secured using specialized straps to the patient's waist (L3-L4 level) and both ankles/feet.
- Base Stations (Lighthouses): Two external laser emitters were positioned to track the sensors and the HMD within a 360-degree therapeutic space, ensuring high-precision movement registration.

2. Software and Movement Manipulation (The "Visual Gain" Engine)

- Development Platform: The virtual environment (a simulated gym with a front-facing mirror) was developed using the Unity 5.3.1 engine.
- Avatar Representation: The system generates a full-body kinematic avatar that the patient perceives in the first person, enhancing the sense of embodiment within the fully immersive experience.
- Gain Manipulation Algorithm: This is the core functional component of the treatment. The software is programmed to apply a "Visual Gain" factor (e.g., 0.8) to the lumbar flexion and extension axes. In this setup, the avatar is intentionally programmed to move less than the patient’s actual physical movement during both forward and backward bending. By providing this subestimated visual feedback, the system encourages the patient to increase their range of motion (ROM) to reach a visual goal, effectively bypassing kinesiophobic barriers in both directions of movement.

3. Processing Unit

- All data integration and real-time rendering were managed by a high-performance MSI Raider GE78 HX laptop. This ensured that the intentional asynchrony remained fluid and did not cause sensory conflict or motion sickness, which is essential for maintaining the integrity of the fully immersive experience.

#### **SIXTH QUESTION**

What types of videos or games are going to be used for VR treatment?

**Response**

Thank you for your inquiry regarding the nature of the visual content used in the intervention. We would like to note, respectfully, that the detailed description of the VR content (including the materials and access links provided below) is already available in the Supplementary Materials, specifically in S3 Appendix (Intervention manual).

We would like to clarify that the treatment does not utilize commercial videos or standard recreational games. Instead, it employs a fully immersive virtual reality environment custom-developed using the Unity 5.3.1 engine.

The therapeutic process is based on the embodiment of the patient within a first-person kinematic avatar. Rather than being a passive observer, the patient performs a structured program of 10 therapeutic exercises focused on lumbopelvic stability, strength, and mobility directly through the virtual world. By observing their movements in a virtual mirror in real-time, the sense of embodiment is significantly enhanced, which is crucial for the success of the intervention.

A key feature of this system is the integrated Visual Gain algorithm. This software allows for the intentional subestimation of the patient’s physical range of motion, displaying 20 percent less flexion and 10 percent less extension than the actual movement performed. By interacting with the virtual world under these manipulated conditions, patients are encouraged to increase their physical activity beyond their perceived limitations, effectively reducing kinesiophobia and facilitating graded exposure.

To provide a more comprehensive view of the clinical application, we have provided access to a detailed intervention protocol document at the following link [[S3 Appendix. Intervention manual](https://ceu365-my.sharepoint.com/:w:/g/personal/carlos_salvador1_alumnos_uchceu_es/IQBGduBZfRWxRZgygg8UJN8cAQAb0q3NKYsidLNO7PCo99E)] and a demonstration video available at [<https://drive.google.com/file/d/1bszDrgOKrwQzkJtK3Di_bNA8g0HGn7iV/view?usp=sharing>] where the system’s functionality and the patient’s interaction with the virtual environment can be observed.

We hope this information adequately addresses your question. Please let us know if you require any further clarification.

#### **SEVENTH QUESTION**

What are the operational definitions of the “physiological, psychological, and physical outcomes” of fibromyalgia?

**Response**

We appreciate the opportunity to clarify this point. The terms “physiological, psychological, and physical outcomes” were used in the final paragraph of the Introduction as a concise way to summarise the range of secondary outcomes included in the trial, rather than to introduce undefined constructs. Our intention was to avoid listing each individual variable in that section and instead group them conceptually.

Specifically, these operational definitions correspond to the following measures assessed in the study:

- Psychological outcomes: Hospital Anxiety and Depression Scale (HADS), Pain Catastrophizing Scale (PCS), Fear-Avoidance Beliefs Questionnaire (FABQ), Tampa Scale for Kinesiophobia (TSK-11), Behavioural Regulation in Exercise Questionnaire-3 (BREQ-3), and the psychological dimension of the EQ-5D-5L.
- Physiological outcomes: Central Sensitization Inventory (CSI), Quantitative Sensory Testing (QST), Spanish FreBAQ-S (body perception), Fibromyalgia Survey Diagnostic Criteria (FSDC; symptom severity components), and selected domains related to fatigue and sleep (Multidimensional Fatigue Inventory [MFI-20] and Pittsburgh Sleep Quality Index [PSQI]).
- Physical outcomes: 30-second Sit-to-Stand test (STS-30), Timed Up and Go (TUG), isometric quadriceps strength, handgrip strength, lumbar flexion and extension range of motion (including the influence of modified visual feedback), and physical activity level (IPAQ-E).

Therefore, the terminology was used as a structured way to categorise the predefined secondary outcomes. To improve clarity, we are happy to revise the manuscript by explicitly linking these categories to the corresponding validated instruments in the text.

#### **EIGHTH QUESTION**

Why do you want to include only those patients with a pain intensity score of ≥3?

**Response**

We selected a minimum pain intensity score of ≥3 (on a 0–10 scale) to ensure that participants present at least a clinically relevant level of pain at baseline, thereby avoiding floor effects and enhancing the ability to detect meaningful changes following the intervention. Including individuals with very low baseline pain could limit responsiveness and reduce the sensitivity of the analysis, as small absolute changes may not reflect clinically meaningful improvement.

A threshold of ≥3 is commonly considered indicative of at least mild-to-moderate pain and has been used in clinical research to ensure that participants experience persistent symptoms warranting intervention. This approach improves internal validity by ensuring that the sample has sufficient symptom burden for the intervention to demonstrate potential efficacy, while still maintaining external validity by not restricting inclusion to only severe cases[26].

#### **NINTH QUESTION**

What are the comorbidities and/or symptoms that you will consider as contraindications for IVR and exercise-based interventions?

**Response**

We thank the reviewer for this important question. We have revised the exclusion criteria to explicitly define the comorbidities and/or symptoms considered contraindications for both the FIVR exposure and the exercise component. Specifically, we now distinguish FIVR-related contraindications (e.g., epilepsy/photosensitive seizures, severe vestibular disorders/marked cybersickness susceptibility, severe uncorrected visual impairment preventing safe headset use, and acute symptoms at the time of a session such as severe migraine, pronounced dizziness, nausea/vomiting or disorientation) from exercise-related contraindications (e.g., unstable or acute cardiopulmonary conditions, uncontrolled severe hypertension, or acute systemic illness/fever). We also clarify that contraindications are assessed at screening and re-checked immediately prior to each session, with temporary contraindications leading to session deferral when applicable. Accordingly, the revised manuscript text now reads as follows:

“Participants will be excluded if they meet any of the following criteria:(1) presence of comorbidities and/or symptoms that constitute a contraindication for FIVR and exercise-based interventions, including: (i) FIVR-related: history of epilepsy or photosensitive seizures; severe vestibular disorders/vertigo or marked susceptibility to motion sickness/cybersickness; acute symptoms increasing the risk of adverse VR effects at the time of a session (e.g., severe headache/migraine, pronounced dizziness, nausea/vomiting, or disorientation); or severe uncorrected visual impairment preventing safe headset use; and (ii) exercise-related: any unstable or acute cardiopulmonary condition (e.g., unstable angina, uncontrolled arrhythmias, decompensated heart failure, acute myocarditis/pericarditis, acute pulmonary embolism), uncontrolled severe hypertension, or acute systemic illness/fever; (2) presence of medical conditions likely to interfere with outcome assessment or interpretation, such as severe auditory, perceptual, or sensory disorders, or concurrent rheumatological diseases (e.g., rheumatoid arthritis or osteoarthritis);(3) use of medications that could potentially affect study results; (4) engagement in another therapeutic physical activity programme during the intervention period, or any modifications to existing therapies (including medication or physical therapy) throughout the study; (5) inability to attend the centre where the interventions will take place.”

#### **TENTH QUESTION**

Why did you select 6 weeks for the duration of therapy? And how did you determine the 2 times per week frequency of sessions? And why did you select 60 minutes for the duration of a session?

**Response**

We selected a 6-week intervention with two supervised sessions per week and 60 minutes per session for three converging reasons: (i) this dose is consistent with prior exercise and IVR-based exercise protocols in fibromyalgia, (ii) it aligns with the most common and feasible dosing used in fibromyalgia exercise trials and evidence syntheses, and (iii) it balances therapeutic exposure with fatigue, symptom fluctuation, and adherence constraints typical of fibromyalgia. In addition, based on previous studies conducted by our research group in people with chronic low back pain using the same immersive VR treatment, we observed that this dosing was highly effective, supporting its selection for the present trial.

**Why 6 weeks?**

A 6-week programme is a commonly used “short-course” timeframe in fibromyalgia rehabilitation trials that is long enough to produce clinically meaningful changes while limiting attrition and symptom-burden-related drop-out. Importantly for our intervention type, recent fibromyalgia work specifically using immersive VR–based exercise has used a 6-week programme and reported feasibility/safety and improvements in patient-relevant outcomes[27,28]. Additionally, controlled exercise programmes in fibromyalgia frequently span 6–24 weeks, with measurable benefits observed within this range[29].

**Why 2 sessions/week?**

Across fibromyalgia exercise RCTs and reviews, 2–3 sessions/week is the most typical supervised frequency and is widely considered feasible for this population. For example, a Cochrane review of aerobic exercise trials in fibromyalgia reported that programmes were delivered, on average, 2–3 times/week[29]. A fibromyalgia telerehabilitation trial also used two sessions/week at moderate intensity with good feasibility[30]. We therefore chose 2 sessions/week as a pragmatic, evidence-consistent frequency that provides repeated exposure while limiting exacerbation risk and burden for participants with high fatigue and symptom variability.

**Why 60 minutes/session?**

A 60-minute session length is standard in many fibromyalgia supervised exercise interventions because it accommodates a complete structure (warm-up, main therapeutic content, and cool-down), while allowing progression and adequate rest. In fibromyalgia exercise trials, sessions commonly fall in the 45–60 min range (including supervised programmes delivered twice weekly)[31]. Recent dose-focused evidence syntheses also identify protocols with ~60 min/session as part of effective dosing patterns in fibromyalgia[32].

#### **ELEVENTH QUESTION**

Why did you choose HTC Vive Pro?

**Response**

Thank you for your question regarding the selection of the HTC Vive Pro for this study. The primary reason for this choice was that our therapeutic software was custom-developed and specifically optimized for the architecture of this hardware. This optimization ensured high tracking fidelity and the stability required to implement visual gain manipulations smoothly, preventing latencies or sensory discrepancies that could affect the patient’s experience.

Furthermore, the development of this virtual reality system was made possible through a research project funded by the Ministry of Science and Innovation of the Government of Spain. This support allowed us to design a robust technical infrastructure specifically oriented toward meeting the high immersion requirements necessary for these investigations and subsequent clinical trials.

It is also important to note that our research team is currently working on migrating the intervention to Meta Quest devices. The objective of this transition is to enhance the clinical scalability of the treatment, allowing the model to evolve from individual therapy to a group-based approach. We are developing a system for synchronized sessions that will allow for the treatment of up to five patients per hour simultaneously, thereby optimizing clinical resources without compromising the effectiveness of the personalized intervention.

We hope this clarifies the technical and strategic reasons behind our hardware selection. We remain at your disposal for any further information.

#### **TWELVTH QUESTION**

Why did you choose 20% arm underestimation during flexion and 10% underestimation during extension specifically?

**Response**

The specific selection of a 20% underestimation for flexion and 10% for extension was based on the results of previous psychophysical validation studies (Pilot Study) conducted by our research group. The objective of these preliminary studies was to determine the perception thresholds at which visual gain manipulations remain imperceptible to the user, thereby maintaining the integrity of the immersive illusion.

Regarding lumbar flexion, the data indicated that a gain of 0.8 (equivalent to a 20% underestimation) is the maximum value at which the avatar's movement is perceived as natural and non-manipulated by the majority of subjects. For extension movements, it was observed that individuals exhibit greater proprioceptive and sensory sensitivity in this plane; therefore, a gain of 0.9 (10% underestimation) was selected to prevent conscious detection of the visual discrepancy and to ensure safety during the exercise.

These specific parameters have been documented and validated in previous publications derived from this research line, ensuring that the manipulation is significant enough to encourage a greater actual range of motion and reduce kinesiophobia, yet subtle enough not to disrupt the sense of embodiment within the virtual environment[33,34].

We hope this technical justification clarifies the methodological criteria used in our study.

# **BIBLIOGRAPHY**

1.	Bennett RM, Bushmakin AG, Cappelleri JC, Zlateva G, Sadosky AB. Minimal clinically important difference in the fibromyalgia impact questionnaire. Journal of Rheumatology. 2009;36: 1304–1311. doi:10.3899/jrheum.081090

2.	Pérez-Aranda A, Andrés-Rodríguez L, Feliu-Soler A, Núñez C, Stephan-Otto C, Pastor-Mira MA, et al. Clustering a large Spanish sample of patients with fibromyalgia using the Fibromyalgia Impact Questionnaire-Revised: Differences in clinical outcomes, economic costs, inflammatory markers, and gray matter volumes. Pain. 2019;160: 908–921. doi:10.1097/j.pain.0000000000001468

3.	Häuser W, Sarzi-Puttini P, Fitzcharles M-A. Fibromyalgia syndrome: under-, over- and misdiagnosis. Clin Exp Rheumatol. 2019;37 Suppl 116: 90–97.

4.	Clauw DJ. Fibromyalgia. JAMA. 2014;311: 1547. doi:10.1001/jama.2014.3266

5.	Cabo-Meseguer A, Cerdá-Olmedo G, Trillo-Mata JL. Fibromialgia: prevalencia, perfiles epidemiológicos y costes económicos. Med Clin (Barc). 2017;149: 441–448. doi:10.1016/j.medcli.2017.06.008

6.	Robinson RL, Birnbaum HG, Morley MA, Sisitsky T, Greenberg PE, Claxton AJ. Economic cost and epidemiological characteristics of patients with fibromyalgia claims. J Rheumatol. 2003;30: 1318–25.

7.	Wright CL, Mist SD, Ross RL, Jones KD. Duloxetine for the treatment of fibromyalgia. Expert Rev Clin Immunol. 2010;6: 745–756. doi:10.1586/eci.10.64

8.	Guymer EK, Littlejohn GO, Brand CK, Kwiatek RA. Fibromyalgia onset has a high impact on work ability in Australians. Intern Med J. 2016;46: 1069–1074. doi:10.1111/imj.13135

9.	Wolfe F, Smythe HA, Yunus MB, Bennett RM, Bombardier C, Goldenberg DL, et al. The american college of rheumatology 1990 criteria for the classification of fibromyalgia. Arthritis Rheum. 1990;33: 160–172. doi:10.1002/art.1780330203

10.	Wolfe F, Clauw DJ, Fitzcharles M, Goldenberg DL, Katz RS, Mease P, et al. The American College of Rheumatology Preliminary Diagnostic Criteria for Fibromyalgia and Measurement of Symptom Severity. Arthritis Care Res (Hoboken). 2010;62: 600–610. doi:10.1002/acr.20140

11.	WOLFE F, CLAUW DJ, FITZCHARLES M-A, GOLDENBERG DL, HÄUSER W, KATZ RS, et al. Fibromyalgia Criteria and Severity Scales for Clinical and Epidemiological Studies: A Modification of the ACR Preliminary Diagnostic Criteria for Fibromyalgia. J Rheumatol. 2011;38: 1113–1122. doi:10.3899/jrheum.100594

12.	Wolfe F, Clauw DJ, Fitzcharles M-A, Goldenberg DL, Häuser W, Katz RL, et al. 2016 Revisions to the 2010/2011 fibromyalgia diagnostic criteria. Semin Arthritis Rheum. 2016;46: 319–329. doi:10.1016/j.semarthrit.2016.08.012

13.	Galvez-Sánchez CM, Reyes del Paso GA. Diagnostic Criteria for Fibromyalgia: Critical Review and Future Perspectives. J Clin Med. 2020;9: 1219. doi:10.3390/jcm9041219

14.	Wang C, Schmid CH, Fielding RA, Harvey WF, Reid KF, Price LL, et al. Effect of tai chi versus aerobic exercise for fibromyalgia: comparative effectiveness randomized controlled trial. BMJ. 2018; k851. doi:10.1136/bmj.k851

15.	Gulsen C, Soke F, Eldemir K, Apaydin Y, Ozkul C, Guclu-Gunduz A, et al. Effect of fully immersive virtual reality treatment combined with exercise in fibromyalgia patients: a randomized controlled trial. Assistive Technology. 2022;34: 256–263. doi:10.1080/10400435.2020.1772900

16.	Carvajal-Parodi C, Arias-Álvarez G, Ulloa-Díaz D, Romero-Vera L, Andrades-Ramírez O, Guede-Rojas F, et al. Immersive Virtual Reality-Based Exercise for Pain Management in Fibromyalgia: An Exploratory Study with Risk of Poor Outcomes Stratification. Applied Sciences. 2025;15: 5956. doi:10.3390/app15115956

17.	Salgueiro M, García-Leiva JM, Ballesteros J, Hidalgo J, Molina R, Calandre EP. Validation of a Spanish version of the Revised Fibromyalgia Impact Questionnaire (FIQR). Health Qual Life Outcomes. 2013;11: 132. doi:10.1186/1477-7525-11-132

18.	Cuesta-Vargas AI, Roldan-Jimenez C, Neblett R, Gatchel RJ. Cross-cultural adaptation and validity of the Spanish central sensitization inventory. Springerplus. 2016;5: 1837. doi:10.1186/s40064-016-3515-4

19.	Munguía-Izquierdo D, Segura-Jiménez V, Camiletti-Moirón D, Pulido-Martos M, Alvarez-Gallardo IC, Romero A, et al. Multidimensional Fatigue Inventory: Spanish adaptation and psychometric properties for fibromyalgia patients. The Al-Andalus study. Clin Exp Rheumatol. 2012;30: 94–102.

20.	Hita-Contreras F, Martínez-López E, Latorre-Román PA, Garrido F, Santos MA, Martínez-Amat A. Reliability and validity of the Spanish version of the Pittsburgh Sleep Quality Index (PSQI) in patients with fibromyalgia. Rheumatol Int. 2014;34: 929–936. doi:10.1007/s00296-014-2960-z

21.	Hernandez G, Garin O, Pardo Y, Vilagut G, Pont À, Suárez M, et al. Validity of the EQ–5D–5L and reference norms for the Spanish population. Quality of Life Research. 2018;27: 2337–2348. doi:10.1007/s11136-018-1877-5

22.	Ramos-Goñi JM, Craig BM, Oppe M, Ramallo-Fariña Y, Pinto-Prades JL, Luo N, et al. Handling Data Quality Issues to Estimate the Spanish EQ-5D-5L Value Set Using a Hybrid Interval Regression Approach. Value in Health. 2018;21: 596–604. doi:10.1016/j.jval.2017.10.023

23.	Polat M, Kahveci A, Muci B, Günendi Z, Kaymak Karataş G. The Effect of Virtual Reality Exercises on Pain, Functionality, Cardiopulmonary Capacity, and Quality of Life in Fibromyalgia Syndrome: A Randomized Controlled Study. Games Health J. 2021;10: 165–173. doi:10.1089/g4h.2020.0162

24.	Chittaro L, Longhino S, Serafini M, Cacioppo S, Quartuccio L. Efficacy of Immersive Virtual Reality Combined With Multisensor Biofeedback on Chronic Pain in Fibromyalgia: A Pilot Randomized Controlled Trial. ACR Open Rheumatol. 2025;7: e70048. doi:10.1002/acr2.70048

25.	Sañudo B, de Hoyo M, Carrasco L, McVeigh JG, Corral J, Cabeza R, et al. The effect of 6-week exercise programme and whole body vibration on strength and quality of life in women with fibromyalgia: a randomised study. Clin Exp Rheumatol. 2010;28: S40-5.

26.	Tomas-Carus P, Biehl-Printes C, del Pozo-Cruz J, Parraca JA, Folgado H, Pérez-Sousa MÁ. Effects of respiratory muscle training on respiratory efficiency and health-related quality of life in sedentary women with fibromyalgia: a randomised controlled trial. Clin Exp Rheumatol. 2021. doi:10.55563/clinexprheumatol/0v55nh

27.	Kamerman PR, Vollert J. Greater baseline pain inclusion criteria in clinical trials increase regression to the mean effect: a modelling study. Pain. 2022;163: e748–e758. doi:10.1097/j.pain.0000000000002468

28.	Arias-Álvarez G, Santamera-Lastras M, Guzmán-Oyarzo D, Osorio-Torres W, Parada-Norambuena B, Pecos-Martín D, et al. Immersive Virtual Reality Exercise: Effects on Cortisol, Quality of Life, Cognitive Function, and Psychological Symptoms in Fibromyalgia. Medicina (B Aires). 2026;62: 446. doi:10.3390/medicina62030446

29.	ARIAS-ÁLVAREZ G, GUZMÁN-PINCHEIRA C, GONZÁLEZ-GONZÁLEZ D, OSORIO-TORRES W, PECOS-MARTÍN D, GÓMEZ-PULIDO J, et al. Effects of immersive virtual reality-based exercise on quality of life, stress, anxiety, depression, and handgrip strength in fibromyalgia: A pilot study. Virtual Reality & Intelligent Hardware. 2025;7: 267–278. doi:10.1016/j.vrih.2025.03.004

30.	Bidonde J, Busch AJ, Schachter CL, Overend TJ, Kim SY, Góes SM, et al. Aerobic exercise training for adults with fibromyalgia. Cochrane Database Syst Rev. 2017;6: CD012700. doi:10.1002/14651858.CD012700

31.	Hernando-Garijo I, Medrano-de-la-Fuente R, Jiménez-del-Barrio S, Mingo-Gómez MT, Hernández-Lázaro H, Lahuerta-Martin S, et al. Effects of a Telerehabilitation Program in Women with Fibromyalgia at 6-Month Follow-Up: Secondary Analysis of a Randomized Clinical Trial. Biomedicines. 2022;10: 3024. doi:10.3390/biomedicines10123024

32.	Sañudo B, Galiano D, Carrasco L, Blagojevic M, de Hoyo M, Saxton J. Aerobic Exercise Versus Combined Exercise Therapy in Women With Fibromyalgia Syndrome: A Randomized Controlled Trial. Arch Phys Med Rehabil. 2010;91: 1838–1843. doi:10.1016/j.apmr.2010.09.006

33.	Li Y, Yuan Y, Hua L, Ran L, Qiu B, Yang Y, et al. Optimal aerobic exercise dose for pain relief in fibromyalgia syndrome: a systematic review and meta-analysis. Front Physiol. 2025;16. doi:10.3389/fphys.2025.1693735

34.	Jordán-López J, Arguisuelas MD, Doménech J, Peñalver-Barrios ML, Miragall M, Herrero R, et al. Modifying lumbar flexion pain thresholds in patients with chronic low back pain through visual-proprioceptive manipulation with virtual reality: a cross-sectional study. J Neuroeng Rehabil. 2025;22: 138. doi:10.1186/s12984-025-01664-2

35.	Jordán-López J, Arguisuelas MD, Doménech J, Peñalver-Barrios ML, Miragall M, Herrero R, et al. Visual feedback manipulation in virtual reality alters movement-evoked pain perception in chronic low back pain. Sci Rep. 2025;15: 20372. doi:10.1038/s41598-025-08094-z
